# The Association of Long COVID and CKD: Findings from the National Clinical Cohort Collaborative

**DOI:** 10.2215/CJN.0000000773

**Published:** 2025-08-05

**Authors:** A. Jerrod Anzalone, Spencer Krichevsky, Yun Jae Yoo, Kenneth J. Wilkins, Fadhl Alakwaa, Feifan Liu, Ankit Sakhuja, Joel H. Saltz, Yun Han, Richard L. Zhu, Soko Setoguchi, Sandra L. Kane-Gill, Sandeep K. Mallipattu, Yongqun He, David H. Ellison, James Brian Byrd, Chirag R. Parikh, Rajiv Saran, Richard A. Moffitt, Farrukh M. Koraishy

**Affiliations:** 1 Department of Biostatistics, College of Public Health, University of Nebraska Medical Center, Omaha, Nebraska; 2 Department of Biomedical Informatics, Stony Brook University, Stony Brook, New York; 3 Department of Hematology and Medical Oncology, Emory University, Atlanta, Georgia; 4 Biostatistics Program, Department of Preventive Medicine and Biostatistics, National Institute of Diabetes and Digestive and Kidney Diseases, F. Edward Hébert School of Medicine, Uniformed Services University of the Health Sciences, Bethesda, Maryland; 5 Nephrology Division, Department of Internal Medicine, University of Michigan, Ann Arbor, Michigan; 6 Department of Population and Quantitative Health Sciences, University of Massachusetts Chan Medical School, Worcester, Massachusetts; 7 Division of Data Driven and Digital Medicine, Department of Medicine, Institute for Critical Care Medicine, Icahn School of Medicine at Mount Sinai, New York, New York; 8 Institution for Clinical and Translational Research, Johns Hopkins University School of Medicine, Baltimore, Maryland; 9 Department of Medicine and Epidemiology, Rutgers Robert Wood Johnson Medical School and School of Public Health, New Brunswick, New Jersy; 10 Department of Pharmacy and Therapeutics, School of Pharmacy, University of Pittsburgh, Pittsburgh, Pennsylvania; 11 Division of Nephrology and Hypertension, Department of Medicine, Stony Brook University, Stony Brook, New York; 12 Northport VAMC, Northport, New York; 13 Department of Microbiology and Immunology, University of Michigan Medical School, Ann Arbor, Michigan; 14 Oregon Clinical and Translational Research Institute, Oregon Health and Science University, Portland, Oregon; 15 Division of Cardiovascular Medicine, Department of Medicine, University of Michigan, Ann Arbor, Michigan; 16 Division of Nephrology, Department of Medicine, Johns Hopkins School of Medicine, Baltimore, Maryland; 17 Division of Nephrology, Departments of Internal Medicine and Epidemiology, University of Michigan, Ann Arbor, Michigan

**Keywords:** CKD, COVID-19

## Abstract

**Key Points:**

- Baseline CKD, even mild, is associated with a higher risk of long coronavirus disease (COVID) in patients with acute severe acute respiratory syndrome coronavirus infection.
- Among those without CKD at baseline, Long COVID is associated with a higher risk of developing new CKD and faster kidney function decline.
- Associations between Long COVID and CKD/kidney function decline persist after matching, adjustment, and accounting for the competing risk of death.

**Background:**

Among patients with acute coronavirus disease-19 (COVID-19), the association of CKD and Long COVID has not been reported in large multicenter cohorts.

**Methods:**

This study used data from 59 health care systems across the United States, in the National Clinical Cohort Collaborative COVID enclave, to analyze the relationship between CKD and Long COVID among adults diagnosed with acute COVID-19 between October 2021 and September 2023. We conducted two main analyses. *First analysis*: we tested if baseline CKD (eGFR <60 ml/min per 1.73 m^2^ or diagnostic code) or baseline ESKD are risk factors for Long COVID (identified using ICD-10-CM code U09.9). We secondarily assessed associations between baseline mild CKD (Stage 3a, eGFR 45–59 ml/min per 1.73 m^2^) and Long COVID. *Second Analysis*: among patients without baseline CKD/ESKD, we examined if incident CKD/ESKD and eGFR decline (≥20% in 1 year) were associated with Long COVID. We used propensity score matching for demographics and data contributing site, with models adjusted for risk factors and competing risk of death. All outcomes were evaluated within a 365-day follow-up period from the onset of acute COVID-19.

**Results:**

*First analysis*: From an unmatched cohort of 2,385,20 patients with acute COVID-19, those with baseline CKD/ESKD had a higher risk of Long COVID (adjusted subdistribution hazard ratio [sHR], 1.13; 95% confidence interval [CI], 1.09 to 1.18) after matching. A similar risk was noted even among those with mild CKD (sHR, 1.15; 95% CI, 1.05 to 1.25). *Second Analysis:* Among patients with acute COVID-19 and without baseline CKD/ESKD, Long COVID was associated with incident CKD/ESKD (sHR, 1.65; 95% CI, 1.51 to 1.81) and 20% or greater eGFR decline (sHR, 1.21; 95% CI 1.04 to 1.40) within 1 year.

**Conclusions:**

CKD, even mild, was associated with an higher risk of Long COVID. Among those without baseline CKD, Long COVID was associated with incident CKD and eGFR decline.

## Visual Abstract

**Figure:**
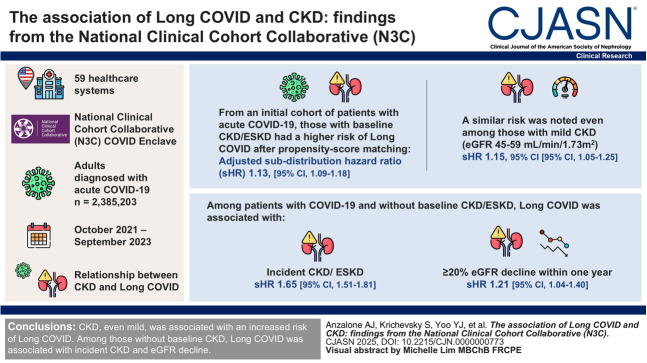

## Introduction

Long coronavirus disease (COVID; also referred to as “post-COVID syndrome” or “Post-Acute Sequelae of SARS-CoV-2 infection [PASC]”) is the persistence or development of new symptoms after the initial severe acute respiratory syndrome coronavirus (SARS-CoV-2) infection.^1^ Among patients with acute coronavirus disease 2019 (COVID-19), the prevalence of Long COVID ranges between 6% and 15%.^2,3^ The pathogenesis of Long COVID has been attributed to viral strain, immune response, and inflammation.^4^ Female sex,^3,5,6^ race and ethnicity,^7^ smoking,^6^ and preexisting comorbid conditions such as cognitive disorders,^2^ psychiatric conditions,^5,6,8,9^ and medical comorbidities^2,6,7,10–12^ have been associated with an increased risk of Long COVID. On the other hand, COVID-19 vaccination (compared with no vaccination) is associated with a lower risk.^6,13^

The studies that included CKD and ESKD as a risk factor for Long COVID in their analyses have shown variable results.^6,7,9,14–19^ These studies were limited by sample size, duration of follow-up, and focused populations. Several symptoms associated with Long COVID,^11,20^ including malaise, fatigue, gastrointestinal symptoms, palpitations, sexual dysfunction, dyspnea, and chest pain, are often also associated with advanced CKD and ESKD.^21^ Mild, early CKD, which is typically asymptomatic, has not been evaluated as a risk factor for Long COVID. In the first part of our study, we sought to test the hypothesis that baseline CKD/ESKD and even mild CKD (Stage 3a with eGFR in the range 45–59 ml/min per 1.73 m^2^) is associated with increased risk of Long COVID in a large nationally representative population.

Acute COVID-19 in hospitalized patients is associated with a high incidence of AKI^22,23^ and eGFR decline after hospital discharge.^22,24,25^ Even among patients without clinical AKI, a risk of CKD has been reported.^26^ Although Long COVID diagnosis is typically made on the basis of the duration of a constellation of symptoms,^20^ chronic organ injury and dysfunction have now been recognized as a manifestation of PASC.^22,27–29^ In a study of US Veterans, COVID-19 survivors were reported to have an higher risk of AKI, eGFR decline, and ESKD during the postacute phase.^30^ Those with Long COVID have a greater risk of cardiac arrhythmias, pulmonary embolism, ischemic stroke, coronary artery disease, heart failure, chronic obstructive pulmonary disease, and asthma compared with patients without COVID-19.^31^ The importance of considering kidney disease as a manifestation of Long COVID has been highlighted, but understudied.^32,33^ In the second part of our study, we investigated the association of incident CKD with Long COVID in patients without baseline CKD. We hypothesize that after acute COVID-19, patients with Long COVID have a greater incidence of CKD and eGFR decline than those without.

## Methods

### Study Environment and Review Board Approvals

This retrospective cohort study used the National Clinical Cohort Collaborative (N3C) COVID Enclave,^34^ a next-generation registry^35^ containing longitudinal electronic health record data (EHR) on SARS-CoV-2-infected patients from US institutions dating back to January 2018.^36^ It contains the largest limited dataset gathered in US history for COVID-19 research.^37^ The N3C Data Access Committee approved this study (RP-68DB83), which adheres to REporting of studies Conducted using Observational Routinely-collected Data reporting guidelines for observational studies.^38^ N3C operates under the authority of the National Institutes of Health (NIH) Institutional Review Board (IRB), with Johns Hopkins University serving as the central IRB (IRB00309495). Each investigator received IRB approval from their respective institutions. No informed consent was obtained from patients because the study used a limited dataset, stripping direct identifiers in compliance with the HIPAA Privacy Rule.

### COVID-19 Definitions

#### Acute COVID-19

All patients were diagnosed with acute COVID-19, defined as a positive PCR, antigen, or diagnosis code.

#### Long COVID

Long COVID was defined using the October 1, 2021, released International Classification of Diseases, Tenth Revision, Clinical Modification (ICD-10-CM) diagnosis code of “Post COVID-19 condition, unspecified” (U09.9). Patients with a long COVID diagnosis 28 days or more after the initial SARS-CoV-2 infection were considered to have Long COVID.^7,39,40^

### Renal Definitions

#### CKD

CKD (both “baseline” and “incident”) was defined by diagnostic codes (Supplemental Methods Table) and/or a single eGFR measurement <60 ml/min per 1.73 m^2^. eGFR was calculated from outpatient-collected serum creatinine measurements according to the 2021 CKD Epidemiology Collaboration equation.^41^ A sensitivity analysis was conducted requiring CKD to defined by two eGFR measurements <60 ml/min per 1.73 m^2^ at least 90 days apart based on KDIGO guidelines.^42^

#### ESKD

ESKD was defined by diagnostic codes (Supplemental Methods Table).

#### GFR Decline

Patients were evaluated for eGFR decline based on the percent difference between their first and last outpatient post-COVID eGFR measurements. Patients with 20% or greater decrease in eGFR in 1 year after acute COVID-19 were deemed to have significant eGFR decline.^43^

### Cohort Design, Inclusion, and Exclusion Criteria

**Figure 1:**
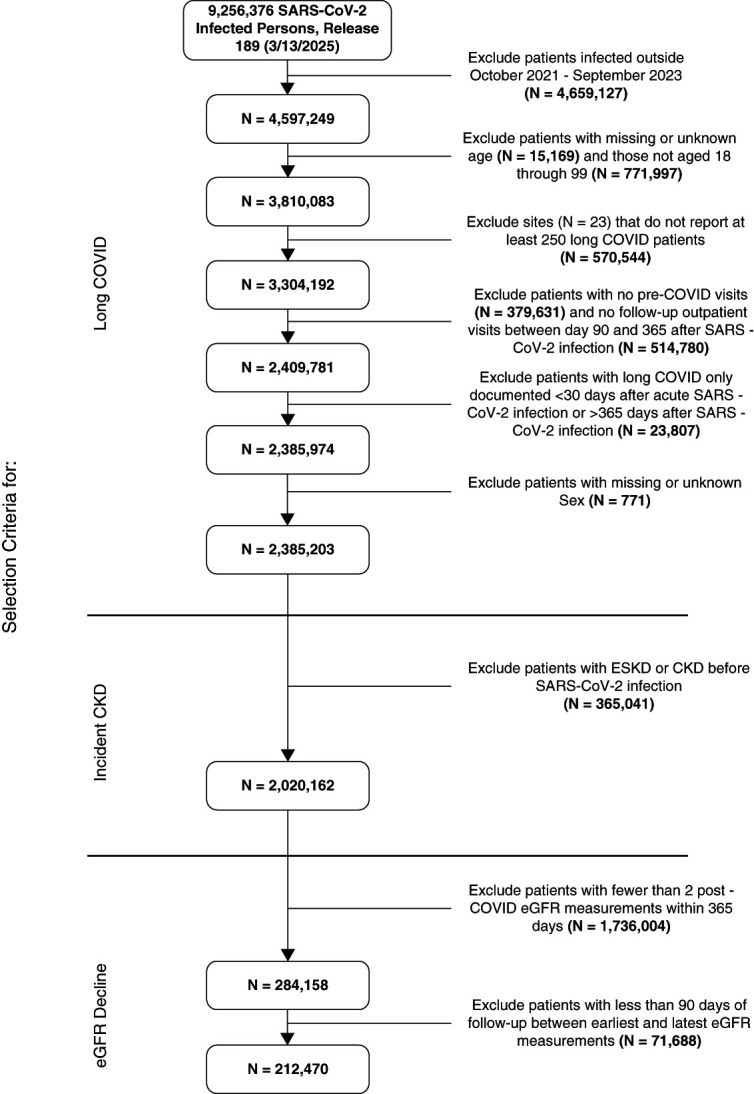
**Analytic Selection Flow Diagram.** Figure 1 shows the three sequentially derived cohorts, each designed to evaluate distinct outcomes. COVID-19, coronavirus disease 2019; SARS-CoV-2, acute respiratory syndrome coronavirus.

Sequentially derived cohorts were used to evaluate distinct outcomes. In the first analysis investigating baseline CKD as a risk factor for Long COVID, we included patients who tested positive for acute COVID-19 between October 1, 2021 (the date the Long COVID ICD-10 code became available in the United States), and September 30, 2023, to allow for a 1-year follow-up period for each patient (Figure 1 and Supplemental Methods Figure). Patients were excluded if age or sex was missing or unknown or if they were younger than 18 or older than 99 years at baseline. We included patients from Long COVID reporting health care systems, with at least 250 patients attributed with U09.9. The criteria of 250 or greater U09.9-coded cases were selected based on precedent within the Researching COVID to Enhance Recovery protocol to reduce misclassification due to underreporting of the U09.9 code set by the 2024 N3C study.^44^ Patients without a health care visit before COVID-19 or without an outpatient clinic visit between 28 and 365 days after acute infection were excluded. We excluded patients with Long COVID diagnosed only before 28 or after 365 days of acute COVID-19. This time frame aligns with the available contemporaneous guidance^39^ and differences in diagnostic practices^7^ throughout our observation window. We then compared those with or without baseline CKD/ESKD for the Long COVID outcome.

In addition, we performed a sensitivity analysis to study patients with baseline CKD Stage 3a (eGFR 45–59 ml/min per 1.73 m^2^) compared with those without any CKD for incident long COVID diagnosis to understand the risk of mild CKD. We excluded patients with ESKD, those without an eGFR measurement in the year before acute COVID-19, and those with a baseline eGFR <45 ml/min per 1.73 m^2^.

In our second analysis, we investigated the association of Long COVID with incident CKD. A refined cohort further excluded patients with any history of CKD or ESKD before their acute COVID-19 diagnosis (Figure 1). In a secondary analysis of eGFR decline, we assessed post-COVID eGFR decline from patients with at least two eGFR measurements between 30 and 365 days post-COVID diagnosis, with a minimum interval of 90 days between the earliest and latest measurements.^42^ eGFR decline was assessed using the earliest post–COVID-19 eGFR measurement recorded after infection (30 days or greater) and the last post–COVID-19 eGFR measurement taken within 365 days.

#### Covariates

The concept sets (diagnoses codes) used to determine all the variables used in the study, including CKD, are detailed in the Supplemental Methods Table.

#### Rurality

Residential zone improvement plan Codes were mapped to Rural-Urban Commuting Area Codes.^45^ Codes 1–3 were classified as urban and 4–10 as rural, which is a definition used by the Federal Office of Rural Health Policy^46,47^ and the Veterans Health Administration Office of Rural Health.^48,49^

#### SARS-CoV-2 Variant-Dominant Periods

SARS-CoV-2 variant-dominant periods based on initial SARS-CoV-2 infection date alignment with the predominant strain, as reported by the US Centers for Disease Control and Prevention.^50,51^ These included Delta (B.1.617.2) before December 17, 2021; Omicron (B.1.1.529, BA.2, BA.2.12.1) between December 18, 2021, and July 1, 2022; and Omicron (BA.5, BQ.1.1, XBB.1.5) between July 2, 2022, and September 30, 2023.

#### Mortality

Mortality data were obtained from N3C-contributing health care systems within 365 days of COVID-19. This encompasses deaths recorded in each health care system's EHR or research data mart.

### Statistical Analyses

Analyses were conducted before and after 1:1 propensity score matching (PSM). Patients were matched by CKD/ESKD status for Long COVID outcomes and by Long COVID status for incident CKD and eGFR decline. Balance between matched groups was assessed using standardized mean differences, with values <0.1 indicating acceptable balance. Baseline characteristics were compared using the Wilcoxon rank-sum test for continuous variables and the Pearson chi-squared test for categorical variables in models before PSM and using standardized mean differences in models after PSM. Time-to-event outcomes were evaluated using Cox proportional hazards models and Fine-Gray subdistribution hazard models to account for death as a competing risk.^52^ Multivariable models adjusted for the following covariates: sex, age group, race and ethnicity, vaccination status, rurality, preexisting comorbidities (heart failure, diabetes mellitus [DM], hypertension, cardiovascular disease [CVD], obesity, tobacco use), and SARS-CoV-2 variant-dominant periods. Missing data were handled through complete case analysis, where patients with missing data in any covariates were excluded from the analysis, apart from race and ethnicity and rurality. We report cause-specific hazard ratios, subdistribution hazard ratios (sHR), cumulative incidence function estimates, and corresponding 95% confidence intervals (CI), in accordance with best practices for competing risk analyses.^53^ *P* values < 0.05 were considered statistically significant (two-sided tests).

### PSM

We generated propensity scores to examine the risk of historical CKD/ESKD on Long COVID incidence and Long COVID attribution on the emergence of incident CKD. The cohorts were matched using a “nearest-neighbor” approach^54^ based on age at COVID-19 diagnosis, sex, race and ethnicity, COVID-19 variant period, and data contributing site. These specific characteristics were chosen for matching to produce a covariate balance on average between groups by using predicted propensity scores. We paired patients with and without Long COVID based on these features by matching on the logit-transformed propensity scores with a caliper of 0.1. We used PSM for age and exact matching for all other covariates. We assessed prematching and postmatching balance using Love plots (see Supplemental Figures 3, 7, and 12).

All analyses and visualizations were conducted within the N3C Enclave using SQL, Python v3.6, and R v4.1.3. The analytic code and underlying data are accessible to researchers with NIH approval.

#### Sensitivity Analyses

To assess the robustness and validity of the primary findings, we performed three sensitivity analyses. For the first sensitivity analysis (alternative CKD definition), we repeated the primary analyses applying a stricter definition of baseline CKD, requiring two eGFR values <60 ml/min per 1.73 m^2^ at least 90 days apart before COVID-19 diagnosis. This stricter definition aligns with KDIGO clinical guidelines for CKD diagnosis and was used to reduce the possibility of misclassification based on single transient eGFR values. For the second sensitivity analysis (adjustment for COVID-19 severity), we expanded the covariate adjustment set to include COVID-19 severity at the initial presentation (categorized as outpatient only, hospitalized without oxygen support, or hospitalized with oxygen support). Because the severity of acute COVID-19 may influence the risk of both Long COVID and renal outcomes, this analysis assessed whether accounting for disease severity materially altered the observed associations. For the third sensitivity analysis 3 (age stratification), we stratified the primary analyses by age group (younger than 65 years and 65 years and older) and re-estimated the associations within each age stratum. Given known age-related differences in susceptibility to Long COVID and CKD, stratification allowed us to examine potential effect modification by age and to ensure that associations were not driven by confounding due to age distribution.

## Results

### Baseline CKD/ESKD and Risk of Incident Long COVID

**Figure 2:**
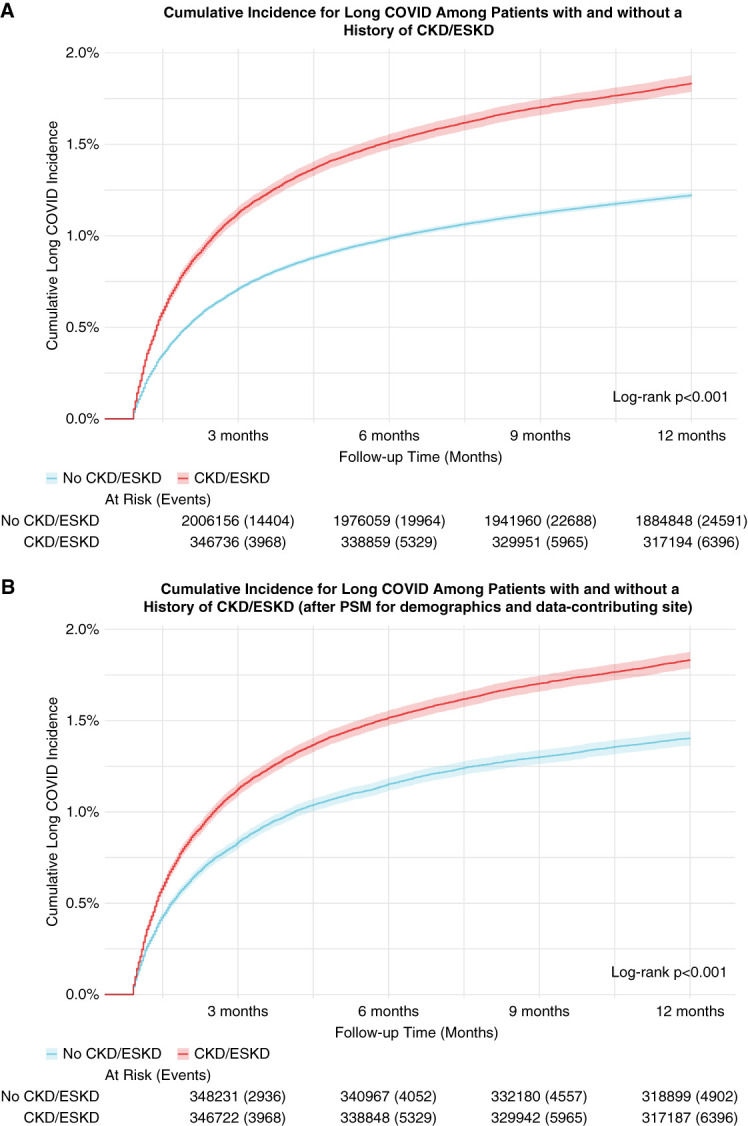
**Cumulative incidence for long COVID.** (A) Cumulative incidence for Long COVID among patients with and without a history of CKD/ESKD. Figure 2A presents cumulative incidence for Long COVID diagnosis from month 1 to month 12 after THE initial SARS-CoV-2 infection for patients with and without a history of CKD/ESKD. (B) Cumulative incidence for Long COVID among patients with and without a history of CKD/ESKD (after PSM for demographics and data contributing site). Figure 2B presents cumulative incidence for Long COVID diagnosis from month 1 to month 12 after the initial SARS-CoV-2 infection for patients with and without a history of CKD/ESKD. COVID, coronavirus disease; PSM, propensity score matching.

In a starting cohort of 2,385,203 patients (Figure 1), compared with patients without CKD/ESKD at the time of acute COVID-19, those with baseline CKD/ESKD (*N*=353,689, 14.8% of the cohort) were more likely to have Long COVID (1.8% versus 1.2%) and die (6.3% versus 0.9%) in the post-COVID period (Figure 2A and Supplemental Figure 1, Supplemental Table 1). Similar findings were found in the PSM cohort (Supplemental Figure 2). Those who had baseline CKD/ESKD (compared with those who did not) were more likely to have Long COVID (1.8% versus 1.4%) and die (6.3% versus 2.7%) in the post-COVID period (Figure 2B, Table 1 and Supplemental Figure 3).

**Table 1:** Descriptive statistics of patients with and without baseline CKD/ESKD after propensity score matching for demographics and data contributing site

Characteristic^a^	Overall, *N*=707,350	No Baseline CKD/ESKD, *n*=353,675	Baseline CKD/ESKD, *n*=353,675	SMD (95% CI)^b^
Age at COVID-19	70 (60–78)	70 (60–77)	70 (60–79)	−0.07 (−0.08 to −0.07)
**Age group**				0.12 (0.12 to 0.13)
18–29	176,539 (25%)	91,060 (26%)	85,479 (24%)	
30–39	7822 (1%)	3943 (1%)	3879 (1%)	
40–49	19,542 (3%)	9828 (3%)	9714 (3%)	
50–59	42,015 (6%)	21,172 (6.0%)	20,843 (6%)	
60–69	97,194 (14%)	49,512 (14%)	47,682 (13%)	
70–79	213,968 (30%)	111,652 (32%)	102,316 (29%)	
80+	150,270 (21%)	66,508 (19%)	83,762 (24%)	
**Sex**				0.00 (0.00 to 0.00)
Female	397,814 (56%)	198,907 (56%)	198,907 (56%)	
Male	309,536 (44%)	154,768 (44%)	154,768 (44%)	
**Race and ethnicity**				0.00 (0.00 to 0.00)
Black or African American non-Hispanic	113,256 (16%)	56,628 (16%)	56,628 (16%)	
Hispanic or Latino any race	42,304 (6%)	21,152 (6%)	21,152 (6%)	
Other non-Hispanic	35,368 (5%)	17,684 (5%)	17,684 (5%)	
Unknown	17,288 (2%)	8644 (2%)	8644 (2%)	
White non-Hispanic	499,134 (71%)	249,567 (71%)	249,567 (71%)	
**Rurality**				0.04 (0.03 to 0.04)
Urban	512,014 (72%)	258,236 (73%)	253,778 (72%)	
Rural	117,088 (17%)	56,176 (16%)	60,912 (17%)	
Missing	*78,248 (11%)*	*39,263 (11%)*	*38,985 (11%)*	
**COVID-19 vaccination status**				0.06 (0.06 to 0.07)
No documented COVID-19 vaccination	387,606 (55%)	199,338 (56%)	188,268 (53%)	
Primary COVID-19 vaccination documented	124,536 (18%)	59,924 (17%)	64,612 (18%)	
Primary and Additional COVID-19 dose(s) documented	195,208 (28%)	94,413 (27%)	100,795 (28%)	
**SARS-CoV-2 variant-dominant period**				0.00 (0.00 to 0.00)
Delta (B.1.617.2)	65,206 (9%)	32,603 (9%)	32,603 (9%)	
Omicron (B.1.1.529, BA.2, BA.2.12.1)	282,580 (40%)	141,290 (40%)	141,290 (40%)	
Omicron (BA.5, BQ.1.1, XBB.1.5)	359,564 (51%)	179,782 (51%)	179,782 (51%)	
Heart failure before COVID-19	120,268 (17%)	27,790 (7.9%)	92,478 (26%)	0.50 (0.50 to 0.51)
CVD before COVID-19	329,002 (47%)	120,279 (34%)	208,723 (59%)	0.52 (0.51 to 0.52)
Hypertension before COVID-19	462,388 (65%)	181,478 (51%)	280,910 (79%)	0.62 (0.61 to 0.62)
Obesity before COVID-19	343,819 (49%)	144,055 (41%)	199,764 (56%)	0.32 (0.31 to 0.32)
Tobacco usage before COVID-19	104,558 (15%)	45,325 (13%)	59,233 (17%)	0.11 (0.11 to 0.12)
Diabetes before COVID-19	214,021 (30%)	68,261 (19%)	145,760 (41%)	0.49 (0.49 to 0.50)
**Baseline eGFR before COVID-19**	70 (54–87)	86 (75–94)	57 (45–70)	1.60 (1.60 to 1.60)
No eGFR available in the yr before COVID-19	*227,253*	*160,763*	*66,490*	
**Adverse post–COVID-19 events**				
Long COVID	11,298 (2%)	4902 (1%)	6396 (2%)	−0.03 (−0.04 to −0.03)
Death after COVID-19 (with no long COVID diagnosis)	31,064 (4%)	9327 (3%)	21,737 (6%)	−0.17 (−0.18 to −0.17)
Any death after COVID-19	31,747 (5%)	9514 (3%)	22,233 (6%)	−0.17 (−0.18 to −0.17)

Table 1 presents descriptive statistics for patients with a documented acute respiratory syndrome coronavirus infection between October 1, 2021, and September 30, 2023, after propensity score matching. Patients were propensity score matching using logistic regression with 1:1 matching for binary long coronavirus status with exact matching on sex, race and ethnicity, coronavirus disease 2019 variant period, and data contributing site and nearest neighbor propensity score matching on age. This includes all patients seen at clinics with ≥250 long coronavirus cases based on study inclusion/exclusion criteria. CI, confidence interval; COVID-19, coronavirus disease 2019; CVD, cardiovascular disease; SARS-CoV-2, acute respiratory syndrome coronavirus; SMD, standardized mean differences.

a *n* (%); median (interquartile range).

b Standardized mean differences with 95% confidence interval.

In univariate Cox regression (Supplemental Table 2), baseline CKD/ESKD was associated with Long COVID, unadjusted HR (1.51; 95% CI, 1.47 to 1.55). This association remained significant in multivariable models after adjusting for sex, age, race and ethnicity, vaccination status, comorbid conditions, rurality, and the competing risk of death (sHR, 1.16; 95% CI, 1.12 to 1.20). Baseline CKD/ESKD remained significantly associated with Long COVID in multivariable models of the PSM cohort (sHR, 1.13; 95% CI, 1.09 to 1.18; Table 2). Other significant risk factors were Delta (B.1.617.2) variant and history of hypertension, CVD, obesity, and tobacco use. Male sex, Black race, vaccination, and Omicron (BA.5, BQ.1.1, XBB.1.5) period were associated with lower risk.

**Table 2:** Univariate and multivariable Cox proportional hazards regression for Long COVID after propensity score matching for demographics and data contributing site

Characteristic	No. Events/*N* (%)	Unadjusted cHR (95% CI)	*P* Value	Adjusted sHR (95% CI)	*P* Value
**CKD/ESKD status**					
No baseline CKD/ESKD	4902/353,675 (1.4%)	Reference		Reference	
Baseline CKD/ESKD	6396/353,675 (1.8%)	1.31 (1.26 to 1.36)	<0.001	1.13 (1.09 to 1.18)	<0.001
**Sex**					
Female	6970/397,814 (1.8%)	Reference		Reference	
Male	4328/309,536 (1.4%)	0.80 (0.77 to 0.83)	<0.001	0.76 (0.73 to 0.79)	<0.001
**Age group**					
60–69	3146/176,539 (1.8%)	Reference		Reference	
18–29	71/7822 (0.91%)	0.51 (0.40 to 0.64)	<0.001	0.54 (0.42 to 0.68)	<0.001
30–39	259/19,542 (1.3%)	0.74 (0.65 to 0.84)	<0.001	0.76 (0.67 to 0.86)	<0.001
40–49	738/42,015 (1.8%)	0.98 (0.91 to 1.07)	0.68	0.98 (0.90 to 1.06)	0.62
50–59	1716/97,194 (1.8%)	0.99 (0.93 to 1.05)	0.74	0.98 (0.92 to 1.04)	0.51
70–79	3428/213,968 (1.6%)	0.90 (0.86 to 0.94)	<0.001	0.91 (0.87 to 0.96)	<0.001
80+	1940/150,270 (1.3%)	0.72 (0.68 to 0.77)	<0.001	0.74 (0.70 to 0.79)	<0.001
**Race and ethnicity**					
Black or African American non-Hispanic	1654/113,256 (1.5%)	0.89 (0.84 to 0.94)	<0.001	0.81 (0.76 to 0.85)	<0.001
Hispanic or Latino any race	703/42,304 (1.7%)	1.02 (0.95 to 1.11)	0.56	1.00 (0.93 to 1.08)	0.95
Other non-Hispanic	465/35,368 (1.3%)	0.80 (0.73 to 0.88)	<0.001	0.87 (0.79 to 0.96)	0.004
Unknown	249/17,288 (1.4%)	0.89 (0.79 to 1.01)	0.08	0.95 (0.84 to 1.08)	0.45
White non-Hispanic	8227/499,134 (1.6%)	Reference		Reference	
**SARS-CoV-2 variant period**					
Omicron (B.1.1.529, BA.2, BA.2.12.1)	5074/282,580 (1.8%)	Reference		Reference	
Delta (B.1.617.2)	1793/65,206 (2.7%)	1.54 (1.46 to 1.62)	<0.001	1.51 (1.43 to 1.60)	<0.001
Omicron (BA.5, BQ.1.1, XBB.1.5)	4431/359,564 (1.2%)	0.70 (0.67 to 0.72)	<0.001	0.69 (0.66 to 0.72)	<0.001
**COVID-19 vaccination status**					
No documented COVID-19 vaccination	6794/387,606 (1.8%)	Reference		Reference	
Primary COVID-19 vaccination documented	1909/124,536 (1.5%)	0.87 (0.82 to 0.91)	<0.001	0.80 (0.76 to 0.84)	<0.001
Primary and additional COVID-19 dose(s) documented	2595/195,208 (1.3%)	0.75 (0.72 to 0.79)	<0.001	0.84 (0.80 to 0.88)	<0.001
**Heart failure**					
No history of heart failure	8914/587,082 (1.5%)	Reference		Reference	
History of heart failure	2384/120,268 (2.0%)	1.31 (1.26 to 1.37)	<0.001	1.04 (0.99 to 1.10)	0.15
**Diabetes**					
No history of diabetes	7419/493,329 (1.5%)	Reference		Reference	
History of diabetes	3879/214,021 (1.8%)	1.21 (1.16 to 1.26)	<0.001	0.99 (0.95 to 1.03)	0.62
**Hypertension**					
No history of HTN	3204/244,962 (1.3%)	Reference		Reference	
History of HTN	8094/462,388 (1.8%)	1.35 (1.29 to 1.40)	<0.001	1.14 (1.09 to 1.20)	<0.001
**CVD**					
No history of CVD	5164/378,348 (1.4%)	Reference		Reference	
History of CVD	6134/329,002 (1.9%)	1.37 (1.33 to 1.43)	<0.001	1.30 (1.24 to 1.36)	<0.001
**Obesity**					
No history of obesity	4639/363,531 (1.3%)	Reference		Reference	
History of obesity	6659/343,819 (1.9%)	1.52 (1.47 to 1.58)	<0.001	1.35 (1.30 to 1.41)	<0.001
**Tobacco usage**					
No history of tobacco usage	9345/602,792 (1.6%)	Reference		Reference	
History of tobacco usage	1953/104,558 (1.9%)	1.21 (1.15 to 1.27)	<0.001	1.12 (1.06 to 1.18)	<0.001
**Rurality**					
Urban	8064/512,014 (1.6%)	Reference		Reference	
Rural	2006/117,088 (1.7%)	1.08 (1.03 to 1.13)	0.003	1.01 (0.96 to 1.07)	0.60
Missing	1228/78,248 (1.6%)	1.01 (0.95 to 1.07)	0.75	0.97 (0.91 to 1.03)	0.30

Table 2 includes univariate and multivariable Cox proportional hazards for long coronavirus (U09.9) among patients with an acute respiratory syndrome coronavirus infection documented between October 1, 2021, and September 30, 2023, after propensity score matching. Patients were propensity score matching using logistic regression with 1:1 matching for binary long coronavirus status with exact matching on sex, race and ethnicity, coronavirus disease 2019 variant period, and data contributing site and nearest neighbor propensity score matching on age. cHR, cause-specific hazard ratio; CI, confidence interval; COVID-19, Coronavirus disease 2019; CVD, cardiovascular disease; HR, hazard ratio; HTN, hypertension; *N*, Number of Patients; *N* Events, Number of Events; sHR, subdistribution hazard ratio; SARS-CoV-2, acute respiratory syndrome coronavirus.

Patients with mild CKD had a greater incidence of Long COVID than those without CKD/ESKD (Supplemental Figures 4 and 5 and Supplemental Tables 3 and 4). Baseline mild CKD was significantly associated with Long COVID in multivariable models of the overall cohort (sHR, 1.21; 95% CI 1.15 to 1.29; Supplemental Table 5) and the PSM cohort (sHR 1.15; 95% CI, 1.05 to 1.25; Supplemental Table 6).

### Association of Long COVID with Incident CKD/ESKD

**Figure 3:**
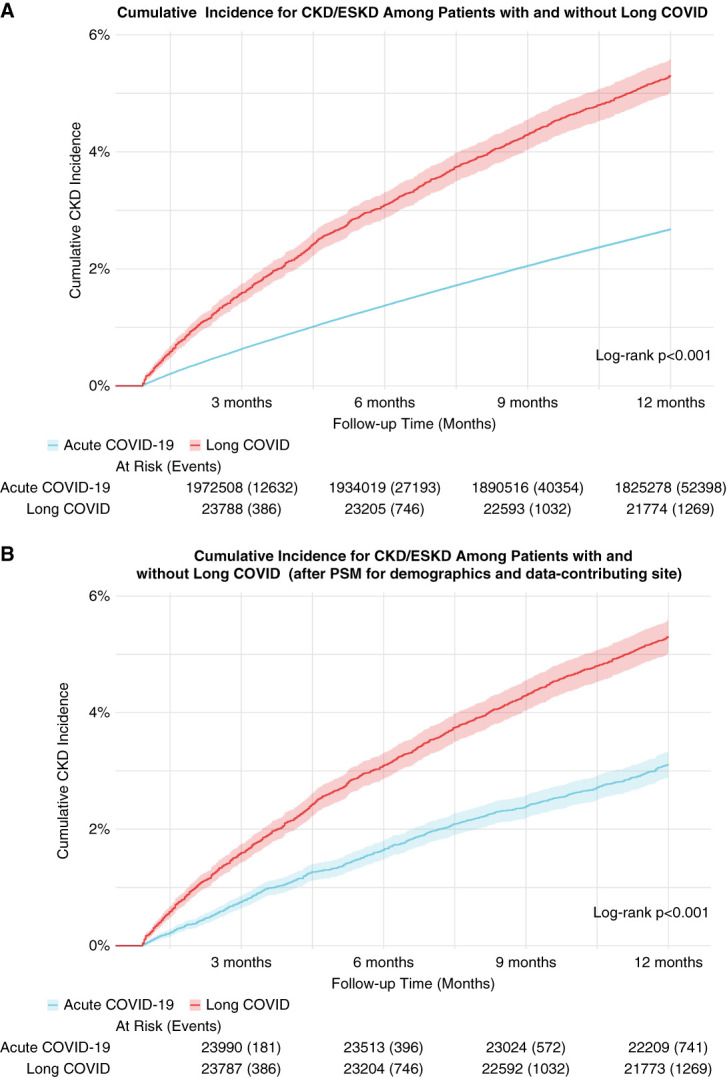
**Cumulative incidence for CKD/ESKD.** (A) Cumulative incidence for CKD/ESKD among patients with and without Long COVID. Figure 3A presents cumulative incidence for CKD/ESKD from month 1 to month 12 after initial SARS-CoV-2 infection for patients with and without Long COVID. (B) Cumulative Incidence for CKD/ESKD among patients with and without Long COVID (after PSM for demographics and data contributing site). Figure 3B presents cumulative incidence for CKD/ESKD from month 1 to month 12 after initial SARS-CoV-2 infection for patients with and without Long COVID.

Among 2,020,162 patients with acute COVID-19 with no CKD/ESKD at baseline, those who developed Long COVID (*N*=24,260, 1.2% of the cohort) were more likely to have incident CKD (5.2% versus 2.6%) and die (1.6% versus 0.9%) in the post–COVID-19 period (Figure 3A, Supplemental Figure 6 and Supplemental Table 7). In the PSM cohort (Supplemental Figure 7), those who developed Long COVID (compared with those who did not) were also more likely to have new-onset CKD/ESKD (7.9% versus 6.4%) in the post-COVID period (Figure 3B, Table 3, and Supplemental Figure 8).

**Table 3:** Descriptive statistics after propensity score matching for patients with and without Long COVID after propensity score matching

Characteristic^a^	Overall, *N*=48,518	No Long COVID, *n*=24,259	Long COVID, *n*=24,259	SMD (95% CI)^b^
Age at COVID-19	52 (40–63)	52 (40–63)	52 (40–63)	−0.01 (−0.02 to 0.01)
**Age group**				0.01 (−0.01 to 0.02)
18–29	4086 (8%)	2051 (9%)	2035 (8%)	
30–39	7431 (15%)	3719 (15%)	3712 (15%)	
40–49	9844 (20%)	4932 (20%)	4912 (20%)	
50–59	11,018 (23%)	5513 (23%)	5505 (23%)	
60–69	9433 (19%)	4712 (19%)	4721 (19%)	
70–79	5080 (10%)	2532 (10%)	2548 (11%)	
80+	1626 (3%)	800 (3%)	826 (3%)	
**Sex**				0.00 (−0.02 to 0.02)
Female	33,250 (69%)	16,625 (69%)	16,625 (69%)	
Male	15,268 (31%)	7634 (31%)	7634 (31%)	
**Race and ethnicity**				0.00 (−0.02 to 0.02)
Black or African American non-Hispanic	4494 (9%)	2247 (9%)	2247 (9%)	
Hispanic or Latino any race	4840 (10.0%)	2420 (10.0%)	2420 (10.0%)	
Other non-Hispanic	2692 (6%)	1346 (6%)	1346 (6%)	
Unknown	2134 (4%)	1067 (4%)	1067 (4%)	
White non-Hispanic	34,358 (71%)	17,179 (71%)	17,179 (71%)	
**Rurality**				0.01 (0.00 to 0.03)
Urban	35,814 (74%)	17,841 (74%)	17,973 (74%)	
Rural	7287 (15%)	3705 (15%)	3582 (15%)	
Missing	*5417 (11%)*	*2713 (11%)*	*2704 (11%)*	
**COVID-19 vaccination status**				0.05 (0.03 to 0.06)
No documented COVID-19 vaccination	31,890 (66%)	15,750 (65%)	16,140 (67%)	
Primary COVID-19 vaccination documented	8458 (17%)	4437 (18%)	4021 (17%)	
Primary and additional COVID-19 dose(s) documented	8170 (17%)	4072 (17%)	4098 (17%)	
**SARS-CoV-2 variant-dominant period**				0.00 (−0.02 to 0.02)
Delta (B.1.617.2)	9298 (19%)	4649 (19%)	4649 (19%)	
Omicron (B.1.1.529, BA.2, BA.2.12.1)	22,956 (47%)	11,478 (47%)	11,478 (47%)	
Omicron (BA.5, BQ.1.1, XBB.1.5)	16,264 (34%)	8132 (34%)	8132 (34%)	
Heart failure before COVID-19	2000 (4%)	864 (4%)	1136 (5%)	−0.06 (−0.07 to −0.04)
CVD before COVID-19	10,767 (22%)	4696 (19%)	6071 (25%)	−0.14 (−0.15 to −0.12)
Hypertension before COVID-19	16,836 (35%)	7912 (33%)	8924 (37%)	−0.09 (−0.11 to −0.07)
Obesity before COVID-19	23,153 (48%)	10,899 (45%)	12,254 (51%)	−0.11 (−0.13 to −0.09)
Tobacco usage before COVID-19	7301 (15%)	3677 (15%)	3624 (15%)	0.01 (−0.01 to 0.02)
Diabetes before COVID-19	7003 (14%)	3331 (14%)	3672 (15%)	−0.04 (−0.06 to −0.02)
**Baseline eGFR before COVID-19**	94 (82–105)	94 (82–105)	94 (82–105)	0.01 (−0.01 to 0.04)
No eGFR available in the yr before COVID-19	*23,886*	*12,831*	*11,055*	
**Adverse post–COVID-19 events**				
Incident CKD/ESKD	2010 (4%)	741 (3%)	1269 (5%)	−0.11 (−0.13 to −0.09)
Death after COVID-19 (with no long COVID diagnosis)	480 (1%)	181 (1%)	299 (1%)	−0.05 (−0.07 to −0.03)
Any death after COVID-19	585 (1%)	208 (01%)	377 (2%)	−0.06 (−0.08 to −0.05)

Table 3 presents descriptive statistics for patients with a documented acute respiratory syndrome coronavirus infection between October 1, 2021, and September 30, 2023, after propensity score matching. Patients were propensity score matching using logistic regression with 1:1 matching for binary long coronavirus status with exact matching on sex, race and ethnicity, coronavirus disease variant period, and data contributing site and nearest neighbor propensity score matching on age. This includes all patients seen at clinics with ≥250 long coronavirus cases based on study inclusion/exclusion criteria. CI, confidence interval; COVID-19, coronavirus disease 2019; CVD, cardiovascular disease; SARS-CoV-2, acute respiratory syndrome coronavirus; SMD, standardized mean differences.

a *n* (%); median (interquartile range).

b Standardized mean differences with 95% confidence interval.

In univariate Cox regression (Supplemental Table 8), Long COVID was associated with incident CKD/ESKD (HR, 2.02; 95% CI, 1.91 to 2.13). This association remained significant in multivariable models after adjusting for sex, age, race and ethnicity, vaccination status, comorbid conditions, rurality, and the competing risk of death (sHR, 1.66; 95% CI, 1.57 to 1.76). Long COVID remained significantly associated with incident CKD in multivariable models of the PSM cohort (sHR, 1.65; 95% CI, 1.51–1.81; Table 4). Other significant risk factors were older age, Black race, unvaccinated status, rural residence, and history of heart failure, DM, hypertension, CVD, and obesity.

**Table 4:** Univariate and multivariable Cox proportional hazards regression for incident CKD/ESKD after propensity score matching for demographics and data contributing site

Characteristic	*N* Events/*N* (%)	Unadjusted HR (95% CI)	*P* Value	Adjusted sHR (95% CI)	*P* Value
**COVID-19 status**					
Acute COVID-19	741/24,259 (3.1%)	Reference		Reference	
Long COVID	1269/24,259 (5.2%)	1.73 (1.58, 1.90)	<0.001	1.65 (1.51, 1.81)	<0.001
**Sex**					
Female	1226/33,250 (3.7%)	Reference		Reference	
Male	784/15,268 (5.1%)	1.40 (1.28, 1.53)	<0.001	1.11 (1.01, 1.22)	0.03
**Age group**					
60–69	595/9433 (6.3%)	Reference		Reference	
18–29	22/4086 (0.54%)	0.08 (0.05, 0.13)	<0.001	0.12 (0.08, 0.18)	<0.001
30–39	72/7431 (0.97%)	0.15 (0.12, 0.19)	<0.001	0.19 (0.15, 0.25)	<0.001
40–49	187/9844 (1.9%)	0.29 (0.25, 0.35)	<0.001	0.35 (0.29, 0.41)	<0.001
50–59	422/11,018 (3.8%)	0.60 (0.53, 0.68)	<0.001	0.64 (0.57, 0.73)	<0.001
70–79	480/5080 (9.4%)	1.52 (1.35, 1.72)	<0.001	1.49 (1.32, 1.69)	<0.001
80+	232/1626 (14%)	2.40 (2.06, 2.79)	<0.001	2.22 (1.90, 2.60)	<0.001
**Race and ethnicity**					
Black or African American non-Hispanic	247/4494 (5.5%)	1.24 (1.08, 1.41)	0.002	1.51 (1.31, 1.75)	<0.001
Hispanic or Latino any race	110/4840 (2.3%)	0.51 (0.42, 0.62)	<0.001	0.68 (0.56, 0.83)	<0.001
Other non-Hispanic	67/2692 (2.5%)	0.56 (0.44, 0.71)	<0.001	0.73 (0.57, 0.94)	0.01
Unknown	48/2134 (2.2%)	0.51 (0.38, 0.68)	<0.001	0.72 (0.54, 0.97)	0.03
White non-Hispanic	1538/34,358 (4.5%)	Reference		Reference	
**SARS-CoV-2 variant period**					
Omicron (B.1.1.529, BA.2, BA.2.12.1)	907/22,956 (4.0%)	Reference		Reference	
Delta (B.1.617.2)	404/9298 (4.3%)	1.10 (0.98, 1.24)	0.11	1.05 (0.93, 1.18)	0.44
Omicron (BA.5, BQ.1.1, XBB.1.5)	699/16,264 (4.3%)	1.12 (1.02, 1.24)	0.02	0.95 (0.86, 1.05)	0.31
**COVID-19 vaccination status**					
No documented COVID-19 vaccination	1331/31,890 (4.2%)	Reference		Reference	
Primary COVID-19 vaccination documented	318/8458 (3.8%)	0.89 (0.79, 1.01)	0.07	0.88 (0.77, 0.99)	0.04
Primary and additional COVID-19 Dose(s) documented	361/8170 (4.4%)	1.06 (0.94, 1.19)	0.37	0.84 (0.75, 0.95)	0.006
**Heart failure**					
No history of heart failure	1756/46,518 (3.8%)	Reference		Reference	
History of heart failure	254/2000 (13%)	3.54 (3.10, 4.03)	<0.001	1.41 (1.21, 1.64)	<0.001
**Diabetes**					
No history of diabetes	1437/41,515 (3.5%)	Reference		Reference	
History of diabetes	573/7003 (8.2%)	2.42 (2.20, 2.67)	<0.001	1.44 (1.29, 1.60)	<0.001
**Hypertension**					
No history of HTN	802/31,682 (2.5%)	Reference		Reference	
History of HTN	1208/16,836 (7.2%)	2.90 (2.66, 3.18)	<0.001	1.31 (1.18, 1.46)	<0.001
**CVD**					
No history of CVD	1161/37,751 (3.1%)	Reference		Reference	
History of CVD	849/10,767 (7.9%)	2.64 (2.41, 2.88)	<0.001	1.24 (1.12, 1.38)	<0.001
**Obesity**					
No history of obesity	888/25,365 (3.5%)	Reference		Reference	
History of obesity	1122/23,153 (4.8%)	1.39 (1.27, 1.52)	<0.001	1.14 (1.04, 1.26)	0.006
**Tobacco usage**					
No history of tobacco usage	1686/41,217 (4.1%)	Reference		Reference	
History of tobacco usage	324/7301 (4.4%)	1.08 (0.96, 1.22)	0.19	1.03 (0.92, 1.16)	0.60
**Rurality**					
Urban	1399/35,814 (3.9%)	Reference		Reference	
Rural	376/7287 (5.2%)	1.31 (1.17, 1.47)	<0.001	1.15 (1.02, 1.29)	0.02
Missing	235/5417 (4.3%)	1.13 (0.98, 1.29)	0.09	1.12 (0.97, 1.29)	0.11

Table 4 includes univariate and multivariable Cox Proportional Hazards for incident CKD/ESKD among patients with an acute respiratory syndrome coronavirus infection documented between October 1, 2021, and September 30, 2023, after propensity score matching. Patients are followed up for 365 days for an outcome of CKD. CI, confidence interval; COVID-19, coronavirus disease 2019; CVD, cardiovascular disease; HR, hazard ratio; *N*, Number of Patients; *N* Events, Number of Events; sHR, subdistribution hazard ratio; SARS-CoV-2, acute respiratory syndrome coronavirus.

### Association of Long COVID with eGFR Decline

In a subanalysis of 212,470 patients who had at least two eGFR measures after acute COVID and without a history of CKD/ESKD, those who developed Long COVID (compared with those who did not) were more likely to have a significant eGFR decline (7.9% versus 6.4%) in the post–COVID-19 period (Supplemental Figures 9 and 10 and Supplemental Table 9). In the PSM cohort (Supplemental Figure 11), those who developed Long COVID were also more likely to have eGFR decline (7.9% versus 6.5%) in the post-COVID period (Supplemental Figures 12 and 13 and Supplemental Table 10).

Long COVID was significantly associated with eGFR decline in univariate Cox regression (HR, 1.21; 95% CI, 1.24 to 1.37) and in multivariable models (Supplemental Table 11) after adjusting for sex, age, race and ethnicity, vaccination status, comorbid conditions, rurality, and the competing risk of death (sHR, 1.21; 95% CI, 1.10 to 1.34). Long COVID remained associated with eGFR decline in adjusted models after PSM (sHR, 1.21; 95% CI, 1.04 to 1.40; Supplemental Table 12).

## Sensitivity Analyses

Using a stricter baseline CKD definition requiring two eGFR measurements <60 ml/min per 1.73 m^2^ at least 90 days apart (Supplemental Tables 13–18), baseline CKD/ESKD remained significantly associated with Long COVID (PSM sHR, 1.12; 95% CI, 1.08 to 1.16), Long COVID remained associated with incident CKD/ESKD (PSM sHR, 1.63; 95% CI, 1.49 to 1.78), and Long COVID remained associated with eGFR decline (PSM sHR, 1.19; 95% CI, 1.03 to 1.38). These results were consistent with the primary PSM findings, with minimal change in effect sizes.

After adjusting for acute COVID-19 severity (Supplemental Tables 19–21), baseline CKD/ESKD remained associated with Long COVID (PSM sHR, 1.08; 95% CI, 1.03 to 1.12), Long COVID remained associated with incident CKD/ESKD (PSM sHR, 1.51; 95% CI, 1.37 to 1.65), and no association was observed between Long COVID and eGFR decline (PSM sHR, 1.13; 95% CI, 0.97 to 1.31). These estimates were similar to the primary models, indicating that COVID-19 severity at presentation did not materially alter the observed associations. COVID-19 severity (moderate or severe versus mild) was associated with Long COVID, incident CKD, and eGFR decline.

When stratifying by age group (younger than 65 versus 65 years and older; Supplemental Tables 22–27), baseline CKD/ESKD remained significantly associated with Long COVID and Long COVID remained significantly associated with incident CKD/ESKD and post-COVID eGFR decline across both age strata. Subdistribution hazard ratios remained similar to the main PSM estimates in each age group, supporting the robustness of the associations across age cohorts.

## Discussion

In this study, we report that among patients with acute COVID-19, those with baseline CKD or ESKD have a higher incidence of Long COVID than those without. Even mild CKD (Stage 3a: eGFR, 45–59 ml/min per 1.73 m^2^) was associated with risk of Long COVID. Among those without CKD at baseline, those who developed Long COVID had a greater incidence of CKD when compared with those without Long COVID. Long COVID was also associated with eGFR decline. The findings remained robust even after adjusting for traditional risk factors and propensity matching for age, sex, race and ethnicity, and data contributing site. To the best of our knowledge, this is the largest study to report the association between Long COVID and CKD. Our findings highlight baseline CKD as a risk factor for Long COVID and support the inclusion of incident CKD as a manifestation of Long COVID.

The robustness of our findings was further supported by sensitivity analyses, which demonstrated consistent associations across alternative CKD definitions, adjustment for acute COVID-19 severity, and stratification by age group. These analyses reinforce that the observed relationships between CKD, Long COVID, and subsequent kidney outcomes were not materially influenced by case definition, disease severity, or age.

In a meta-analysis, Tsampasian *et al.* observed multiple factors associated with increased risk of Long COVID.^6^ However, in a pooled analysis of eight studies that included 255,791 patients, they observed that CKD was not a significant risk factor. Among these eight studies, in a Veteran Affairs study of 198,601 SARS-CoV-2–positive persons,^7^ CKD was associated with Long COVID. By contrast, in a smaller study of 238 patients, CKD was reported as a risk factor only in those with reduced lung oxygenation capacity.^14^ The other studies that did not find CKD as a risk factor for Long COVID had small sample sizes and were mostly limited to single centers or health care systems.^9,15–19^ Our primary cohort had a sample size of over 2.3 million patients from multiple health care systems across the United States, with over 350,000 patients with a history of CKD/ESKD and over 30,000 cases of Long COVID. This makes ours the largest study to date investigating CKD as a risk factor for Long COVID.

After matching for key demographics and adjusting for multiple covariates and the competing risk of death, we observed that baseline CKD/ESKD was associated with an 13% and CKD Stage 3a with 15% greater risk of Long COVID in propensity matched multivariable models. Patients with CKD, especially those with ESKD on maintenance dialysis, have a greater risk of SARS-CoV-2 and getting hospitalized with COVID-19 compared with those without CKD/ESKD.^55,56^ It is possible that in our cohort, those with CKD had a greater severity of COVID-19 infection. However, the risk was equally significant with mild baseline CKD. CKD has been associated with immune system dysfunction that increases the risk and severity of infection.^57,58^ Among patients hospitalized with acute COVID-19, those with CKD also have a higher mortality compared with those without CKD.^55,59,60^ In our study, the risk of Long COVID with CKD persisted even after accounting for the competing risk of death.

Consistent with previous studies, we found that female sex and history of HTN,^2^ CVD,^7^ and obesity^2,6^ were associated with higher risk of Long COVID. We also observed an association with the Delta (B.1.617.2) variant, urban residence, and tobacco use. Patients of Black race and those with COVID-19 vaccinations had a lower risk of Long COVID. In fully adjusted models, diabetes^10^ and heart failure were not associated with increased risk of Long COVID.

In multivariable models, we also observed that among patients with acute COVID-19, those who developed Long COVID were 1.65 times more likely to have incident CKD and 1.25 times more likely to have significant eGFR decline than those without Long COVID. To the best of our knowledge, this is the first study to report this association of Long COVID with incident CKD. In our study, we used the diagnosis code (U09.9) to define Long COVID,^40^ which is commonly used in Long COVID clinics and is based on an algorithm that primarily involves patients' symptoms and signs. Our findings support the current evidence that kidney disease should be considered a potential manifestation of Long COVID, as recently proposed in the National Academies Science Engineering Medicine 2024 definition of Long COVID.^61^ While the pathophysiologic contributions of Long COVID on incident CKD and eGFR decline are plausible, our findings require further exploration. One potential explanation is that viral persistence in the kidney or systemic inflammation plays a role in the pathogenesis of incident CKD. SARS-CoV-2 is known to infect the kidneys and can result in acute and chronic kidney injury.^62^ In a mouse model of Long COVID, increased levels of inflammatory cytokines in the kidney and renal scarring have been reported.^63^

This study had several notable limitations. First, the reliance on a diagnosis code based on subjective symptoms for making a clinical diagnosis of Long COVID. Second, the exclusion of patients who had COVID-19 before October 1, 2021, due to a lack of diagnosis codes for Long COVID before this date. Third, we did not evaluate the effect of treatment of COVID-19 and SARS-Cov2 reinfections due to collinearity concerns with the primary outcomes in the study. Fourth, our definition of incident CKD relied only on eGFR or diagnosis codes and did not specifically include urinary markers (*e.g*., albuminuria). Proteinuria is associated with COVID-19 and its association with Long COVID required further investigation. Fifth, CKD was defined by only one eGFR measure in the primary analyses. However, we conducted a sensitivity analysis where CKD was defined by two eGFR values ≥90 days apart and noted similar findings. Sixth, eGFR was estimated using the serum creatinine-based equation, and measures of facility and muscle mass were not available in the dataset. However, to partially address age-related risk differences, we conducted a sensitivity analysis stratified by age group (younger than 65 versus 65 years and older) and noted similar findings. Finally, we acknowledge the potential risk of selection bias due to missing age and other key demographic variables.

In conclusion, we report that baseline CKD/ESKD (even at an early stage), incident CKD/ESKD, and eGFR decline are associated with Long COVID. Our findings have important clinical implications. We suggest that those with CKD or ESKD who get acute COVID-19 require closer monitoring for the development of Long COVID. Our study also highlights that in those without kidney disease who get acute COVID-19, close monitoring of kidney function in those with persistent symptoms is important. Further research is needed to explore the association between Long COVID and kidney disease.

## Supplementary Material

## Data Availability

Original data generated for the study will be made available upon reasonable request to the corresponding author. Aggregated Data. The N3C Enclave is available for public research use. More than 4000 researchers currently have access to data in N3C, representing more than 300 US research institutions. Institutions must have a signed Data Use Agreement executed with the US National Center for Advancing Translational Sciences to access data. Investigators must complete mandatory training and submit a Data Use Request to N3C. To request N3C data access, follow instructions at https://covid.cd2h.org/onboarding. This project uses data from N3C release 170, which can be replicated within the N3C Enclave by qualified N3C users. All concepts and definitions are provided in the Supplemental Methods. All code used for analyses can be made available upon request. Decline color. No.

## Supplemental Material

Supplemental Methods Table. Defined list of concepts for visit type, race, ethnicity, rurality, Long COVID, CKD, DM, hypertension, CVD, heart failure, ESKD, and AKI.

Supplemental Table 1. Descriptive statistics of patients with and without baseline CKD/ESKD.

Supplemental Table 2. Univariate and multivariable Cox proportional hazards regression for Long COVID.

Supplemental Table 3. Descriptive statistics of patients with and without baseline mild CKD (Stage 3a).

Supplemental Table 4. Descriptive statistics of patients with and without baseline mild CKD (Stage 3a) after PSM.

Supplemental Table 5. Univariate and multivariable Cox proportional hazards regression for Long COVID (mild CKD – Stage 3a).

Supplemental Table 6. Univariate and multivariable Cox proportional hazards regression for Long COVID After PSM (mild CKD – Stage 3a).

Supplemental Table 7. Descriptive statistics of patients with and without baseline Long COVID.

Supplemental Table 8. Univariate and multivariable Cox proportional hazards regression for incident CKD/ESKD.

Supplemental Table 9. Descriptive statistics for patients with and without Long COVID (among patients with at least two eGFR measurements 30–365 days post–COVID-19).

Supplemental Table 10. Descriptive statistics for patients with and without Long COVID after PSM (among patients with at least two eGFR measurements).

Supplemental Table 11. Univariate and multivariable Cox proportional hazards regression for eGFR decline (among patients with at least two eGFR measurements).

Supplemental Table 12. Univariate and multivariable Cox proportional hazards regression for eGFR decline after PSM (among patients with at least two eGFR measurements).

Sensitivity Analysis 1 (alternative CKD definition by the two GFR criteria):

Supplemental Table 13. Descriptive statistics for patients with and without CKD/ESKD after PSM (baseline CKD based on the two eGFR criteria—sensitivity analysis 1).

Supplemental Table 14. Univariate and multivariable Cox proportional hazards regression for Long COVID after PSM (sensitivity analysis 1).

Supplemental Table 15. Descriptive statistics among patients with and without Long COVID after PSM (incident CKD based on the two eGFR criteria—sensitivity analysis 1).

Supplemental Table 16. Univariate and multivariable Cox proportional hazards regression for incident CKD/ESKD after PSM (sensitivity analysis 1).

Supplemental Table 17. Descriptive statistics of patients with and without Long COVID after PSM (among patients with at least two eGFR Measurements—sensitivity analysis 1).

Supplemental Table 18. Univariate and multivariable Cox proportional hazards regression for eGFR decline after PSM (sensitivity analysis 1).

Sensitivity analysis 2 (adjustment for COVID-19 severity):

Supplemental Table 19. Univariate and multivariable Cox proportional hazards regression for Long COVID including adjustment for acute COVID-19 severity after PSM (sensitivity analysis 2).

Supplemental Table 20. Univariate and multivariable Cox proportional hazards regression for incident CKD/ESKD including adjustment for acute COVID-19 severity after PSM (sensitivity analysis 2).

Supplemental Table 21. Univariate and multivariable Cox proportional hazards regression for eGFR decline including adjustment for acute COVID-19 severity after PSM (sensitivity analysis 2).

Sensitivity analysis 3 (age stratification):

Supplemental Table 22. Univariate and multivariable Cox proportional hazards regression for Long COVID among patients younger than 65 years after PSM (sensitivity analysis 3).

Supplemental Table 23. Univariate and multivariable Cox proportional hazards regression for Long COVID among patients 65 and older after PSM (sensitivity analysis 3).

Supplemental Table 24. Univariate and multivariable Cox proportional hazards regression for incident CKD/ESKD among patients younger than 65 years after PSM (sensitivity analysis 3).

Supplemental Table 25. Univariate and multivariable Cox proportional hazards regression for incident CKD/ESKD among patients 65 years and older after PSM (sensitivity analysis 3).

Supplemental Table 26. Univariate and multivariable Cox proportional hazards regression for eGFR decline among patients younger than 65 years after PSM (sensitivity analysis 3).

Supplemental Table 27. Univariate and multivariable Cox proportional hazards regression for eGFR decline among patients 65 years and older after PSM (sensitivity analysis 3).

Supplemental Methods Figure

Supplemental Figure 1. Competing risk for Long COVID/death.

Supplemental Figure 2. Covariate balance plot after matching for Long COVID as an outcome.

Supplemental Figure 3. Competing risk for Long COVID/death after PSM.

Supplemental Figure 4. Cumulative incidence for Long COVID among patients with and without a history of CKD stage 3a.

Supplemental Figure 5. Cumulative incidence for Long COVID among patients with and without a history of CKD stage 3a after PSM.

Supplemental Figure 6. Competing risk for incident CKD/ESKD and death.

Supplemental Figure 7. Covariate balance plot after matching for incident CKD as an outcome.

Supplemental Figure 8. Competing risk for incident CKD/ESKD and death after PSM.

Supplemental Figure 9. Cumulative incidence of eGFR decline.

Supplemental Figure 10. Competing risk for eGFR decline and death.

Supplemental Figure 11. Covariate balance plot after matching for eGFR decline as an outcome.

Supplemental Figure 12. Cumulative incidence of eGFR decline after PSM.

Supplemental Figure 13. Competing risk for eGFR decline and death after PSM.
