# Supplementary material for: The Association of Long COVID and CKD: Findings from the National Clinical Cohort Collaborative
Source: Clin J Am Soc Nephrol. 2025 Aug 5;20(10):1323–40. doi: 10.2215/CJN.0000000773 (PMC12537286; doi:10.2215/CJN.0000000773)
Supplement: Supplementary file 1 [file cjasn-20-1323-s001.pdf]

ASN American Society of Nephrology
JASN Journal of the American Society of Nephrology
KIDNEY360 Accessing Our World From Every Angle
CJASN Clinical Journal of the American Society of Nephrology

ASN Journal Disclosure Form

As per ASN journal policy, I have disclosed any financial relationships or commitments I have held in the past 36 months as included below. I have listed my Current Employer below to indicate there is a relationship requiring disclosure. If no relationship exists, my Current Employer is not listed.

F. Alakwaa has nothing to disclose.

I understand that the information above will be published within the journal article, if accepted, and that failure to comply and/or to accurately and completely report the potential financial conflicts of interest could lead to the following: 1) Prior to publication, article rejection, or 2) Post-publication, sanctions ranging from, but not limited to, issuing a correction, reporting the inaccurate information to the authors' institution, banning authors from submitting work to ASN journals for varying lengths of time, and/or retraction of the published work.

Name: Fadhl Alakwaa
Manuscript ID: CJASN-2025-000130R1
Manuscript Title: The association of Long COVID and CKD: findings from the National COVID Cohort Collaborative (N3C)
Date of Completion: May 2, 2025
Disclosure Updated Date: March 21, 2025

ASN American Society of Nephrology | JASN Journal of the American Society of Nephrology | KIDNEY360 Accessing Our World From Every Angle | CJASN Clinical Journal of the American Society of Nephrology

ASN Journal Disclosure Form

As per ASN journal policy, I have disclosed any financial relationships or commitments I have held in the past 36 months as included below. I have listed my Current Employer below to indicate there is a relationship requiring disclosure. If no relationship exists, my Current Employer is not listed.

A. Anzalone reports the following:
Employer: University of Nebraska Medical Center

I understand that the information above will be published within the journal article, if accepted, and that failure to comply and/or to accurately and completely report the potential financial conflicts of interest could lead to the following: 1) Prior to publication, article rejection, or 2) Post-publication, sanctions ranging from, but not limited to, issuing a correction, reporting the inaccurate information to the authors' institution, banning authors from submitting work to ASN journals for varying lengths of time, and/or retraction of the published work.

Name: Alfred Jerrod Anzalone
Manuscript ID: CJASN-2025-000130R2
Manuscript Title: The association of Long COVID and CKD: findings from the National COVID Cohort Collaborative (N3C)
Date of Completion: June 20, 2025
Disclosure Updated Date: June 20, 2025

ASN American Society of Nephrology · JASN Journal of the American Society of Nephrology · KIDNEY360 Accessing Our World From Every Angle · CJASN Clinical Journal of the American Society of Nephrology

ASN Journal Disclosure Form

As per ASN journal policy, I have disclosed any financial relationships or commitments I have held in the past 36 months as included below. I have listed my Current Employer below to indicate there is a relationship requiring disclosure. If no relationship exists, my Current Employer is not listed.

J. Byrd reports the following:
Employer: University of Michigan; Consultancy: Advisory boards for Blue Earth Diagnostics and Idorsia.; Advisory or Leadership Role: I am an editorial board member of the Journal of Clinical and Translational Endocrinology (Elsevier) and I am Associate Editor of the Journal of Human Hypertension (Springer-Nature). I am Associate Editor of Hypertension Research (Springer-Nature). Advisory boards for Blue Earth Diagnostics and Idorsia.; and Other Interests or Relationships: I am funded by the NIH and PCORI.

I understand that the information above will be published within the journal article, if accepted, and that failure to comply and/or to accurately and completely report the potential financial conflicts of interest could lead to the following: 1) Prior to publication, article rejection, or 2) Post-publication, sanctions ranging from, but not limited to, issuing a correction, reporting the inaccurate information to the authors' institution, banning authors from submitting work to ASN journals for varying lengths of time, and/or retraction of the published work.

Name: J. Brian Byrd
Manuscript ID: CJASN-2025-000130
Manuscript Title: The association of Long COVID and CKD: findings from the National COVID Cohort Collaborative (N3C)
Date of Completion: March 21, 2025
Disclosure Updated Date: March 21, 2025

ASN American Society of Nephrology · JASN Journal of the American Society of Nephrology · KIDNEY360 Accessing Our World From Every Angle · CJASN Clinical Journal of the American Society of Nephrology

ASN Journal Disclosure Form

As per ASN journal policy, I have disclosed any financial relationships or commitments I have held in the past 36 months as included below. I have listed my Current Employer below to indicate there is a relationship requiring disclosure. If no relationship exists, my Current Employer is not listed.

F. Koraishy has nothing to disclose.

I understand that the information above will be published within the journal article, if accepted, and that failure to comply and/or to accurately and completely report the potential financial conflicts of interest could lead to the following: 1) Prior to publication, article rejection, or 2) Post-publication, sanctions ranging from, but not limited to, issuing a correction, reporting the inaccurate information to the authors' institution, banning authors from submitting work to ASN journals for varying lengths of time, and/or retraction of the published work.

Name: Farrukh M. Koraishy
Manuscript ID: CJASN-2025-000130R1
Manuscript Title: The association of Long COVID and CKD: findings from the National COVID Cohort Collaborative (N3C)
Date of Completion: May 2, 2025
Disclosure Updated Date: May 2, 2025

ASN American Society of Nephrology
JASN Journal of the American Society of Nephrology
KIDNEY360 Accessing Our World From Every Angle
CJASN Clinical Journal of the American Society of Nephrology

ASN Journal Disclosure Form

As per ASN journal policy, I have disclosed any financial relationships or commitments I have held in the past 36 months as included below. I have listed my Current Employer below to indicate there is a relationship requiring disclosure. If no relationship exists, my Current Employer is not listed.

S. Krichevsky reports the following:
Employer: Stony Brook University

I understand that the information above will be published within the journal article, if accepted, and that failure to comply and/or to accurately and completely report the potential financial conflicts of interest could lead to the following: 1) Prior to publication, article rejection, or 2) Post-publication, sanctions ranging from, but not limited to, issuing a correction, reporting the inaccurate information to the authors' institution, banning authors from submitting work to ASN journals for varying lengths of time, and/or retraction of the published work.

Name: Spencer Krichevsky
Manuscript ID: CJASN-2025-000130
Manuscript Title: The association of Long COVID and CKD: findings from the National COVID Cohort Collaborative (N3C)
Date of Completion: March 21, 2025
Disclosure Updated Date: March 21, 2025

ASN American Society of Nephrology
JASN Journal of the American Society of Nephrology
KIDNEY360 Accessing Our World From Every Angle
CJASN Clinical Journal of the American Society of Nephrology

ASN Journal Disclosure Form

As per ASN journal policy, I have disclosed any financial relationships or commitments I have held in the past 36 months as included below. I have listed my Current Employer below to indicate there is a relationship requiring disclosure. If no relationship exists, my Current Employer is not listed.

F. Liu reports the following:
Employer: University of Massachusetts Medical School; and Ownership Interest: Apple, Amazon, Alibaba, Palantir technologies, robinhood, costco, tesla, Ely lily, google, microsoft, SoFi, Paypal, UNM, VNOM, VLO, SPOT, SNAP, PSN, NVDA, MPLX, EXEL, APP.

I understand that the information above will be published within the journal article, if accepted, and that failure to comply and/or to accurately and completely report the potential financial conflicts of interest could lead to the following: 1) Prior to publication, article rejection, or 2) Post-publication, sanctions ranging from, but not limited to, issuing a correction, reporting the inaccurate information to the authors' institution, banning authors from submitting work to ASN journals for varying lengths of time, and/or retraction of the published work.

Name: Feifan Liu
Manuscript ID: CJASN-2025-000130R1
Manuscript Title: The association of Long COVID and CKD: findings from the National COVID Cohort Collaborative (N3C)
Date of Completion: May 2, 2025
Disclosure Updated Date: May 2, 2025

ASN American Society of Nephrology
JASN Journal of the American Society of Nephrology
KIDNEY360 Accessing Our World From Every Angle
CJASN Clinical Journal of the American Society of Nephrology

ASN Journal Disclosure Form

As per ASN journal policy, I have disclosed any financial relationships or commitments I have held in the past 36 months as included below. I have listed my Current Employer below to indicate there is a relationship requiring disclosure. If no relationship exists, my Current Employer is not listed.

S. Mallipattu reports the following:
Employer: Stony Brook Medicine; Consultancy: Wildwood Therapeutics, Inc.; L.E.K. Consulting; Dedham Group; Graticule;; Research Funding: Dialysis Clinic Inc.; Patents or Royalties: Krüppel-like factor 15 (KLF15) Small Molecule Agonists in Kidney Disease. US 63/018.247. 2023.; and Advisory or Leadership Role: Clinically Integrated Network, Board Member (Accountable Care Organization, LLC Stony Brook Medicine);.

I understand that the information above will be published within the journal article, if accepted, and that failure to comply and/or to accurately and completely report the potential financial conflicts of interest could lead to the following: 1) Prior to publication, article rejection, or 2) Post-publication, sanctions ranging from, but not limited to, issuing a correction, reporting the inaccurate information to the authors' institution, banning authors from submitting work to ASN journals for varying lengths of time, and/or retraction of the published work.

Name: Sandeep K. Mallipattu
Manuscript ID: CJASN-2025-000130
Manuscript Title: The association of Long COVID and CKD: findings from the National COVID Cohort Collaborative (N3C)
Date of Completion: March 22, 2025
Disclosure Updated Date: March 18, 2025

ASN American Society of Nephrology
JASN Journal of the American Society of Nephrology
KIDNEY360 Accessing Our World From Every Angle
CJASN Clinical Journal of the American Society of Nephrology

ASN Journal Disclosure Form

As per ASN journal policy, I have disclosed any financial relationships or commitments I have held in the past 36 months as included below. I have listed my Current Employer below to indicate there is a relationship requiring disclosure. If no relationship exists, my Current Employer is not listed.

R. Moffitt reports the following:
Employer: Emory University; Ownership Interest: Aflac, Global Payments Inc; and Patents or Royalties: GeneCentric, Tempus AI.

I understand that the information above will be published within the journal article, if accepted, and that failure to comply and/or to accurately and completely report the potential financial conflicts of interest could lead to the following: 1) Prior to publication, article rejection, or 2) Post-publication, sanctions ranging from, but not limited to, issuing a correction, reporting the inaccurate information to the authors' institution, banning authors from submitting work to ASN journals for varying lengths of time, and/or retraction of the published work.

Name: Richard A. Moffitt
Manuscript ID: CJASN-2025-000130R2
Manuscript Title: he association of Long COVID and CKD: findings from the National COVID Cohort Collaborative (N3C).
Date of Completion: July 9, 2025
Disclosure Updated Date: July 9, 2025

ASN
American Society of Nephrology
JASN
Journal of the American Society of Nephrology
KIDNEY360
Accessing Our World From Every Angle
CJASN
Clinical Journal of the American Society of Nephrology

ASN Journal Disclosure Form

As per ASN journal policy, I have disclosed any financial relationships or commitments I have held in the past 36 months as included below. I have listed my Current Employer below to indicate there is a relationship requiring disclosure. If no relationship exists, my Current Employer is not listed.

C. Parikh reports the following:
Employer: Johns Hopkins University School of Medicine; Ownership Interest: Renalytix; Research Funding: National Institute of Diabetes and Digestive and Kidney Diseases (NIDDK); National Heart, Lung and Blood Institute (NHLBI); AstraZeneca; Alexion; and Advisory or Leadership Role: AstraZeneca; Alexion; Panoramic Science.

I understand that the information above will be published within the journal article, if accepted, and that failure to comply and/or to accurately and completely report the potential financial conflicts of interest could lead to the following: 1) Prior to publication, article rejection, or 2) Post-publication, sanctions ranging from, but not limited to, issuing a correction, reporting the inaccurate information to the authors' institution, banning authors from submitting work to ASN journals for varying lengths of time, and/or retraction of the published work.

Name: Chirag R. Parikh
Manuscript ID: CJASN-2025-000130R2
Manuscript Title: "The association of Long COVID and CKD: findings from the National COVID Cohort Collaborative (N3C)"
Date of Completion: July 11, 2025
Disclosure Updated Date: April 23, 2025

ASN
American Society of Nephrology
JASN
Journal of the American Society of Nephrology
KIDNEY360
Accessing Our World From Every Angle
CJASN
Clinical Journal of the American Society of Nephrology

ASN Journal Disclosure Form

As per ASN journal policy, I have disclosed any financial relationships or commitments I have held in the past 36 months as included below. I have listed my Current Employer below to indicate there is a relationship requiring disclosure. If no relationship exists, my Current Employer is not listed.

A. Sakhuja reports the following:
Employer: Icahn School of Medicine at Mount Sinai; Ownership Interest: multiple technology, utility etc companies. No stock in health related companies.; Honoraria: SCCM, ASPEN; Patents or Royalties: Provision Patent Application No. 63/698,447; Advisory or Leadership Role: Carolinas/Virginia's chapter of SCCM - unpaid; and Other Interests or Relationships: Current Funding from NIH/NIDDK K08DK131286; PI: Ankit Sakhuja.

I understand that the information above will be published within the journal article, if accepted, and that failure to comply and/or to accurately and completely report the potential financial conflicts of interest could lead to the following: 1) Prior to publication, article rejection, or 2) Post-publication, sanctions ranging from, but not limited to, issuing a correction, reporting the inaccurate information to the authors' institution, banning authors from submitting work to ASN journals for varying lengths of time, and/or retraction of the published work.

Name: Ankit Sakhuja
Manuscript ID: CJASN-2025-000130R1
Manuscript Title: The association of Long COVID and CKD: findings from the National COVID Cohort Collaborative (N3C)
Date of Completion: May 2, 2025
Disclosure Updated Date: May 2, 2025

ASN
American Society of Nephrology
JASN
Journal of the American Society of Nephrology
KIDNEY360
Accessing Our World From Every Angle
CJASN
Clinical Journal of the American Society of Nephrology

ASN Journal Disclosure Form

As per ASN journal policy, I have disclosed any financial relationships or commitments I have held in the past 36 months as included below. I have listed my Current Employer below to indicate there is a relationship requiring disclosure. If no relationship exists, my Current Employer is not listed.

J. Saltz reports the following:
Employer: Stony Brook; and Advisory or Leadership Role: Memorial Sloan Kettering; Mark Foundation Both are academic, neither are companies.

I understand that the information above will be published within the journal article, if accepted, and that failure to comply and/or to accurately and completely report the potential financial conflicts of interest could lead to the following: 1) Prior to publication, article rejection, or 2) Post-publication, sanctions ranging from, but not limited to, issuing a correction, reporting the inaccurate information to the authors' institution, banning authors from submitting work to ASN journals for varying lengths of time, and/or retraction of the published work.

Name: Joel Haskin Saltz
Manuscript ID: CJASN-2025-000130R2
Manuscript Title: The association of Long COVID and CKD: findings from the National COVID Cohort Collaborative (N3C)
Date of Completion: July 24, 2025
Disclosure Updated Date: July 24, 2025

ASN
American Society of Nephrology
JASN
Journal of the American Society of Nephrology
KIDNEY360
Accessing Our World From Every Angle
CJASN
Clinical Journal of the American Society of Nephrology

ASN Journal Disclosure Form

As per ASN journal policy, I have disclosed any financial relationships or commitments I have held in the past 36 months as included below. I have listed my Current Employer below to indicate there is a relationship requiring disclosure. If no relationship exists, my Current Employer is not listed.

R. Saran reports the following:
Employer: University of Michigan; Ann Arbor VA Health System; Advisory or Leadership Role: Advisory Group Member, National Kidney Foundation of Michigan, Ann Arbor, MI; International Advisory Group, World NCD Federation, Chandigarh, India; and Other Interests or Relationships: Health Synergy Alliance.

I understand that the information above will be published within the journal article, if accepted, and that failure to comply and/or to accurately and completely report the potential financial conflicts of interest could lead to the following: 1) Prior to publication, article rejection, or 2) Post-publication, sanctions ranging from, but not limited to, issuing a correction, reporting the inaccurate information to the authors' institution, banning authors from submitting work to ASN journals for varying lengths of time, and/or retraction of the published work.

Name: Rajiv Saran
Manuscript ID: CJASN-2025-000130R2
Manuscript Title: The association of Long COVID and CKD: findings from the National COVID Cohort Collaborative (N3C),
Date of Completion: July 18, 2025
Disclosure Updated Date: July 18, 2025

ASN American Society of Nephrology | JASN Journal of the American Society of Nephrology | KIDNEY360 Accessing Our World From Every Angle | CJASN Clinical Journal of the American Society of Nephrology

ASN Journal Disclosure Form

As per ASN journal policy, I have disclosed any financial relationships or commitments I have held in the past 36 months as included below. I have listed my Current Employer below to indicate there is a relationship requiring disclosure. If no relationship exists, my Current Employer is not listed.

S. Setoguchi reports the following:
Employer: Rutgers Robert Wood Johnson Medical School; Consultancy: Pfizer Inc.; Pfizer Japan; Merck Inc.; Medtronic; BMS; Regeneron Inc;; Research Funding: Pfizer Inc; Pfizer Japan; Daiichi Sankyo;; and Advisory or Leadership Role: Editorial Board for Pharmacoepidemiology and Drug Safety.

I understand that the information above will be published within the journal article, if accepted, and that failure to comply and/or to accurately and completely report the potential financial conflicts of interest could lead to the following: 1) Prior to publication, article rejection, or 2) Post-publication, sanctions ranging from, but not limited to, issuing a correction, reporting the inaccurate information to the authors' institution, banning authors from submitting work to ASN journals for varying lengths of time, and/or retraction of the published work.

Name: Soko Setoguchi
Manuscript ID: CJASN-2025-000130R1
Manuscript Title: The association of Long COVID and CKD: findings from the National COVID Cohort Collaborative (N3C)
Date of Completion: May 2, 2025
Disclosure Updated Date: May 2, 2025

ASN American Society of Nephrology · JASN Journal of the American Society of Nephrology · KIDNEY360 Accessing Our World From Every Angle · CJASN Clinical Journal of the American Society of Nephrology

ASN Journal Disclosure Form

As per ASN journal policy, I have disclosed any financial relationships or commitments I have held in the past 36 months as included below. I have listed my Current Employer below to indicate there is a relationship requiring disclosure. If no relationship exists, my Current Employer is not listed.

K. Wilkins reports the following:
Employer: National Institutes of Health, National Institute of Diabetes & Digestive & Kidney Diseases; Advisory or Leadership Role: International Journal of Obesity - editorial board (unpaid); in process to join the Scientific Knowledge Accelerator Foundation Board, subject to Federal Ethics approval (unpaid); and Other Interests or Relationships: I have previously made commitments to involve members of kidney patient/advocacy organizations in kidney research conferences or technical expert panels: American Association of Kidney Patients (via Board members including Jenny Kitsen, Richard Knight, Paul Conway, and Ed Hickey); Renal Support Network (via President and Founder Lori Hartwell); only correspondence with similar organizations or advocates within last 24 months is Voice of the Patient (via Kevin Fowler).

I understand that the information above will be published within the journal article, if accepted, and that failure to comply and/or to accurately and completely report the potential financial conflicts of interest could lead to the following: 1) Prior to publication, article rejection, or 2) Post-publication, sanctions ranging from, but not limited to, issuing a correction, reporting the inaccurate information to the authors' institution, banning authors from submitting work to ASN journals for varying lengths of time, and/or retraction of the published work.

Name: Kenneth J. Wilkins
Manuscript ID: CJASN-2025-000130R2
Manuscript Title: The association of Long COVID and CKD: findings from the National COVID Cohort Collaborative (N3C)
Date of Completion: July 18, 2025
Disclosure Updated Date: May 29, 2025

ASN American Society of Nephrology | JASN Journal of the American Society of Nephrology | KIDNEY360 Accessing Our World From Every Angle | CJASN Clinical Journal of the American Society of Nephrology

ASN Journal Disclosure Form

As per ASN journal policy, I have disclosed any financial relationships or commitments I have held in the past 36 months as included below. I have listed my Current Employer below to indicate there is a relationship requiring disclosure. If no relationship exists, my Current Employer is not listed.

Y. Yoo reports the following:
Employer: Emory University; and Research Funding: NIH.

I understand that the information above will be published within the journal article, if accepted, and that failure to comply and/or to accurately and completely report the potential financial conflicts of interest could lead to the following: 1) Prior to publication, article rejection, or 2) Post-publication, sanctions ranging from, but not limited to, issuing a correction, reporting the inaccurate information to the authors' institution, banning authors from submitting work to ASN journals for varying lengths of time, and/or retraction of the published work.

Name: Yun Jae Yoo
Manuscript ID: CJASN-2025-000130R2
Manuscript Title: The association of Long COVID and CKD: findings from the National COVID Cohort Collaborative (N3C)
Date of Completion: June 21, 2025
Disclosure Updated Date: March 21, 2025

ASN
American Society of Nephrology
JASN
Journal of the American Society of Nephrology
KIDNEY360
Accessing Our World From Every Angle
CJASN
Clinical Journal of the American Society of Nephrology

ASN Journal Disclosure Form

As per ASN journal policy, I have disclosed any financial relationships or commitments I have held in the past 36 months as included below. I have listed my Current Employer below to indicate there is a relationship requiring disclosure. If no relationship exists, my Current Employer is not listed.

R. Zhu reports the following:
Employer: Johns Hopkins Medicine

I understand that the information above will be published within the journal article, if accepted, and that failure to comply and/or to accurately and completely report the potential financial conflicts of interest could lead to the following: 1) Prior to publication, article rejection, or 2) Post-publication, sanctions ranging from, but not limited to, issuing a correction, reporting the inaccurate information to the authors' institution, banning authors from submitting work to ASN journals for varying lengths of time, and/or retraction of the published work.

Name: Richard L. Zhu
Manuscript ID: CJASN-2025-000130R1
Manuscript Title: The association of Long COVID and CKD: findings from the National COVID Cohort Collaborative (N3C)
Date of Completion: May 5, 2025
Disclosure Updated Date: May 5, 2025

ASN
American Society of Nephrology
JASN
Journal of the American Society of Nephrology
KIDNEY360
Accessing Our World From Every Angle
CJASN
Clinical Journal of the American Society of Nephrology

ASN Journal Disclosure Form

As per ASN journal policy, I have disclosed any financial relationships or commitments I have held in the past 36 months as included below. I have listed my Current Employer below to indicate there is a relationship requiring disclosure. If no relationship exists, my Current Employer is not listed.

D. Ellison reports the following:
Employer: Oregon Health & Science University; Patents or Royalties: Author, UpToDate; and Advisory or Leadership Role: Deputy Editor, Journal of the American Society of Nephrology.

I understand that the information above will be published within the journal article, if accepted, and that failure to comply and/or to accurately and completely report the potential financial conflicts of interest could lead to the following: 1) Prior to publication, article rejection, or 2) Post-publication, sanctions ranging from, but not limited to, issuing a correction, reporting the inaccurate information to the authors' institution, banning authors from submitting work to ASN journals for varying lengths of time, and/or retraction of the published work.

Name: David H. Ellison
Manuscript ID: 2025-000130R1
Manuscript Title: he association of Long COVID and CKD: findings from the National COVID Cohort Collaborative (N3C)
Date of Completion: May 28, 2025
Disclosure Updated Date: August 23, 2024

ASN
American Society of Nephrology
JASN
Journal of the American Society of Nephrology
KIDNEY360
Accessing Our World From Every Angle
CJASN
Clinical Journal of the American Society of Nephrology

ASN Journal Disclosure Form

As per ASN journal policy, I have disclosed any financial relationships or commitments I have held in the past 36 months as included below. I have listed my Current Employer below to indicate there is a relationship requiring disclosure. If no relationship exists, my Current Employer is not listed.

Y. Han has nothing to disclose.

I understand that the information above will be published within the journal article, if accepted, and that failure to comply and/or to accurately and completely report the potential financial conflicts of interest could lead to the following: 1) Prior to publication, article rejection, or 2) Post-publication, sanctions ranging from, but not limited to, issuing a correction, reporting the inaccurate information to the authors' institution, banning authors from submitting work to ASN journals for varying lengths of time, and/or retraction of the published work.

Name: Yun Han
Manuscript ID: CJASN-2025-000130
Manuscript Title: The association of Long COVID and CKD: findings from the National COVID Cohort Collaborative (N3C)
Date of Completion: April 2, 2025
Disclosure Updated Date: April 2, 2025

ASN American Society of Nephrology
JASN Journal of the American Society of Nephrology
KIDNEY360 Accessing Our World From Every Angle
CJASN Clinical Journal of the American Society of Nephrology

ASN Journal Disclosure Form

As per ASN journal policy, I have disclosed any financial relationships or commitments I have held in the past 36 months as included below. I have listed my Current Employer below to indicate there is a relationship requiring disclosure. If no relationship exists, my Current Employer is not listed.

Y. He reports the following:
Employer: University of Michigan Medical School; Consultancy: INFOTECH Soft, Inc.;; and Advisory or Leadership Role: Editorial Board: Frontier in Cellular and Infection Microbiology, Frontiers in Bioinformatics and Computational Biology, and Scientific Data; 2022-2026: President (2022-2024), Board Chair (2024-2026), Overseas Chinese Society for Microbiology (Sino-Micro); 2017-present: Advisory committee member of China Biomedical Ontology Consortium (i.e., OntoChina). 2023-2024: Co-organizer for annual Vaccine/Drug Ontology Study (VDOS) Workshops. Note that these activities are all for non-profit purpose.

I understand that the information above will be published within the journal article, if accepted, and that failure to comply and/or to accurately and completely report the potential financial conflicts of interest could lead to the following: 1) Prior to publication, article rejection, or 2) Post-publication, sanctions ranging from, but not limited to, issuing a correction, reporting the inaccurate information to the authors' institution, banning authors from submitting work to ASN journals for varying lengths of time, and/or retraction of the published work.

Name: Yongqun Oliver He
Manuscript ID: CJASN-2025-000130R1
Manuscript Title: The association of Long COVID and CKD: findings from the National COVID Cohort Collaborative (N3C)
Date of Completion: June 15, 2025
Disclosure Updated Date: June 15, 2025

ASN American Society of Nephrology | JASN Journal of the American Society of Nephrology | KIDNEY360 Accessing Our World From Every Angle | CJASN Clinical Journal of the American Society of Nephrology

ASN Journal Disclosure Form

As per ASN journal policy, I have disclosed any financial relationships or commitments I have held in the past 36 months as included below. I have listed my Current Employer below to indicate there is a relationship requiring disclosure. If no relationship exists, my Current Employer is not listed.

S. Kane-Gill reports the following:
Employer: University of Pittsburgh; Research Funding: National Institute of Diabetes and Digestive and Kidney Diseases; National Center For Complementary and Alternative Medicine; Jewish Healthcare Foundation; and Advisory or Leadership Role: American Board of Internal Medicine- Critical Care Specialty Board.

I understand that the information above will be published within the journal article, if accepted, and that failure to comply and/or to accurately and completely report the potential financial conflicts of interest could lead to the following: 1) Prior to publication, article rejection, or 2) Post-publication, sanctions ranging from, but not limited to, issuing a correction, reporting the inaccurate information to the authors' institution, banning authors from submitting work to ASN journals for varying lengths of time, and/or retraction of the published work.

Name: Sandra L. Kane-Gill
Manuscript ID: CJASN-2025-000130R1
Manuscript Title: "The association of Long COVID and CKD: findings from the National COVID Cohort Collaborative (N3C),"
Date of Completion: May 2, 2025
Disclosure Updated Date: May 2, 2025
