# Supplementary material for: The Association of Long COVID and CKD: Findings from the National Clinical Cohort Collaborative
Source: Clin J Am Soc Nephrol. 2025 Aug 5;20(10):1323–40. doi: 10.2215/CJN.0000000773 (PMC12537286; doi:10.2215/CJN.0000000773)
Supplement: Supplementary file 2 [file cjasn-20-1323-s002.pdf]

Supplemental Tables:

Supplemental Methods Table: Defined list of concepts for visit type, race, ethnicity, rurality, Long COVID, CKD, diabetes mellitus, hypertension, cardiovascular disease, heart failure, ESKD, and AKI.

Supplemental Table 1: Descriptive Statistics of Patients with and without Baseline CKD/ESKD.

Supplemental Table 2: Univariate and Multivariable Cox Proportional Hazards Regression for Long COVID.

Supplemental Table 3: Descriptive Statistics of Patients with and without Baseline Mild CKD (Stage 3a).

Supplemental Table 4: Descriptive Statistics of Patients with and without Baseline Mild CKD (Stage 3a) After PSM.

Supplemental Table 5: Univariate and Multivariable Cox Proportional Hazards Regression for Long COVID (Mild CKD – Stage 3a).

Supplemental Table 6: Univariate and Multivariable Cox Proportional Hazards Regression for Long COVID After PSM (Mild CKD – Stage 3a).

Supplemental Table 7: Descriptive Statistics of Patients with and without Baseline Long COVID.

Supplemental Table 8: Univariate and Multivariable Cox Proportional Hazards Regression for Incident CKD/ESKD.

Supplemental Table 9: Descriptive Statistics for Patients with and without Long COVID (Among Patients with at Least 2 eGFR Measurements 30–365 Days Post COVID-19).

Supplemental Table 10: Descriptive Statistics for Patients with and without Long COVID After PSM (Among Patients with at Least 2 eGFR Measurements).

Supplemental Table 11: Univariate and Multivariable Cox Proportional Hazards Regression for eGFR Decline (Among Patients with at Least 2 eGFR Measurements).

Supplemental Table 12: Univariate and Multivariable Cox Proportional Hazards Regression for eGFR Decline after PSM (Among Patients with at Least 2 eGFR Measurements)

Sensitivity Analysis 1 (Alternative CKD Definition by the 2 GFR criteria):

Supplemental Table 13: Descriptive Statistics for Patients with and without CKD/ESKD after PSM (baseline CKD based on the 2 eGFR criteria - Sensitivity Analysis 1).

Supplemental Table 14: Univariate and Multivariable Cox Proportional Hazards Regression for Long COVID After PSM (Sensitivity Analysis 1).

Supplemental Table 15: Descriptive Statistics Among Patients with and without Long COVID after PSM (incident CKD based on the 2 eGFR criteria - Sensitivity Analysis 1).

Supplemental Table 16: Univariate and Multivariable Cox Proportional Hazards Regression for Incident CKD/ESKD After PSM (Sensitivity Analysis 1).

Supplemental Table 17: Descriptive Statistics of Patients with and without Long COVID After PSM (Among Patients with at Least 2 eGFR Measurements - Sensitivity Analysis 1).

Supplemental Table 18: Univariate and Multivariable Cox Proportional Hazards Regression for eGFR Decline After PSM (Sensitivity Analysis 1).

Sensitivity Analysis 2 (Adjustment for COVID-19 Severity):

Supplemental Table 19: Univariate and Multivariable Cox Proportional Hazards Regression for Long COVID Including Adjustment for Acute COVID-19 Severity After PSM (Sensitivity Analysis 2).

Supplemental Table 20: Univariate and Multivariable Cox Proportional Hazards Regression for Incident CKD/ESKD Including Adjustment for Acute COVID-19 Severity After PSM (Sensitivity Analysis 2).

Supplemental Table 21: Univariate and Multivariable Cox Proportional Hazards Regression for eGFR Decline Including Adjustment for Acute COVID-19 Severity After PSM (Sensitivity Analysis 2).

Sensitivity Analysis 3 (Age Stratification):

Supplemental Table 22: Univariate and Multivariable Cox Proportional Hazards Regression for Long COVID Among Patients Under 65 After PSM (Sensitivity Analysis 3).

Supplemental Table 23: Univariate and Multivariable Cox Proportional Hazards Regression for Long COVID Among Patients 65 and Older After PSM (Sensitivity Analysis 3).
Supplemental Table 24: Univariate and Multivariable Cox Proportional Hazards Regression for Incident CKD/ESKD Among Patients Under 65 After PSM (Sensitivity Analysis 3).
Supplemental Table 25: Univariate and Multivariable Cox Proportional Hazards Regression for Incident CKD/ESKD Among Patients 65 and Older After PSM (Sensitivity Analysis 3).
Supplemental Table 26: Univariate and Multivariable Cox Proportional Hazards Regression for eGFR Decline Among Patients Under 65 After PSM (Sensitivity Analysis 3).
Supplemental Table 27: Univariate and Multivariable Cox Proportional Hazards Regression for eGFR Decline Among Patients 65 and Older After PSM (Sensitivity Analysis 3).

Supplementary Methods Figure
Supplementary Figure 1. Competing Risk for Long COVID/Death
Supplementary Figure 2. Covariate Balance Plot After Matching for Long COVID as an Outcome
Supplementary Figure 3. Competing Risk for Long COVID/Death after PSM
Supplementary Figure 4. Cumulative Incidence for Long COVID Among Patients with and without a History of CKD Stage 3a
Supplementary Figure 5. Cumulative Incidence for Long COVID Among Patients with and without a History of CKD Stage 3a after PSM
Supplementary Figure 6. Competing Risk for Incident CKD/ESKD and Death
Supplementary Figure 7. Covariate Balance Plot After Matching for Incident CKD as an Outcome
Supplementary Figure 8. Competing Risk for Incident CKD/ESKD and Death After PSM
Supplementary Figure 9. Cumulative Incidence of eGFR Decline
Supplementary Figure 10. Competing Risk for eGFR Decline and Death
Supplementary Figure 11. Covariate Balance Plot After Matching for eGFR Decline as an Outcome
Supplementary Figure 12. Cumulative Incidence of eGFR Decline After PSM
Supplementary Figure 13. Competing Risk for eGFR Decline and Death After PSM

Supplemental Methods Table: Defined list of concepts for visit type, race, ethnicity, rurality, Long COVID, CKD, diabetes mellitus, hypertension, cardiovascular disease, heart failure, ESKD, and AKI

**Visit Type**	**Concept name**
**Inpatient**	
	inpatient visit
	inpatient hospital
	emergency room and inpatient visit
	inpatient intensive care unit
	inpatient psychiatric facility
**Visit Type**	**Concept name**
**Outpatient**	
	Outpatient Visit
	Office Visit
	Laboratory Visit
	Observation Room
	Non-hospital institution Visit
	Ambulatory Surgical Center
	Ambulatory Clinic / Center, Ambulatory Infusion Therapy Clinic / Center
	Interactive Telemedicine Service
	Telehealth
	Ambulatory Oncology Clinic / Center, Ambulatory Radiology Clinic / Center
	Telephone call to a patient
	Ambulatory Endoscopy Clinic / Center
	Ambulatory Oncological Radiation Clinic / Center
	Ambulatory Dental Clinic / Center
	Ambulatory Magnetic Resonance Imaging (MRI) Clinic / Center
	Skilled Nursing Facility
**Race**	**Concept name**
**White**	
	White
**Black**	
	Black
	Black or African American
**Asian**	
	Asian
	Asian Indian
	Filipino
	Korean
	Chinese
	Vietnamese
	Japanese
**American Indian or Alaska Native**	American Indian or Alaska Native
**Pacific Islander**	Native Hawaiian or Other Pacific Islander
	Polynesian
**Others**	
	other
	different races
	multiple races
	two or more races
**Ethnicity**	**Concept name**
**Not Hispanic or Latino Ethnicity**	
	Not Hispanic or Latino Ethnicity
**Hispanic or Latino**	
	Hispanic or Latino

**Rurality**	Rural-Urban Commuting Area codes[3,7]
**Long COVID**	Post COVID-19 condition, unspecified (ICD-10 code: U09.9)
	**Diagnostic concept name**
**Chronic Kidney Disease (CKD)**	Chronic kidney disease stage 3<br>Chronic kidney disease due to type 2 diabetes mellitus<br>Chronic kidney disease<br>Chronic kidney disease due to hypertension<br>Anemia in chronic kidney disease<br>Chronic kidney disease stage 4<br>Hypertensive heart and renal disease with (congestive) heart failure<br>Renal disorder due to type 2 diabetes mellitus<br>Hypertensive renal failure<br>Chronic kidney disease stage 2<br>Chronic kidney disease stage 5<br>Chronic kidney disease stage 3A<br>Hypertensive heart and chronic kidney disease<br>Chronic kidney disease stage 3B<br>Renal disorder due to type 1 diabetes mellitus<br>Disorder of kidney due to diabetes mellitus<br>SLE glomerulonephritis syndrome<br>Nephritic syndrome<br>Chronic kidney disease stage 1<br>Multiple congenital cysts of kidney<br>Nephrotic syndrome<br>Hypertensive renal disease<br>Benign hypertensive renal disease with renal failure<br>Impaired renal function disorder<br>Chronic kidney disease stage 5 due to hypertension<br>Hypertensive heart AND renal disease<br>Hypertensive heart AND chronic kidney disease stage 5<br>Membranous glomerulonephritis<br>Renal impairment<br>Renal insufficiency<br>Nephrotic syndrome, focal and segmental glomerular lesions<br>Aneurysm of renal artery<br>Recurrent hematuria co-occurrent and due to diffuse membranous glomerulonephritis<br>Alport syndrome<br>Anemia of chronic renal failure<br>Mesangiocapillary glomerulonephritis<br>Chronic nephritic syndrome<br>Nephrotic syndrome, minor glomerular abnormality<br>Chronic nephritic syndrome with membranous glomerulonephritis<br>Chronic renal failure<br>Polycystic kidney disease, infantile type<br>Postoperative renal failure<br>Focal segmental glomerulosclerosis<br>Nephrotic syndrome, diffuse membranous glomerulonephritis<br>Hypertensive heart AND chronic kidney disease with congestive heart failure<br>Pre-existing hypertensive chronic kidney disease in mother complicating pregnancy<br>Chronic kidney disease due to type 1 diabetes mellitus<br>Congenital renal failure<br>Renal disease in pregnancy AND/OR puerperium without hypertension<br>Acute-on-chronic renal failure<br>Chronic renal insufficiency<br>Diffuse crescentic glomerulonephritis<br>IgA nephropathy<br>Nephrotic syndrome with minimal change glomerulonephritis<br>Anemia in chronic kidney disease stage 4<br>Chronic kidney disease stage 5 on dialysis<br>Nephrotic syndrome associated with another disorder<br>Nephrotic syndrome with membranous glomerulonephritis

	Chronic kidney disease mineral and bone disorder<br>Anemia co-occurrent and due to chronic kidney disease stage 3<br>Chronic nephritic syndrome, focal and segmental glomerular lesions<br>Chronic kidney disease stage 3 due to type 2 diabetes mellitus<br>Benign hypertensive heart AND renal disease<br>Nephrotic syndrome, diffuse mesangiocapillary glomerulonephritis<br>Hereditary nephritis<br>Chronic nephritic syndrome, diffuse mesangiocapillary glomerulonephritis<br>Glomerulonephritis<br>Microalbuminuric diabetic nephropathy<br>Isolated proteinuria with specified morphological lesion, diffuse membranous glomerulonephritis<br>Microalbuminuria due to type 2 diabetes mellitus<br>Chronic glomerulonephritis<br>Chronic kidney disease stage 4 due to type 2 diabetes mellitus<br>Nephritis<br>Minimal change disease<br>Malignant hypertensive chronic kidney disease<br>Chronic mesangial proliferative glomerulonephritis<br>Complement component 3 glomerulonephritis<br>Hypertensive heart AND chronic kidney disease stage 3<br>Chronic kidney disease stage 3 due to hypertension<br>Dense deposit disease<br>Chronic kidney disease stage 5 due to type 2 diabetes mellitus<br>Malignant hypertensive renal disease<br>SLE glomerulonephritis syndrome, WHO class V<br>Cardiorenal syndrome<br>Mesangial proliferative glomerulonephritis<br>Glomerulosclerosis<br>Proteinuric nephropathy due to diabetes mellitus<br>SLE glomerulonephritis syndrome, WHO class IV<br>Nephrotic syndrome, diffuse mesangial proliferative glomerulonephritis<br>Benign hypertensive renal disease<br>Proteinuria due to type 2 diabetes mellitus<br>Isolated proteinuria with specified morphological lesion, diffuse mesangial proliferative glomerulon…<br>Nephrotic syndrome with membranoproliferative glomerulonephritis<br>Chronic kidney disease stage 4 due to hypertension<br>Steroid-resistant nephrotic syndrome<br>Recurrent hematuria co-occurrent and due to diffuse mesangiocapillary glomerulonephritis<br>Glomerulonephritis co-occurrent and due to antineutrophil cytoplasmic antibody positive vasculitis<br>Page kidney<br>Anemia in chronic kidney disease stage 5<br>Nephrotic syndrome, diffuse crescentic glomerulonephritis<br>Congenital nephrotic syndrome<br>Pre-existing hypertensive heart and chronic kidney disease in mother complicating pregnancy<br>Glomerulopathy due to diabetes mellitus<br>Nephrotic syndrome, dense deposit disease<br>Anemia, pre-end stage renal disease on erythropoietin protocol<br>Malignant hypertensive heart AND renal disease<br>Chronic kidney disease stage 3 due to type 1 diabetes mellitus<br>Chronic kidney disease stage 2 due to type 2 diabetes mellitus<br>Microalbuminuria due to type 1 diabetes mellitus<br>Hereditary diffuse membranous glomerulonephritis<br>Idiopathic crescentic glomerulonephritis<br>Steroid-dependent nephrotic syndrome<br>SLE glomerulonephritis syndrome, WHO class III<br>Isolated proteinuria with specified morphological lesion, diffuse endocapillary proliferative glomer…<br>Isolated proteinuria with specified morphological lesion, diffuse concentric glomerulonephritis<br>Non-functioning kidney<br>Macroalbuminuric nephropathy due to diabetes mellitus<br>Hereditary diffuse mesangial proliferative glomerulonephritis<br>Chronic nephritic syndrome, diffuse crescentic glomerulonephritis<br>Nephrotic syndrome in amyloidosis<br>Type 2 diabetes mellitus with diabetic chronic kidney disease<br>Recurrent hematuria co-occurrent and due to diffuse crescentic glomerulonephritis<br>Drash syndrome<br>Mesangiocapillary glomerulonephritis, type I<br>Malignant hypertensive chronic kidney disease stage 1

	Focal glomerular sclerosis<br>Pre-existing hypertensive heart and chronic kidney disease in mother complicating childbirth<br>Immune-complex glomerulonephritis<br>Primary IgA nephropathy<br>Immunotactoid glomerulonephritis<br>Chronic progressive renal failure<br>Fibrillary glomerulonephritis<br>Hypertension in chronic kidney disease stage 3 due to type 2 diabetes mellitus<br>Chronic kidney disease stage 4 due to type 1 diabetes mellitus<br>Chronic glomerulonephritis associated with another disorder<br>Glomerulonephritis due to granulomatosis with polyangiitis<br>Hypertension in chronic kidney disease due to type 2 diabetes mellitus<br>SLE glomerulonephritis syndrome, WHO class I<br>Proliferative glomerulonephritis<br>Chronic proliferative glomerulonephritis<br>Persistent hematuria co-occurrent and due to diffuse endocapillary proliferative glomerulonephritis<br>Chronic nephritic syndrome, dense deposit disease<br>Chronic kidney disease stage 1 due to type 2 diabetes mellitus<br>Chronic nephritic syndrome, minor glomerular abnormality<br>SLE glomerulonephritis syndrome, WHO class II<br>Chronic nephritic syndrome, diffuse endocapillary proliferative glomerulonephritis<br>Persistent microalbuminuria due to type 2 diabetes mellitus<br>C1q nephropathy<br>Hypertensive heart AND chronic kidney disease stage 4<br>Nephrotic syndrome, diffuse endocapillary proliferative glomerulonephritis<br>Nephrotic syndrome due to diabetes mellitus<br>Aneurysm of left renal artery<br>Mesangiocapillary glomerulonephritis, type II<br>Diffuse endocapillary proliferative glomerulonephritis<br>Nephrotic syndrome with proliferative glomerulonephritis<br>Idiopathic glomerulonephritis<br>Chronic kidney disease stage 5 due to type 1 diabetes mellitus<br>Nephrotic-nephritic syndrome<br>Aneurysm of right renal artery<br>Anti-glomerular basement membrane disease<br>Hypertension in chronic kidney disease stage 5 due to type 2 diabetes mellitus<br>Chronic kidney disease stage 1 due to type 1 diabetes mellitus<br>Chronic kidney disease stage 3 due to benign hypertension<br>Hypertensive heart AND chronic kidney disease stage 2<br>Glomerulitis<br>Hereditary diffuse mesangiocapillary glomerulonephritis<br>Hypertension in chronic kidney disease stage 4 due to type 2 diabetes mellitus<br>Hypertension in chronic kidney disease due to type 1 diabetes mellitus<br>Focal AND segmental proliferative glomerulonephritis<br>Chronic kidney disease stage 2 due to type 1 diabetes mellitus<br>Finnish congenital nephrotic syndrome<br>Alport syndrome X-linked<br>Idiopathic membranous glomerulonephritis<br>Necrotizing glomerulonephritis<br>IgM nephropathy<br>Hypertensive renal disease complicating AND/OR reason for care during pregnancy<br>Sclerosing glomerulonephritis<br>Persistent proteinuria due to type 2 diabetes mellitus<br>Glomerulosclerosis of kidney due to diabetes mellitus<br>Glomerulonephritis due to Henoch-Schönlein purpura<br>Milk alkali syndrome<br>Armanni-Ebstein kidney due to diabetes mellitus<br>Acute glomerulonephritis associated with another disorder<br>Chronic kidney disease following donor nephrectomy<br>Hypertension in chronic kidney disease stage 2 due to type 2 diabetes mellitus<br>Nephrotic syndrome due to type 2 diabetes mellitus<br>Steroid-sensitive nephrotic syndrome<br>De novo glomerulonephritis<br>Malignant arteriolar nephrosclerosis<br>Membranous glomerulonephritis - stage I<br>Nephrotic syndrome secondary to systemic disease<br>Persistent hematuria co-occurrent and due to diffuse crescentic glomerulonephritis

	Anti-glomerular basement membrane tubulointerstitial nephritis<br>Cardiovascular renal disease<br>SLE glomerulonephritis syndrome, WHO class VI<br>Chronic kidney disease due to benign hypertension<br>Proteinuria due to type 1 diabetes mellitus<br>Glomerulonephritis due to hepatitis C<br>Interstitial nephritis<br>Kimmelstiel-Wilson syndrome<br>Recurrent hematuria co-occurrent and due to dense deposit disease<br>Malignant hypertensive chronic kidney disease stage 2<br>Malignant hypertensive chronic kidney disease stage 3<br>Alport syndrome, intellectual disability, midface hypoplasia, elliptocytosis syndrome<br>Chronic kidney disease stage 2 due to benign hypertension<br>Chronic kidney disease stage 2 due to hypertension<br>Chronic diffuse glomerulonephritis<br>Chronic kidney disease stage 4 due to benign hypertension<br>IgA nephropathy associated with liver disease<br>Persistent hematuria co-occurrent and due to dense deposit disease<br>Sarcoidosis with glomerulonephritis<br>Chronic kidney disease stage 5 with transplant<br>Epstein syndrome<br>Congenital renal artery aneurysm<br>Chronic kidney disease stage 1 due to hypertension<br>Schimke immuno-osseous dysplasia<br>Hereditary diffuse endocapillary proliferative glomerulonephritis<br>Persistent microalbuminuria due to type 1 diabetes mellitus<br>Complement component 3 glomerulopathy<br>Shunt nephritis<br>Hypertensive heart AND chronic kidney disease stage 1<br>Alport syndrome autosomal dominant<br>Mesangiocapillary glomerulonephritis type III<br>Pre-existing hypertensive heart and renal disease complicating pregnancy, childbirth and the puerper…<br>Benign hypertensive heart disease and chronic renal disease stage 3<br>Autosomal dominant focal segmental glomerulosclerosis<br>Steroid sensitive nephrotic syndrome of childhood<br>Benign hypertensive heart disease and chronic renal disease stage 4<br>Galloway Mowat syndrome<br>Acquired renal artery aneurysm<br>Chronic kidney disease stage 1 due to benign hypertension<br>Membranous glomerulonephritis - stage II<br>Hereditary diffuse crescentic glomerulonephritis<br>Steroid-resistant minimal change glomerulonephritis<br>Malignant hypertensive chronic kidney disease stage 5<br>Nephrotic syndrome secondary to glomerulonephritis<br>Membranous glomerulonephritis - stage III<br>Hypertensive chronic kidney disease with stage 1 through stage 4 chronic kidney disease, or unspecif…<br>Hypertensive heart and chronic kidney disease with heart failure and stage 1 through stage 4 chronic…<br>Hypertensive chronic kidney disease with stage 5 chronic kidney disease or end stage renal disease<br>Hypertensive heart and chronic kidney disease with heart failure and with stage 5 chronic kidney dis…<br>Type 1 diabetes mellitus with diabetic chronic kidney disease<br>Erythropoietin resistance in anemia of chronic kidney disease<br>Hypertensive heart and chronic kidney disease without heart failure, with stage 5 chronic kidney dis…<br>Isolated proteinuria with specified morphological lesion, diffuse mesangiocapillary glomerulonephrit…<br>Hyperuricemia, anemia, renal failure syndrome<br>Adult type polycystic kidney disease type 1<br>Acquired polycystic kidney disease
**Acute Kidney Injury (AKI)**	
	Acute injury of kidney
	Acute tubular necrosis
	Hepatorenal syndrome
	Acute renal failure due to acute cortical necrosis
	Hemolytic uremic syndrome
	Acute-on-chronic renal failure
	Acute renal insufficiency

	Postpartum acute renal failure
	Acute nephritis
	Rapidly progressive nephritic syndrome
	Acute renal impairment
	Acute kidney injury due to sepsis
	Acute tubulointerstitial nephritis
	Rapidly progressive nephritic syndrome
	diffuse crescentic glomerulonephritis
	Acute nontraumatic kidney injury,
	Acute renal failure on dialysis
	Acute kidney failure stage 1-3
	Rapidly progressive glomerulonephritis
	Hemorrhagic fever with renal syndrome
	Acute renal cortical necrosis
	Rapidly progressive nephritic syndrome
	diffuse membranous glomerulonephritis
	Acute renal failure due to tubular necrosis
	Acute kidney injury due to hypovolemia.
	**Diagnostic concept name**
**Diabetes Mellitus (DM)**	
	Type 2 diabetes mellitus
	Type 2 diabetes mellitus without complication
	Hyperglycemia due to type 2 diabetes mellitus
	Chronic kidney disease due to type 2 diabetes mellitus
	Complication due to diabetes mellitus
	Polyneuropathy due to type 2 diabetes mellitus
	Disorder of nervous system due to type 2 diabetes mellitus
	Peripheral circulatory disorder due to type 2 diabetes mellitus
	Foot ulcer due to type 2 diabetes mellitus
	Renal disorder due to type 2 diabetes mellitus
	Type 1 diabetes mellitus
	Secondary diabetes mellitus
	Disorder due to type 2 diabetes mellitus
	Diabetic ketoacidosis without coma
	Proliferative retinopathy due to type 2 diabetes mellitus
	Diabetes mellitus without complication
	Hyperglycemia due to type 1 diabetes mellitus
	Disorder of eye due to type 2 diabetes mellitus
	Hypoglycemia due to type 2 diabetes mellitus
	Type 2 diabetes mellitus with ulcer
	Diabetes mellitus
	Mild nonproliferative retinopathy due to type 2 diabetes mellitus
	Type 1 diabetes mellitus without complication
	Macular edema due to diabetes mellitus
	Autonomic neuropathy due to type 2 diabetes mellitus
	Gestational diabetes mellitus
	Macular edema and retinopathy due to type 2 diabetes mellitus
	Disorder of nervous system due to diabetes mellitus
	Insulin treated type 2 diabetes mellitus
	Polyneuropathy due to diabetes mellitus
	Disorder of kidney due to diabetes mellitus
	Dermopathy due to type 2 diabetes mellitus
	Moderate nonproliferative retinopathy due to type 2 diabetes mellitus
	Drug-induced diabetes mellitus
	Type II diabetes mellitus uncontrolled
	Renal disorder due to type 1 diabetes mellitus
	Gangrene due to type 2 diabetes mellitus
	Peripheral angiopathy due to diabetes mellitus
	Pre-existing type 2 diabetes mellitus in pregnancy
	Cataract due to diabetes mellitus type 2
	Diabetic foot ulcer
	Disorder of eye due to diabetes mellitus

	Neuropathic arthropathy due to type 2 diabetes mellitus
	Diabetes insipidus
	Hypoglycemia due to type 1 diabetes mellitus
	Disorder of nervous system due to type 1 diabetes mellitus
	Pre-existing type 1 diabetes mellitus in pregnancy
	Autonomic neuropathy due to type 1 diabetes mellitus
	Polyneuropathy due to type 1 diabetes mellitus
	Type 2 diabetes mellitus well controlled
	Nonproliferative retinopathy due to type 2 diabetes mellitus
	Proliferative retinopathy due to diabetes mellitus
	Retinopathy due to type 2 diabetes mellitus
	Neuropathy due to diabetes mellitus
	Ulcer of lower limb due to type 1 diabetes mellitus
	Mononeuropathy due to type 2 diabetes mellitus
	Neuropathy due to type 2 diabetes mellitus
	Severe hyperglycemia due to diabetes mellitus
	Type 2 diabetes mellitus with peripheral angiopathy
	Peripheral vascular disorder due to diabetes mellitus
	Severe nonproliferative retinopathy with clinically significant macular edema due to diabetes mellitus
	Retinopathy due to type 1 diabetes mellitus
	Proliferative retinopathy due to type 1 diabetes mellitus
	Mild nonproliferative retinopathy due to diabetes mellitus
	Pre-existing diabetes mellitus in pregnancy
	Moderate nonproliferative retinopathy due to diabetes mellitus
	Diabetes mellitus during pregnancy, childbirth and the puerperium
	Type 1 diabetes mellitus uncontrolled
	Ketoacidosis due to type 2 diabetes mellitus
	Severe nonproliferative retinopathy without macular edema due to diabetes mellitus
	Autonomic neuropathy due to diabetes mellitus
	Retinopathy due to diabetes mellitus
	Nonproliferative retinopathy due to diabetes mellitus
	Ketoacidosis due to type 1 diabetes mellitus
	Peripheral circulatory disorder due to type 1 diabetes mellitus
	Hyperosmolar coma due to diabetes mellitus
	Hyperosmolar coma due to type 2 diabetes mellitus
	Diabetic ketoacidosis
	Latent autoimmune diabetes mellitus in adult
	Disorder due to type 1 diabetes mellitus
	Severe nonproliferative retinopathy due to diabetes mellitus
	Lumbosacral radiculoplexus neuropathy due to type 2 diabetes mellitus
	Arthropathy due to type 2 diabetes mellitus
	Diabetes mellitus in mother complicating pregnancy, childbirth AND/OR puerperium
	Disorder of eye due to type 1 diabetes mellitus
	Mild nonproliferative retinopathy due to type 1 diabetes mellitus
	Type 2 diabetes mellitus in obese
	Gestational diabetes mellitus in childbirth
	O/E - right eye proliferative diabetic retinopathy
	Infection of foot due to diabetes mellitus
	Proliferative retinopathy of right eye with diabetes mellitus
	Type 1 diabetes mellitus with ulcer
	Gastroparesis due to diabetes mellitus
	O/E - left eye proliferative diabetic retinopathy
	Cataract due to diabetes mellitus type 1
	Mild nonproliferative retinopathy of right eye due to diabetes mellitus
	Mild nonproliferative retinopathy of left eye due to diabetes mellitus
	Moderate nonproliferative retinopathy due to type 1 diabetes mellitus
	Traction detachment of retina due to type 2 diabetes mellitus
	Diabetic mononeuropathy
	Hypoglycemia due to diabetes mellitus
	Skin ulcer due to diabetes mellitus
	Nonproliferative diabetic retinopathy due to type 1 diabetes mellitus
	Pre-existing diabetes mellitus in mother complicating childbirth
	Nephrogenic diabetes insipidus
	Ketoacidotic coma due to type 1 diabetes mellitus

	Diabetic neuropathy with neurologic complication
	Diabetes mellitus type 2 without retinopathy
	Type 2 diabetes mellitus in nonobese
	Dermopathy due to type 1 diabetes mellitus
	Macular edema due to type 2 diabetes mellitus
	Cataract due to diabetes mellitus
	Ulcer of midfoot due to diabetes mellitus
	Hyperosmolar coma due to secondary diabetes mellitus
	Mononeuropathy due to type 1 diabetes mellitus
	Ulcer of toe due to type 2 diabetes mellitus
	Chronic kidney disease due to type 1 diabetes mellitus
	Moderate nonproliferative diabetic retinopathy of right eye
	Diabetes mellitus during pregnancy - baby not yet delivered
	Hypoglycemic coma due to type 2 diabetes mellitus
	Ketoacidotic coma due to type 2 diabetes mellitus
	Steroid-induced diabetes
	Neuropathic arthropathy due to type 1 diabetes mellitus
	Gestational diabetes mellitus, class A>2<
	Moderate nonproliferative diabetic retinopathy of left eye
	Dyslipidemia due to type 2 diabetes mellitus
	Ketoacidotic coma due to diabetes mellitus
	Coma due to diabetes mellitus
	Type 2 diabetes mellitus controlled by diet
	Chronic kidney disease stage 3 due to type 2 diabetes mellitus
	Chronic kidney disease stage 3 due to type 1 diabetes mellitus
	Mixed hyperlipidemia due to type 2 diabetes mellitus
	Gangrene due to type 1 diabetes mellitus
	Gestational diabetes mellitus complicating pregnancy
	Pregnancy and type 2 diabetes mellitus
	Posttransplant diabetes mellitus
	Pregnancy and type 1 diabetes mellitus
	Diabetic peripheral neuropathy
	Proliferative retinopathy of left eye due to diabetes mellitus
	Hypoglycemic coma due to type 1 diabetes mellitus
	Diabetic dermopathy
	Ulcer of heel due to diabetes mellitus
	Disorder due to well controlled type 2 diabetes mellitus
	Gestational diabetes mellitus, class A>1<
	Macular edema of right eye due to diabetes mellitus
	Diabetic foot
	Lumbosacral radiculoplexus neuropathy due to diabetes mellitus
	Proteinuric nephropathy due to diabetes mellitus
	Blindness due to type 1 diabetes mellitus
	Lesion of skin due to diabetes mellitus
	Pre-existing type 1 diabetes mellitus
	Neuropathic arthropathy due to diabetes mellitus
	Chronic kidney disease stage 2 due to type 2 diabetes mellitus
	Peripheral neuropathy due to type 2 diabetes mellitus
	Moderate nonproliferative retinopathy due to secondary diabetes mellitus
	Gangrene due to diabetes mellitus
	Hyperosmolar non-ketotic state due to type 2 diabetes mellitus
	Severe nonproliferative retinopathy of left eye due to diabetes mellitus
	Ulcer of heel due to type 2 diabetes mellitus
	Severe nonproliferative retinopathy of right eye due to diabetes mellitus
	Chronic kidney disease stage 4 due to type 2 diabetes mellitus
	Hyperlipidemia due to type 2 diabetes mellitus
	Postpartum gestational diabetes mellitus
	Vitreous hemorrhage due to diabetes mellitus
	Vitreous hemorrhage of left eye due to diabetes mellitus
	Mixed hyperlipidemia due to type 1 diabetes mellitus
	Diabetes mellitus in mother complicating childbirth
	Chronic kidney disease stage 4 due to type 1 diabetes mellitus
	Pre-existing type 2 diabetes mellitus
	Erectile dysfunction due to type 2 diabetes mellitus

	Mild nonproliferative retinopathy due to secondary diabetes mellitus
	Macroalbuminuric nephropathy due to diabetes mellitus
	Hypoglycemic coma due to diabetes mellitus
	Traction detachment of retina due to type 1 diabetes mellitus
	Microalbuminuria due to type 2 diabetes mellitus
	Lumbosacral radiculoplexus neuropathy due to type 1 diabetes mellitus
	Hyperglycemia due to diabetes mellitus
	Disorder of soft tissue due to diabetes mellitus
	Nonproliferative retinopathy of left eye due to diabetes mellitus
	Ulcer of right foot due to type 2 diabetes mellitus
	Abnormal metabolic state due to diabetes mellitus
	Hyperglycemic crisis due to diabetes mellitus
	Macular edema of left eye due to diabetes mellitus
	O/E - right eye stable treated proliferative diabetic retinopathy
	Peripheral angiopathy due to type 1 diabetes mellitus
	Proteinuria due to type 2 diabetes mellitus
	Ulcer of left foot due to type 2 diabetes mellitus
	Microalbuminuric diabetic nephropathy
	Chronic kidney disease stage 1 due to type 2 diabetes mellitus
	Ulcer of lower limb due to type 2 diabetes mellitus
	Diabetes mellitus associated with cystic fibrosis
	O/E - left eye stable treated proliferative diabetic retinopathy
	Cellulitis of foot due to diabetes mellitus
	Type 1 diabetes mellitus with arthropathy
	Glomerulopathy due to diabetes mellitus
	Hyperosmolar hyperglycemic coma due to diabetes mellitus without ketoacidosis
	Chronic painful neuropathy due to diabetes mellitus
	Diarrhea due to diabetes mellitus
	Skin ulcer of toe due to diabetes mellitus type 1
	Hyperosmolar coma due to type 1 diabetes mellitus
	Diabetes mellitus associated with hormonal etiology
	Retinopathy due to secondary diabetes mellitus
	Dermatitis due to drug induced diabetes mellitus
	Ulcer of foot due to type 1 diabetes mellitus
	Insulin dependent diabetes mellitus type 1A
	Acidosis due to type 2 diabetes mellitus
	Diabetes mellitus induced by non-steroid drugs
	Chronic kidney disease stage 5 due to type 2 diabetes mellitus
	Bullosis diabeticorum
	Hyperosmolar non-ketotic state due to diabetes mellitus
	Peripheral sensory neuropathy due to type 2 diabetes mellitus
	Skin ulcer due to type 2 diabetes mellitus
	Nephrotic syndrome due to diabetes mellitus
	Maturity-onset diabetes of the young, type 5
	Diabetes mellitus due to cystic fibrosis
	Microalbuminuria due to type 1 diabetes mellitus
	Diabetes mellitus in the puerperium - baby delivered during previous episode of care
	Diabetes mellitus associated with pancreatic disease
	Hypertension in chronic kidney disease stage 3 due to type 2 diabetes mellitus
	Hypoglycemic event due to diabetes
	O/E - left eye background diabetic retinopathy
	Coronary artery disease due to type 2 diabetes mellitus
	Glaucoma due to type 2 diabetes mellitus
	**Diagnostic concept name**
**Hypertension (HTN)**	
	Essential hypertension
	Hypertensive heart failure
	Hypertensive heart and renal disease with (congestive) heart failure
	Benign essential hypertension
	Hypertensive urgency
	Hypertensive disorder
	Hypertensive heart disease without congestive heart failure
	Hypertensive emergency

	Hypertensive heart disease with congestive heart failure
	Hypertensive heart and chronic kidney disease
	Hypertensive renal disease
	Hypertensive heart AND renal disease
	Hypertensive retinopathy
	Benign hypertensive renal disease with renal failure
	Hypertensive heart disease
	Benign hypertension
	Hypertensive encephalopathy
	Hypertensive renal failure
	Malignant essential hypertension
	Hypertensive crisis
	Hypertensive heart AND chronic kidney disease with congestive heart failure
	Hypertensive heart and renal disease with renal failure
	Benign hypertensive heart disease without congestive heart failure
	Benign hypertensive renal disease
	Benign hypertensive heart AND renal disease
	Hypertensive heart AND chronic kidney disease stage 5
	Malignant hypertension
	Benign hypertensive heart disease with congestive cardiac failure
	Blind hypertensive eye
	Hypertensive heart AND chronic kidney disease stage 3
	Pre-existing hypertensive chronic kidney disease in mother complicating pregnancy
	Malignant hypertensive heart disease without congestive heart failure
	Pre-existing hypertensive heart disease complicating pregnancy, childbirth and the puerperium
	Hypertensive left ventricular hypertrophy
	Hypertensive complication
	Hypertensive heart AND chronic kidney disease stage 4
	Malignant hypertensive heart AND renal disease
	Pre-existing hypertensive heart disease in mother complicating pregnancy
	Labile essential hypertension
	Benign hypertensive heart disease
	Resistant hypertensive disorder
	Pre-existing hypertensive heart and chronic kidney disease in mother complicating childbirth
	Hypertensive heart AND chronic kidney disease stage 2
	Pre-existing hypertensive heart and chronic kidney disease in mother complicating pregnancy
	Hypertensive nephrosclerosis
	Hypertensive heart and renal disease with both (congestive) heart failure and renal failure
	Pre-existing hypertensive heart and renal disease complicating pregnancy, childbirth and the puerperium
	Malignant hypertensive heart disease
	**Diagnostic concept name**
**Cardiovascular disease (CVD)**	
	Congestive heart failure
	Atherosclerosis of coronary artery without angina pectoris
	Atrial fibrillation
	Paroxysmal atrial fibrillation
	Heart failure
	Chronic systolic heart failure
	Chronic diastolic heart failure
	Chronic congestive heart failure
	Hypertensive heart failure
	Chronic atrial fibrillation
	Cardiomyopathy
	Hypertensive heart and renal disease with (congestive) heart failure
	Old myocardial infarction
	Generalized ischemic myocardial dysfunction
	Acute on chronic diastolic heart failure
	Cardiomegaly
	Acute on chronic systolic heart failure
	Non-rheumatic aortic sclerosis
	Chronic combined systolic and diastolic heart failure
	Cardiac arrhythmia

	Diastolic heart failure
	Persistent atrial fibrillation
	Acute non-ST segment elevation myocardial infarction
	Ventricular tachycardia
	Atrial flutter
	Non-rheumatic mitral valve stenosis with regurgitation
	Angina co-occurrent and due to coronary arteriosclerosis
	Supraventricular tachycardia
	Sick sinus syndrome
	Paralytic syndrome on one side of the body as late effect of cerebrovascular accident
	Acute on chronic combined systolic and diastolic heart failure
	Coronary arteriosclerosis
	Dilated cardiomyopathy
	Angina pectoris
	Systolic heart failure
	Ventricular premature complex
	Heart disease
	Right bundle branch block
	Coronary atherosclerosis
	Complete atrioventricular block
	Left bundle branch block
	First degree atrioventricular block
	Arteriosclerosis of coronary artery bypass graft
	Aortic incompetence, non-rheumatic
	Acute systolic heart failure
	Acute ST segment elevation myocardial infarction
	Tricuspid incompetence, non-rheumatic
	Acute ischemic heart disease
	Acute diastolic heart failure
	Cardiac transplant disorder
	Permanent atrial fibrillation
	Chronic ischemic heart disease
	Long QT syndrome
	Unstable angina co-occurrent and due to coronary arteriosclerosis
	Atrial premature complex
	Hypertensive heart disease without congestive heart failure
	Non-rheumatic heart valve disorder
	Rheumatic tricuspid valve regurgitation
	Cardiomyopathy associated with another disorder
	Typical atrial flutter
	Rheumatic disease of heart valve
	Late effects of cerebrovascular disease
	Preinfarction syndrome
	Second degree atrioventricular block
	Residual cognitive deficit as late effect of cerebrovascular accident
	Cardiac transplant rejection
	Mural thrombus of heart
	Hypertrophic cardiomyopathy
	Myocardial infarction due to demand ischemia
	Atrial septal defect
	Disorders of both mitral and tricuspid valves
	Aphasia as late effect of cerebrovascular disease
	Cardiac arrest
	Right ventricular failure
	Non-rheumatic mitral valve disease
	Atypical atrial flutter
	Conduction disorder of the heart
	Biventricular congestive heart failure
	Hypertensive heart disease with congestive heart failure
	Rheumatic disease of mitral AND aortic valves
	Valvular endocarditis
	Atrioventricular block
	Primary cardiomyopathy
	Dysphagia as a late effect of cerebrovascular accident

	Acute exacerbation of chronic congestive heart failure
	Hypertrophic obstructive cardiomyopathy
	Rheumatic disease of mitral valve
	Left anterior fascicular block
	Left heart failure
	Hypertensive heart and chronic kidney disease
	Bifascicular block
	Acute combined systolic and diastolic heart failure
	Combined disorders of mitral, aortic and tricuspid valves
	Ventricular fibrillation
	Chronic pulmonary heart disease
	Coronary arteriosclerosis in artery of transplanted heart
	Longstanding persistent atrial fibrillation
	Aortic valve disorder
	Acute myocardial infarction
	Congenital heart disease
	Paroxysmal ventricular tachycardia
	Infective endocarditis
	Myocardial infarction
	Acute cor pulmonale
	Mitral valve prolapse
	Hypertensive heart AND renal disease
	Takotsubo cardiomyopathy
	Congenital insufficiency of aortic valve
	Acute subendocardial infarction
	Dysarthria as late effects of cerebrovascular disease
	Mitral valve disorder
	Weakness of face muscles as sequela of stroke
	Chronic right-sided heart failure
	Sinus node dysfunction
	Rheumatic mitral stenosis
	Chronic total occlusion of coronary artery
	Pulmonary incompetence, non-rheumatic
	Non-rheumatic mitral regurgitation
	Aortic stenosis, non-rheumatic
	Acute on chronic right-sided congestive heart failure
	Chronic cor pulmonale
	Tetralogy of Fallot
	Acute ST segment elevation myocardial infarction involving left anterior descending coronary artery
	Hypertensive heart disease
	Ischemic myocardial dysfunction
	Intraventricular conduction defect
	Non-rheumatic mitral valve stenosis
	Sarcoid heart muscle disease
	Rheumatic heart disease
	Aortic valve stenosis
	Acute ST segment elevation myocardial infarction due to right coronary artery occlusion
	Congenital anomaly of coronary artery
	Ventricular tachyarrhythmia
	Coronary artery spasm
	Acute congestive heart failure
	Saddle embolus of pulmonary artery with acute cor pulmonale
	Heart failure with normal ejection fraction
	Multiple valve disease
	Cardiac transplant failure
	Heart block
	Ventricular septal defect
	Aortic valve stenosis with insufficiency
	Speech and language deficit as late effect of cerebrovascular accident
	Atrial fibrillation with rapid ventricular response
	Cardiovascular symptoms
	Disorders of both aortic and tricuspid valves
	Paroxysmal supraventricular tachycardia
	Arteriosclerosis of autologous vein coronary artery bypass graft

	Aortic valve regurgitation
	Restrictive cardiomyopathy
	Dilated cardiomyopathy secondary to peripartum heart disease
	Ataxia as sequela of cerebrovascular disease
	Dilated cardiomyopathy secondary to alcohol
	Acute right-sided heart failure
	Coronary thrombosis not resulting in myocardial infarction
	Pulmonary heart disease
	Dissection of coronary artery
	Premature beats
	Ventricular premature beats
	Discordant ventriculoarterial connection
	Mitral valve regurgitation
	Congestive heart failure due to left ventricular systolic dysfunction
	Symptomatic congestive heart failure
	Aneurysm of heart
	Cardiac tamponade
	Ebstein's anomaly
	Right heart failure secondary to left heart failure
	Aberrant premature complexes
	Accelerated atrioventricular conduction
	Paroxysmal tachycardia
	Myocarditis
	Chronic heart failure
	Disorder of transplanted heart
	Rheumatic mitral regurgitation
	Atrioventricular septal defect and common atrioventricular junction
	Rheumatic disease of tricuspid valve
	Disorder of coronary artery
	Hemiplegia of nondominant side as late effect of cerebrovascular disease
	Acute and subacute endocarditis
	Rheumatic mitral stenosis with regurgitation
	Chronic Chagas disease with heart involvement
	Postoperative cardiac complication
	Thrombosis of atrium, auricular appendage, and ventricle due to and following acute myocardial infarction
	Stable angina
	Acute heart failure
	Nonsustained ventricular tachycardia
	Cardiomyopathy due to viral infection
	Hemiplegia as late effect of cerebrovascular disease
	Tricuspid valve disorder, non-rheumatic
	Congenital stenosis of tricuspid valve
	Exacerbation of congestive heart failure
	Hypertensive heart AND chronic kidney disease with congestive heart failure
	Dextrocardia
	Cardiac arrest due to cardiac disorder
	Acute coronary syndrome
	Cardiovascular stress test abnormal
	Ostium secundum type atrial septal defect
	Common arterial trunk (truncus arteriosus)
	Left ventricular thrombus
	Pulmonary stenosis, non-rheumatic
	Mitral valve stenosis
	Chronic heart failure co-occurrent with normal ejection fraction
	Hemiplegia of dominant side as late effect of cerebrovascular disease
	Trifascicular block
	Decompensated cardiac failure
	Subsequent non-ST segment elevation myocardial infarction
	Rheumatic aortic stenosis
	Arteriosclerosis of autologous arterial coronary artery bypass graft
	Myocarditis due to infectious agent
	Cardiovascular sequelae of disorders
	Hemiparesis as late effect of cerebrovascular accident
	Left posterior fascicular block

	Aphasia as late effect of cerebrovascular accident
	Dysphasia as late effect of cerebrovascular disease
	Acute and subacute bacterial endocarditis
	Heart failure with reduced ejection fraction
	Recurrent coronary arteriosclerosis after percutaneous transluminal coronary angioplasty
	Monoplegia of nondominant upper limb as a late effect of cerebrovascular accident
	Double inlet ventricle
	Calcification of coronary artery
	Left ventricular hypertrophy
	Congenital stenosis of aortic valve
	Eisenmenger's syndrome
	Tricuspid valve disorder
	Endocarditis
	Postcardiotomy syndrome
	Left ventricular cardiac dysfunction
	Disorder of prosthetic cardiac valve
	Acute ST segment elevation myocardial infarction due to left coronary artery occlusion
	Congenital subaortic stenosis
	Hypertensive heart and renal disease with renal failure
	Benign hypertensive heart disease without congestive heart failure
	Acute on chronic heart failure co-occurrent with normal ejection fraction
	Heart valve disorder
	Ischemic heart disease
	Congenital anomaly of heart valve
	Bicuspid aortic valve
	Monoplegia of dominant upper limb as a late effect of cerebrovascular accident
	Non-rheumatic mitral valve prolapse
	Mobitz type II atrioventricular block
	Thallium stress test abnormal
	Pulmonary valve disorder
	Aneurysm of coronary vessels
	Atrial tachycardia
	Congenital stenosis of mitral valve
	Hemiplegia as late effect of cerebrovascular accident
	Monoplegia of dominant lower limb as a late effect of cerebrovascular accident
	Benign neoplasm of heart
	Prosthetic cardiac paravalvular leak
	Atresia of pulmonary valve
	Rapid atrial fibrillation
	Tricuspid valve regurgitation
	Cardiac sarcoidosis
	High output heart failure
	Atrial fibrillation and flutter
	Congenital anomaly of tricuspid valve
	Dysfunction of right cardiac ventricle
	Arteriosclerosis of coronary artery bypass graft of transplanted heart
	Mural thrombus of left ventricle
	Left ventricular systolic dysfunction
	Supraventricular premature beats
	Acute myocardial infarction of anterior wall
	Rheumatic endocarditis
	Mechanical complication of heart valve prosthesis
	Benign hypertensive heart AND renal disease
	Contusion to heart
	Injury of heart
	Hypertensive heart AND chronic kidney disease stage 5
	Junctional premature complex
	Monoplegia of nondominant lower limb as a late effect of cerebrovascular accident
	Tricuspid stenosis, non-rheumatic
	Acute rheumatic endocarditis
	Double outlet right ventricle
	Calcific coronary arteriosclerosis
	Thrombus of left atrium
	Chagas' disease with heart involvement

	Bundle branch block
	Cardiac insufficiency following cardiac surgery
	Paralytic syndrome as late effect of stroke
	Patent foramen ovale
	Left ventricular myocardial noncompaction cardiomyopathy
	Acute myocarditis
	Abnormality of fetal heart
	Abscess of aortic valve
	Arteriosclerosis of arterial coronary artery bypass graft
	Rheumatic disease of aortic valve
	Sinus bradycardia
	Premature atrial contraction
	Left bundle branch hemiblock
	Sudden cardiac death
	Decompensated chronic heart failure
	Congenital malposition of heart
	Aortic valve sclerosis
	Coronary graft stenosis
	Ostium primum defect
	Hypoplastic left heart syndrome
	Nutritional and metabolic cardiomyopathies
	Acute bacterial endocarditis
	Acquired cardiac septal defect
	Slow ventricular response
	Congenital atresia of pulmonary valve
	Acute myocardial infarction of inferior wall
	Atypical angina
	Benign hypertensive heart disease with congestive cardiac failure
	Sequela of cerebrovascular accident
	Injury of heart with hemopericardium
	Cleft leaflet of mitral valve
	Primary eosinophilic endomyocardial restrictive cardiomyopathy
	Candidal endocarditis
	Left ventricular diastolic dysfunction
	Subaortic stenosis
	Weakness as a late effect of stroke
	Tachycardia-bradycardia
	Angina co-occurrent and due to arteriosclerosis of coronary artery bypass graft
	Lipid-rich atherosclerosis of coronary artery
	Rheumatic aortic regurgitation
	Cardiac arrest during surgery
	Paroxysmal atrial flutter
	Atrial septal defect due to and following acute myocardial infarction
	Primary malignant neoplasm of heart
	Acute rejection of cardiac transplant
	Acute myocardial infarction of inferoposterior wall
	Heart transplant failure and rejection
	Ventricular flutter
	Subsequent ST segment elevation myocardial infarction
	Primary dilated cardiomyopathy
	Postpartum cardiomyopathy
	Discordant atrioventricular connection
	Hypertensive heart AND chronic kidney disease stage 3
	Congenital heart block
	Right bundle branch block AND left anterior fascicular block
	Exercise-induced angina
	Isolated (Fiedler's) myocarditis
	Endocardial fibroelastosis
	Congenital stenosis of pulmonary valve
	Malignant hypertensive heart disease without congestive heart failure
	Pre-existing hypertensive heart disease complicating pregnancy, childbirth and the puerperium
	Ventricular bigeminy
	Secondary nonischemic congestive cardiomyopathy
	Incomplete right bundle branch block

	Nonischemic congestive cardiomyopathy
	Endocarditis associated with another disorder
	Congenital insufficiency of mitral valve
	Wolff-Parkinson-White pattern
	Disorder of cardiac function
	Familial cardiomyopathy
	Hypertensive left ventricular hypertrophy
	Bilateral bundle branch block
	Left atrial enlargement
	Right cardiac ventricular dilatation
	Congenital septal defect of heart
	Atrial paroxysmal tachycardia
	Myocardial degeneration
	Hypertensive heart AND chronic kidney disease stage 4
	Cardiac complication of procedure
	Fetal heart disorder
	Monoplegia of lower limb as late effect of cerebrovascular disease
	Atrial arrhythmia
	Mitral stenosis with insufficiency
	Congenital cardiovascular disorder during pregnancy - baby not yet delivered
	Re-entry ventricular arrhythmia
	Pulmonic valve stenosis
	Post cardiac operation functional disturbance
	Viral myocarditis
	Torsades de pointes
	Malignant hypertensive heart AND renal disease
	Fetal dysrhythmia
	Rupture of chordae tendineae
	Mobitz type I incomplete atrioventricular block
	Coronary sinus abnormality
	Subsequent myocardial infarction of inferior wall
	Hypertrophic cardiomyopathy without obstruction
	Mechanical breakdown of prosthetic heart valve
	Atrial septal defect through coronary sinus orifice
	Senile cardiac amyloidosis
	Arteriosclerosis of nonautologous coronary artery bypass graft
	Ventricular arrhythmia
	Endocarditis due to systemic lupus erythematosus
	Acute myocardial infarction of inferolateral wall
	Mechanical complication due to heart valve prosthesis
	Sequelae of cardiovascular disorders
	Pre-existing hypertensive heart disease in mother complicating pregnancy
	Right ventricular hypertension
	Left ventricular outflow tract obstruction
	Wide QRS ventricular tachycardia
	Subsequent myocardial infarction of anterior wall
	Thrombus of cardiac chamber
	Carditis due to rheumatic fever
	Refractory heart failure
	Multi vessel coronary artery disease
	Congenital insufficiency of pulmonary valve
	Acute coronary artery occlusion not resulting in myocardial infarction
	Seizure disorder as sequela of stroke
	Mitral and aortic incompetence
	Subacute periendocarditis
	Angina, class I
	AV nodal re-entry tachycardia
	Congestive heart failure stage D
	Non-specific intraventricular conduction delay
	Disorder of right cardiac ventricle
	Sinus arrest
	AV-junctional (nodal) bradycardia
	Acute myocardial infarction of anterolateral wall
	AV junctional rhythm

	Post-infarction ventricular septal defect
	Benign hypertensive heart disease
	Primary hypertrophic cardiomyopathy
	Significant coronary bypass graft disease
	Nodular calcific aortic valve stenosis
	Fluency disorder as sequela of cerebrovascular disease
	Cor pulmonale
	Bacterial endocarditis
	Williams syndrome
	Vegetation of heart
	Post infarct angina
	Old inferior myocardial infarction
	Cardiac arrest as a complication of care
	Persistent sinus bradycardia
	Brugada syndrome
	Abnormality of left atrial appendage
	Myocardial ischemia
	Prinzmetal angina
	Abnormal vision as a late effect of cerebrovascular disease
	Heart-lung transplant failure and rejection
	Anomalous atrioventricular excitation
	Silent myocardial ischemia
	Primary endocardial fibroelastosis
	Visual disturbance as sequela of cerebrovascular disease
	Symptomatic sinus bradycardia
	Mitral valve vegetations
	Re-entrant atrioventricular node tachycardia
	Systolic heart failure stage B
	Cognitive deficit due to and following cerebrovascular disease
	Re-entrant atrioventricular tachycardia
	Papillary fibroelastoma of heart
	Papillary fibroelastoma
	Staphylococcal endocarditis
	Transthyretin related familial amyloid cardiomyopathy
	Pulmonic valve regurgitation
	Left ventricular aneurysm
	Myocardial bridge of coronary artery
	Rheumatic aortic stenosis with regurgitation
	Mural thrombus of left ventricle following acute myocardial infarction
	Persistent ostium secundum
	Atrial septal aneurysm
	Pre-existing hypertensive heart and chronic kidney disease in mother complicating childbirth
	Severe aortic valve stenosis
	Hemiparesis as late effect of cerebrovascular disease
	Post-phlebitic dermatosis of lower leg
	Rheumatic tricuspid valve stenosis
	Non-rheumatic pulmonary valve stenosis with regurgitation
	Acute rheumatic pericarditis
	Monoplegia of upper limb as late effect of cerebrovascular disease
	Congenital pulmonary valve abnormality
	Post-infarction mural thrombus
	Prosthetic valve endocarditis
	Cardiac volume overload
	Acute endocarditis
	Cardiac complication
	Severe sinus bradycardia
	Mitral insufficiency and aortic stenosis
	Coronary artery bypass graft occlusion
	Common ventricle
	Prosthetic cardiac valve displacement
	Idiopathic myocarditis
	Neonatal cardiac failure
	Congestive heart failure stage C
	Complete atrioventricular block as complication of atrioventricular nodal ablation

	Ectopic beats
	Sensory disorder as a late effect of cerebrovascular disease
	Hypertensive heart AND chronic kidney disease stage 2
	Ectopic atrial beats
	Spasticity as sequela of stroke
	Severe tricuspid valve regurgitation
	Non-rheumatic pulmonary valve disorder
	Myxoid transformation of mitral valve
	Neonatal tachycardia
	Rheumatic tricuspid stenosis and insufficiency
	Cardiac insufficiency during AND/OR resulting from a procedure
	Ataxia as sequela of cerebrovascular accident
	Right atrial dilatation
	Viral endocarditis
	Supraventricular arrhythmia
	Idiopathic hypertrophic subaortic stenosis
	Myxedema heart disease
	Angina decubitus
	Infundibular pulmonic stenosis
	Tachyarrhythmia
	Endocardial cushion defect
	Ventricular tachycardia with normal heart
	Eosinophilic myocarditis
	Masses on mitral apparatus
	Coronary artery fistula
	Valvular cardiomyopathy
	Acute Chagas' disease with heart involvement
	Prosthetic cardiac valve thrombosis
	Moderate aortic valve stenosis
	Acute heart failure co-occurrent with normal ejection fraction
	Atrioventricular dissociation
	Triple vessel disease of the heart
	Kyphoscoliotic heart disease
	Right hypoplastic heart syndrome
	Acute rheumatic heart disease
	Acute rheumatic myocarditis
	Coronary artery stent thrombosis
	Neonatal bradycardia
	Chronic right-sided congestive heart failure
	Ischemic congestive cardiomyopathy
	Pre-existing hypertensive heart and chronic kidney disease in mother complicating pregnancy
	Right atrial enlargement
	Mild aortic valve regurgitation
	Complete transposition of great vessels
	Arrhythmogenic right ventricular dysplasia
	Myocardial disease
	Progressive angina
	Typical angina
	Aortic valve calcification
	Congenital subaortic stenosis due to fibromuscular shelf
	Atrial hypertrophy
	Atrial thrombosis
	Cardiorenal syndrome
	Angina, class II
	Cardiac septal defects
	Injury of heart without open wound into thorax
	Heart disease in mother complicating pregnancy, childbirth AND/OR puerperium
	Right bundle branch block AND left posterior fascicular block
	Cor triatriatum
	Tachycardia-induced cardiomyopathy
	Disorder of cardiac ventricle
	Cardiac ventricular dilatation
	Cardiac disease in pregnancy
	Moderate left ventricular systolic dysfunction

	Controlled atrial fibrillation
	Prosthetic cardiac valve calcification
	Subacute endocarditis
	Moderate laceration of heart with hemopericardium
	Paralytic syndrome of nondominant side as late effect of stroke
	Holt-Oram syndrome
	Rheumatic myocarditis
	Unifocal PVCs
	Vertigo as sequela of cerebrovascular disease
	Coronary arteriosclerosis after percutaneous coronary angioplasty
	Acute right-sided congestive heart failure
	Thrombus of right atrium
	Myocardial dysfunction
	Primary idiopathic dilated cardiomyopathy
	Vertigo as late effect of stroke
	Paralytic syndrome of dominant side as late effect of stroke
	Atrial bigeminy
	Isomerism of atrial appendages
	Right ventricular diastolic dysfunction
	Severe mitral valve regurgitation
	Stokes-Adams syndrome
	Mitral valve prolapse syndrome
	Incomplete left bundle branch block
	Non-rheumatic tricuspid valve stenosis with insufficiency
	Common atrium
	Chronic bacterial endocarditis
	Severe mitral valve stenosis
	Moderate mitral valve regurgitation
	Hypertrophic cardiomegaly
	Acute myocardial infarction of inferior wall involving right ventricle
	Hyperkinetic heart disease
	Congenital cardiovascular disorders during pregnancy, childbirth and the puerperium
	Mitral and aortic stenosis
	Non-obstructive atherosclerosis of coronary artery
	Tuberculosis of heart
	Bulbus cordis and cardiac septal closure anomalies
	Prosthetic aortic valve regurgitation
	Sustained ventricular tachycardia
	Isolated diffuse granulomatous myocarditis
	New onset angina
	Cardiac arrest during AND/OR resulting from a procedure
	Hypertensive heart and renal disease with both (congestive) heart failure and renal failure
	Left main coronary artery disease
	Neurogenic bladder as late effect of cerebrovascular accident
	Atrial myxoma
	Acute ST segment elevation myocardial infarction due to occlusion of circumflex coronary artery
	Asystole
	Complete left bundle branch block
	Tricuspid valve vegetations
	D - transposition of the great vessels
	Congestive heart failure with right heart failure
	Mitral valve annular calcification
	Acute ST segment elevation myocardial infarction of inferior wall
	Acute left-sided congestive heart failure
	Myxoid transformation of cardiac valve
	Mild tricuspid valve regurgitation
	Myocarditis due to influenza virus
	Malignant hypertensive heart disease
	Atrial dilatation
	L - transposition of the great vessels
	Mixed myocardial ischemia and infarction
	Dysarthria due to and following cerebrovascular accident
	Bilateral enlargement of atria
	Ventricular tachycardia, polymorphic

	Acute myocarditis associated with another disorder
	Heart valve regurgitation
	Infection of cardiac graft
	Pre-existing hypertensive heart and renal disease complicating pregnancy, childbirth and the puerperium
	Coronary artery disease due to type 2 diabetes mellitus
	Low output heart failure
	Ventricular tachycardia, monomorphic
	Mild mitral valve regurgitation
	Syphilitic endocarditis
	Severe aortic valve regurgitation
	Right coronary artery occlusion
	Nonsustained paroxysmal ventricular tachycardia
	Laceration of heart
	Coronary arteriosclerosis following coronary artery bypass graft
	Nodal rhythm disorder
	Incomplete atrioventricular block with atrioventricular response
	Electromechanical dissociation
	Subacute bacterial endocarditis
	**Diagnostic concept name**
**Heart Failure (HF)**	
	Congestive heart failure
	Heart failure
	Chronic systolic heart failure
	Chronic diastolic heart failure
	Chronic congestive heart failure
	Hypertensive heart failure
	Hypertensive heart and renal disease with (congestive) heart failure
	Acute on chronic diastolic heart failure
	Acute on chronic systolic heart failure
	Chronic combined systolic and diastolic heart failure
	Diastolic heart failure
	Acute on chronic combined systolic and diastolic heart failure
	Systolic heart failure
	Acute systolic heart failure
	Acute diastolic heart failure
	Hypertensive heart disease without congestive heart failure
	Right ventricular failure
	Biventricular congestive heart failure
	Hypertensive heart disease with congestive heart failure
	Acute exacerbation of chronic congestive heart failure
	Left heart failure
	Acute combined systolic and diastolic heart failure
	Acute cor pulmonale
	Chronic right-sided heart failure
	Acute on chronic right-sided congestive heart failure
	Chronic cor pulmonale
	Acute congestive heart failure
	Saddle embolus of pulmonary artery with acute cor pulmonale
	Heart failure with normal ejection fraction
	Acute right-sided heart failure
	Congestive heart failure due to left ventricular systolic dysfunction
	Symptomatic congestive heart failure
	Right heart failure secondary to left heart failure
	Chronic heart failure
	Acute heart failure
	Exacerbation of congestive heart failure
	Hypertensive heart AND chronic kidney disease with congestive heart failure
	Chronic heart failure co-occurrent with normal ejection fraction
	Decompensated cardiac failure
	Heart failure with reduced ejection fraction
	Benign hypertensive heart disease without congestive heart failure
	Acute on chronic heart failure co-occurrent with normal ejection fraction

	High output heart failure
	Cardiac insufficiency following cardiac surgery
	Decompensated chronic heart failure
	Benign hypertensive heart disease with congestive cardiac failure
	Malignant hypertensive heart disease without congestive heart failure
	Refractory heart failure
	Congestive heart failure stage D
	Cor pulmonale
	Systolic heart failure stage B
	Congestive heart failure stage C
	Neonatal cardiac failure
	Cardiac insufficiency during AND/OR resulting from a procedure
	Chronic right-sided congestive heart failure
	Acute heart failure co-occurrent with normal ejection fraction
	Cardiorenal syndrome
	Acute right-sided congestive heart failure
	Hypertensive heart and renal disease with both (congestive) heart failure and renal failure
	Acute left-sided congestive heart failure
	Low output heart failure
	Congestive heart failure with right heart failure
	**Diagnostic concept name**
**ESKD**	
	End-stage renal disease<br>End stage renal failure on dialysis<br>End stage renal disease due to hypertension<br>Unscheduled or emergency dialysis treatment for an esrd patient in a hospital outpatient department …<br>Hemodialysis, maintenance in hospital<br>Anemia in end stage renal disease<br>Malignant hypertensive end stage renal disease<br>Hyperparathyroidism due to end stage renal disease on dialysis<br>Hypertensive renal disease with end stage renal failure<br>Hypertension concurrent and due to end stage renal disease on dialysis<br>End stage renal disease on dialysis due to type 2 diabetes mellitus<br>End stage renal failure with renal transplant<br>End stage renal disease due to benign hypertension<br>Hypertensive end stage renal disease<br>End stage renal disease on dialysis due to type 1 diabetes mellitus<br>Hypertension concurrent and due to end stage renal disease on dialysis due to type 2 diabetes mellit…<br>End stage renal failure untreated by renal replacement therapy<br>Hypertensive heart AND chronic kidney disease on dialysis<br>End stage renal disease on dialysis due to hypertension<br>Malignant hypertensive end stage renal disease on dialysis<br>Hemodialysis-associated amyloidosis<br>Hemodialysis, maintenance at home<br>Chronic peritoneal dialysis<br>Maintenance hemodialysis<br>End-stage renal disease<br>End stage renal failure on dialysis<br>End stage renal disease due to hypertension<br>Unscheduled or emergency dialysis treatment for an esrd patient in a hospital outpatient department …<br>Hemodialysis, maintenance in hospital<br>Anemia in end stage renal disease<br>Malignant hypertensive end stage renal disease<br>Hyperparathyroidism due to end stage renal disease on dialysis<br>Hypertensive renal disease with end stage renal failure<br>Hypertension concurrent and due to end stage renal disease on dialysis<br>End stage renal disease on dialysis due to type 2 diabetes mellitus<br>End stage renal failure with renal transplant<br>End stage renal disease due to benign hypertension<br>Hypertensive end stage renal disease<br>End stage renal disease on dialysis due to type 1 diabetes mellitus<br>Hypertension concurrent and due to end stage renal disease on dialysis due to type 2 diabetes mellit…<br>End stage renal failure untreated by renal replacement therapy<br>Hypertensive heart AND chronic kidney disease on dialysis<br>End stage renal disease on dialysis due to hypertension

	Malignant hypertensive end stage renal disease on dialysis<br>Hemodialysis-associated amyloidosis<br>Hemodialysis, maintenance at home<br>Chronic peritoneal dialysis<br>Maintenance hemodialysis

Supplemental Table 1. Descriptive Statistics of Patients with and without Baseline CKD/ESKD

Characteristic[1]	Overall, N = 2,385,203	No Baseline CKD/ESKD, N = 2,031,514	Baseline CKD/ESKD, N = 353,689	p value[2]
Age at COVID-19	51 (35, 66)	47 (33, 62)	70 (60, 79)	<0.001
Age Group				<0.001
18-29	369,703 (15%)	365,824 (18%)	3,879 (1.1%)	
30-39	387,149 (16%)	377,433 (19%)	9,716 (2.7%)	
40-49	367,595 (15%)	346,747 (17%)	20,848 (5.9%)	
50-59	404,086 (17%)	356,399 (18%)	47,687 (13%)	
60-69	404,537 (17%)	319,056 (16%)	85,481 (24%)	
70-79	293,856 (12%)	191,540 (9.4%)	102,316 (29%)	
80+	158,277 (6.6%)	74,515 (3.7%)	83,762 (24%)	
Sex				<0.001
Female	1,477,627 (62%)	1,278,720 (63%)	198,907 (56%)	
Male	907,576 (38%)	752,794 (37%)	154,782 (44%)	
Race/Ethnicity				<0.001
Black or African American Non-Hispanic	288,087 (12%)	231,450 (11%)	56,637 (16%)	
Hispanic or Latino Any Race	230,673 (9.7%)	209,521 (10%)	21,152 (6.0%)	
Other non-Hispanic	140,558 (5.9%)	122,870 (6.0%)	17,688 (5.0%)	
Unknown	100,008 (4.2%)	91,363 (4.5%)	8,645 (2.4%)	
White non-Hispanic	1,625,877 (68%)	1,376,310 (68%)	249,567 (71%)	
Rurality				<0.001
Urban	1,769,318 (74%)	1,515,528 (75%)	253,790 (72%)	
Rural	369,036 (15%)	308,123 (15%)	60,913 (17%)	
Missing	246,849 (10%)	207,863 (10%)	38,986 (11%)	
COVID-19 Vaccination Status				<0.001
No Documented COVID-19 Vaccination	1,448,626 (61%)	1,260,351 (62%)	188,275 (53%)	
Primary COVID-19 Vaccination Documented	460,389 (19%)	395,772 (19%)	64,617 (18%)	
Primary and Additional COVID-19 Dose(s) Documented	476,188 (20%)	375,391 (18%)	100,797 (28%)	
SARS-CoV-2 Variant-Dominant Period				<0.001
Delta (B.1.617.2)	296,455 (12%)	263,845 (13%)	32,610 (9.2%)	
Omicron (B.1.1.529, BA.2, BA.2.12.1)	1,142,650 (48%)	1,001,360 (49%)	141,290 (40%)	
Omicron (BA.5, BQ.1.1, XBB.1.5)	946,098 (40%)	766,309 (38%)	179,789 (51%)	
Heart Failure Before COVID-19	154,850 (6.5%)	62,368 (3.1%)	92,482 (26%)	<0.001
Cardiovascular Disease Before COVID-19	552,225 (23%)	343,495 (17%)	208,730 (59%)	<0.001
Hypertension Before COVID-19	855,635 (36%)	574,714 (28%)	280,921 (79%)	<0.001
Obesity Before COVID-19	1,027,886 (43%)	828,117 (41%)	199,769 (56%)	<0.001
Tobacco Usage Before COVID-19	325,854 (14%)	266,618 (13%)	59,236 (17%)	<0.001
Diabetes Before COVID-19	390,377 (16%)	244,611 (12%)	145,766 (41%)	<0.001
Baseline eGFR Before COVID-19	89 (72, 104)	96 (83, 108)	57 (45, 70)	<0.001
No eGFR Available in the Year Before COVID-19	*1,204,367*	*1,137,873*	*66,494*	
Adverse post-COVID-19 Events				
Long COVID	30,987 (1.3%)	24,591 (1.2%)	6,396 (1.8%)	<0.001
Death After COVID-19 (with no Long COVID diagnosis)	39,983 (1.7%)	18,246 (0.9%)	21,737 (6.1%)	<0.001
Any Death After COVID-19	40,882 (1.7%)	18,649 (0.9%)	22,233 (6.3%)	<0.001

1. n (%); Median (IQR)
2. Pearson's Chi-squared test; Wilcoxon rank sum test

Supplemental Table 1 provides descriptive statistics for patients with a documented SARS-CoV-2 infection between October 1, 2021, and September 30, 2023. This includes all patients seen at clinics with >=250 long COVID cases based on study inclusion/exclusion criteria. Abbreviations: Coronavirus disease 2019 (COVID-19), acute respiratory syndrome coronavirus (SARS-CoV-2), chronic kidney disease (CKD), end-stage kidney disease (ESKD), acute kidney injury (AKI).

Supplemental Table 2. Univariate and Multivariable Cox Proportional Hazards Regression for Long COVID

Characteristic	N Events / N (%)	Unadjusted HR (95% CI)	p value	Adjusted sHR (95% CI)	p value
CKD/ESKD Status					
No baseline CKD/ESKD	24,591 / 2,031,514 (1.2%)	Reference		Reference	
Baseline CKD/ESKD	6,396 / 353,689 (1.8%)	1.51 (1.47, 1.55)	<0.001	1.16 (1.12, 1.20)	<0.001
Sex					
Female	20,740 / 1,477,627 (1.4%)	Reference		Reference	
Male	10,247 / 907,576 (1.1%)	0.80 (0.78, 0.82)	<0.001	0.73 (0.71, 0.74)	<0.001
Age Group					
60-69	6,588 / 404,537 (1.6%)	Reference		Reference	
18-29	2,077 / 369,703 (0.56%)	0.34 (0.32, 0.36)	<0.001	0.40 (0.38, 0.42)	<0.001
30-39	3,878 / 387,149 (1.0%)	0.61 (0.59, 0.64)	<0.001	0.68 (0.65, 0.71)	<0.001
40-49	5,371 / 367,595 (1.5%)	0.89 (0.86, 0.93)	<0.001	0.96 (0.92, 1.00)	0.03
50-59	6,509 / 404,086 (1.6%)	0.99 (0.95, 1.02)	0.46	1.02 (0.99, 1.06)	0.25
70-79	4,505 / 293,856 (1.5%)	0.94 (0.91, 0.98)	0.002	0.90 (0.87, 0.94)	<0.001
80+	2,059 / 158,277 (1.3%)	0.80 (0.76, 0.84)	<0.001	0.74 (0.70, 0.77)	<0.001
Race/Ethnicity					
White non-Hispanic	22,075 / 1,625,877 (1.4%)	Reference		Reference	
Black or African American Non-Hispanic	3,252 / 288,087 (1.1%)	0.83 (0.80, 0.86)	<0.001	0.80 (0.77, 0.83)	<0.001
Hispanic or Latino Any Race	2,818 / 230,673 (1.2%)	0.91 (0.87, 0.94)	<0.001	1.01 (0.97, 1.05)	0.77
Other non-Hispanic	1,611 / 140,558 (1.1%)	0.85 (0.81, 0.89)	<0.001	0.98 (0.93, 1.03)	0.34
Unknown	1,231 / 100,008 (1.2%)	0.92 (0.87, 0.98)	0.005	1.07 (1.01, 1.14)	0.02
SARS-CoV-2 Variant Period					
Omicron (B.1.1.529, BA.2, BA.2.12.1)	14,575 / 1,142,650 (1.3%)	Reference		Reference	
Delta (B.1.617.2)	5,732 / 296,455 (1.9%)	1.52 (1.48, 1.57)	<0.001	1.53 (1.48, 1.58)	<0.001
Omicron (BA.5, BQ.1.1, XBB.1.5)	10,680 / 946,098 (1.1%)	0.90 (0.88, 0.92)	<0.001	0.80 (0.78, 0.82)	<0.001
COVID-19 Vaccination Status					
No Documented COVID-19 Vaccination	20,053 / 1,448,626 (1.4%)	Reference		Reference	
Primary COVID-19 Vaccination Documented	5,235 / 460,389 (1.1%)	0.82 (0.79, 0.84)	<0.001	0.77 (0.75, 0.80)	<0.001
Primary and Additional COVID-19 Dose(s) Documented	5,699 / 476,188 (1.2%)	0.86 (0.84, 0.89)	<0.001	0.85 (0.82, 0.87)	<0.001
Heart Failure					
No History of HF	27,890 / 2,230,353 (1.3%)	Reference		Reference	
History of HF	3,097 / 154,850 (2.0%)	1.62 (1.56, 1.68)	<0.001	1.02 (0.97, 1.06)	0.45
Diabetes					
No History of Diabetes	24,439 / 1,994,826 (1.2%)	Reference		Reference	
History of Diabetes	6,548 / 390,377 (1.7%)	1.38 (1.34, 1.42)	<0.001	0.98 (0.95, 1.01)	0.11
Hypertension					
No History of HTN	16,545 / 1,529,568 (1.1%)	Reference		Reference	
History of HTN	14,442 / 855,635 (1.7%)	1.57 (1.54, 1.61)	<0.001	1.13 (1.09, 1.16)	<0.001
Cardiovascular Disease					
No History of CVD	20,609 / 1,832,978 (1.1%)	Reference		Reference	
History of CVD	10,378 / 552,225 (1.9%)	1.69 (1.65, 1.73)	<0.001	1.42 (1.38, 1.47)	<0.001
Obesity					
No History of Obesity	14,340 / 1,357,317 (1.1%)	Reference		Reference	
History of Obesity	16,647 / 1,027,886 (1.6%)	1.54 (1.51, 1.57)	<0.001	1.35 (1.32, 1.38)	<0.001
Tobacco Usage					
No History of Tobacco Usage	26,101 / 2,059,349 (1.3%)	Reference		Reference	
History of Tobacco Usage	4,886 / 325,854 (1.5%)	1.19 (1.15, 1.22)	<0.001	1.07 (1.04, 1.10)	<0.001
Rurality					
Urban	22,721 / 1,769,318 (1.3%)	Reference		Reference	
Rural	4,854 / 369,036 (1.3%)	1.02 (0.98, 1.05)	0.34	0.94 (0.91, 0.97)	<0.001
Missing	3,412 / 246,849 (1.4%)	1.09 (1.05, 1.12)	<0.001	1.03 (1.00, 1.07)	0.09

Supplemental Table 2 includes univariate and multivariable Cox Proportional Hazards for long COVID (U09.9) among patients with a SARS-CoV-2 infection documented between October 1, 2021, and September 30, 2023. Abbreviations: Number of Events (N Events), Number of Patients (N), hazard ratio (HR), subdistribution hazard ratio (sHR), Coronavirus disease 2019 (COVID-19), acute respiratory syndrome coronavirus (SARS-CoV-2), chronic kidney disease (CKD), end-stage kidney disease (ESKD), acute kidney injury (AKI).

Supplemental Table 3. Descriptive Statistics of Patients with and without Baseline Mild CKD (Stage 3a)

Characteristic[1]	Overall, N = 2,096,079	No baseline CKD/ESKD, N = 2,020,945	Baseline CKD3a N = 75,134	p value[2]
Age at COVID-19	48 (34, 63)	47 (33, 62)	73 (65, 81)	<0.001
Age Group				<0.001
18-29	365,800 (17%)	365,632 (18%)	168 (0.2%)	
30-39	377,676 (18%)	377,017 (19%)	659 (0.9%)	
40-49	348,287 (17%)	345,979 (17%)	2,308 (3.1%)	
50-59	362,466 (17%)	354,859 (18%)	7,607 (10%)	
60-69	334,482 (16%)	316,609 (16%)	17,873 (24%)	
70-79	213,817 (10%)	188,836 (9.3%)	24,981 (33%)	
80+	93,551 (4.5%)	72,013 (3.6%)	21,538 (29%)	
Sex				<0.001
Female	1,315,687 (63%)	1,272,851 (63%)	42,836 (57%)	
Male	780,392 (37%)	748,094 (37%)	32,298 (43%)	
Race/Ethnicity				<0.001
Black or African American Non-Hispanic	240,592 (11%)	229,676 (11%)	10,916 (15%)	
Hispanic or Latino Any Race	211,963 (10%)	208,842 (10%)	3,121 (4.2%)	
Other non-Hispanic	125,160 (6.0%)	122,314 (6.1%)	2,846 (3.8%)	
Unknown	92,713 (4.4%)	91,050 (4.5%)	1,663 (2.2%)	
White non-Hispanic	1,425,651 (68%)	1,369,063 (68%)	56,588 (75%)	
Rurality				<0.001
Urban	1,562,133 (75%)	1,508,497 (75%)	53,636 (71%)	
Rural	319,687 (15%)	305,887 (15%)	13,800 (18%)	
Missing	214,259 (10%)	206,561 (10%)	7,698 (10%)	
COVID-19 Vaccination Status				<0.001
No Documented COVID-19 Vaccination	1,289,575 (62%)	1,252,979 (62%)	36,596 (49%)	
Primary COVID-19 Vaccination Documented	408,429 (19%)	394,268 (20%)	14,161 (19%)	
Primary and Additional COVID-19 Dose(s) Documented	398,075 (19%)	373,698 (18%)	24,377 (32%)	
SARS-CoV-2 Variant-Dominant Period				<0.001
Delta (B.1.617.2)	269,153 (13%)	262,558 (13%)	6,595 (8.8%)	
Omicron (B.1.1.529, BA.2, BA.2.12.1)	1,025,976 (49%)	996,972 (49%)	29,004 (39%)	
Omicron (BA.5, BQ.1.1, XBB.1.5)	800,950 (38%)	761,415 (38%)	39,535 (53%)	
Heart Failure Before COVID-19	79,623 (3.8%)	60,954 (3.0%)	18,669 (25%)	<0.001
Cardiovascular Disease Before COVID-19	384,285 (18%)	339,785 (17%)	44,500 (59%)	<0.001
Hypertension Before COVID-19	630,162 (30%)	569,703 (28%)	60,459 (80%)	<0.001
Obesity Before COVID-19	866,337 (41%)	824,061 (41%)	42,276 (56%)	<0.001
Tobacco Usage Before COVID-19	276,135 (13%)	265,007 (13%)	11,128 (15%)	<0.001
Diabetes Before COVID-19	271,009 (13%)	241,890 (12%)	29,119 (39%)	<0.001
Baseline eGFR Before COVID-19	94 (80, 107)	96 (83, 108)	54 (48, 61)	<0.001
No eGFR Available in the Year Before COVID-19	*1,139,419*	*1,130,376*	*9,043*	
Adverse post-COVID-19 Events				
Long COVID	25,753 (1.2%)	24,342 (1.2%)	1,411 (1.9%)	<0.001
Death After COVID-19 (with no Long COVID diagnosis)	20,067 (1.0%)	16,622 (0.8%)	3,445 (4.6%)	<0.001
Any Death After COVID-19	20,515 (1.0%)	16,986 (0.8%)	3,529 (4.7%)	<0.001

1. n (%); Median (IQR)
2. Pearson's Chi-squared test; Wilcoxon rank sum test

Supplemental Table 3 provides descriptive statistics for patients with a documented SARS-CoV-2 infection between October 1, 2021, and September 30, 2023. This includes all patients seen at clinics with >=250 long COVID cases based on study inclusion/exclusion criteria. Abbreviations: Coronavirus disease 2019 (COVID-19), acute respiratory syndrome coronavirus (SARS-CoV-2), chronic kidney disease (CKD), end-stage kidney disease (ESKD), acute kidney injury (AKI), CKD Stage 3a (eGFR 45 -59 ml/min/1.73m2).

Supplemental Table 4. Descriptive Statistics of Patients with and without baseline mild CKD (Stage 3a) after PSM

Characteristic[1]	Overall, N = 150,264	No baseline CKD/ESKD, N = 75,132	Baseline CKD3a N = 75,132	SMD (95% CI)[2]
Age at COVID-19	73 (65, 81)	73 (65, 80)	73 (65, 81)	-0.03 (-0.07, 0.01)
Age Group				0.03 (-0.01, 0.07)
18-29	339 (0.2%)	171 (0.2%)	168 (0.2%)	
30-39	1,324 (0.9%)	665 (0.9%)	659 (0.9%)	
40-49	4,637 (3.1%)	2,329 (3.1%)	2,308 (3.1%)	
50-59	15,248 (10%)	7,641 (10%)	7,607 (10%)	
60-69	35,904 (24%)	18,033 (24%)	17,871 (24%)	
70-79	50,183 (33%)	25,202 (34%)	24,981 (33%)	
80+	42,629 (28%)	21,091 (28%)	21,538 (29%)	
Sex				0.00 (-0.04, 0.04)
Female	85,672 (57%)	42,836 (57%)	42,836 (57%)	
Male	64,592 (43%)	32,296 (43%)	32,296 (43%)	
Race/Ethnicity				0.00 (-0.04, 0.04)
Black or African American Non-Hispanic	21,832 (15%)	10,916 (15%)	10,916 (15%)	
Hispanic or Latino Any Race	6,242 (4.2%)	3,121 (4.2%)	3,121 (4.2%)	
Other non-Hispanic	5,688 (3.8%)	2,844 (3.8%)	2,844 (3.8%)	
Unknown	3,326 (2.2%)	1,663 (2.2%)	1,663 (2.2%)	
White non-Hispanic	113,176 (75%)	56,588 (75%)	56,588 (75%)	
Rurality				0.03 (-0.01, 0.07)
Urban	107,863 (72%)	54,229 (72%)	53,634 (71%)	
Rural	26,917 (18%)	13,117 (17%)	13,800 (18%)	
Missing	15,484 (10%)	7,786 (10%)	7,698 (10%)	
COVID-19 Vaccination Status				0.06 (0.02, 0.10)
No Documented COVID-19 Vaccination	76,695 (51%)	40,100 (53%)	36,595 (49%)	
Primary COVID-19 Vaccination Documented	26,963 (18%)	12,803 (17%)	14,160 (19%)	
Primary and Additional COVID-19 Dose(s) Documented	46,606 (31%)	22,229 (30%)	24,377 (32%)	
SARS-CoV-2 Variant-Dominant Period				0.00 (-0.04, 0.04)
Delta (B.1.617.2)	13,186 (8.8%)	6,593 (8.8%)	6,593 (8.8%)	
Omicron (B.1.1.529, BA.2, BA.2.12.1)	58,008 (39%)	29,004 (39%)	29,004 (39%)	
Omicron (BA.5, BQ.1.1, XBB.1.5)	79,070 (53%)	39,535 (53%)	39,535 (53%)	
Heart Failure Before COVID-19	25,524 (17%)	6,855 (9.1%)	18,669 (25%)	-0.02 (-0.06, 0.02)
Cardiovascular Disease Before COVID-19	72,885 (49%)	28,387 (38%)	44,498 (59%)	-0.08 (-0.12, -0.05)
Hypertension Before COVID-19	101,343 (67%)	40,886 (54%)	60,457 (80%)	-0.06 (-0.10, -0.02)
Obesity Before COVID-19	71,241 (47%)	28,966 (39%)	42,275 (56%)	-0.06 (-0.10, -0.02)
Tobacco Usage Before COVID-19	19,833 (13%)	8,705 (12%)	11,128 (15%)	0.02 (-0.02, 0.06)
Diabetes Before COVID-19	43,809 (29%)	14,691 (20%)	29,118 (39%)	0.04 (0.00, 0.08)
Baseline eGFR Before COVID-19	63 (52, 81)	84 (74, 92)	54 (48, 61)	0.07 (0.03, 0.11)
No eGFR Available in the Year Before COVID-19	*40,811*	*31,769*	*9,042*	
Adverse post-COVID-19 Events				
Long COVID	2,474 (1.6%)	1,063 (1.4%)	1,411 (1.9%)	-0.05 (-0.09, -0.01)
Death After COVID-19 (with no Long COVID diagnosis)	5,932 (3.9%)	2,487 (3.3%)	3,445 (4.6%)	-0.02 (-0.06, 0.02)
Any Death After COVID-19	73 (65, 81)	73 (65, 80)	73 (65, 81)	-0.02 (-0.06, 0.02)

1. n (%); Median (IQR)
2. Standardized mean differences (SMD) with 95% confidence intervals (CI)

Supplemental Table 4 provides descriptive statistics for patients with a documented SARS-CoV-2 infection between October 1, 2021, and September 30, 2023, after propensity-score matching (PSM). Patients were PSM using logistic regression with 1:1 matching for binary long COVID status with exact matching on sex, race/ethnicity, COVID-19 variant period, and data-contributing site and nearest neighbor PSM on age. Abbreviations: Coronavirus disease 2019 (COVID-19), acute respiratory syndrome coronavirus (SARS-CoV-2), chronic kidney disease (CKD), end-stage kidney disease (ESKD), acute kidney injury (AKI), CKD Stage 3a (eGFR 45 -59 ml/min/1.73m2).

Supplemental Table 5. Univariate and Multivariable Cox Proportional Hazards Regression for Long COVID (mild CKD - Stage 3a)

Characteristic	Unadjusted HR (95% CI)	p value	Adjusted sHR (95% CI)	p value
CKD/ESKD Status				
Non-CKD/ESKD	Reference		Reference	
CKD Stage 3a	1.57 (1.49, 1.65)	<0.001	1.21 (1.15, 1.29)	<0.001
Sex				
Female	Reference		Reference	
Male	0.79 (0.76, 0.81)	<0.001	0.71 (0.70, 0.73)	<0.001
Age Group				
60-69	Reference		Reference	
18-29	0.36 (0.34, 0.38)	<0.001	0.40 (0.38, 0.42)	<0.001
30-39	0.64 (0.61, 0.67)	<0.001	0.68 (0.65, 0.71)	<0.001
40-49	0.93 (0.89, 0.96)	<0.001	0.97 (0.93, 1.01)	0.12
50-59	1.01 (0.98, 1.05)	0.49	1.03 (0.99, 1.07)	0.10
70-79	0.91 (0.87, 0.96)	<0.001	0.88 (0.84, 0.92)	<0.001
80+	0.83 (0.78, 0.88)	<0.001	0.77 (0.72, 0.82)	<0.001
Race/Ethnicity				
White non-Hispanic	Reference		Reference	
Black or African American Non-Hispanic	0.79 (0.76, 0.83)	<0.001	0.78 (0.75, 0.82)	<0.001
Hispanic or Latino Any Race	0.92 (0.88, 0.96)	<0.001	1.01 (0.97, 1.06)	0.62
Other non-Hispanic	0.88 (0.83, 0.93)	<0.001	1.01 (0.95, 1.06)	0.79
Unknown	0.95 (0.89, 1.01)	0.095	1.09 (1.03, 1.16)	0.005
SARS-CoV-2 Variant Period				
Omicron (B.1.1.529, BA.2, BA.2.12.1)	Reference		Reference	
Delta (B.1.617.2)	1.53 (1.48, 1.59)	<0.001	1.53 (1.48, 1.59)	<0.001
Omicron (BA.5, BQ.1.1, XBB.1.5)	0.93 (0.91, 0.96)	<0.001	0.83 (0.81, 0.85)	<0.001
COVID-19 Vaccination Status				
No Documented COVID-19 Vaccination	Reference		Reference	
Primary COVID-19 Vaccination Documented	0.80 (0.77, 0.82)	<0.001	0.76 (0.74, 0.79)	<0.001
Primary and Additional COVID-19 Dose(s) Documented	0.85 (0.82, 0.87)	<0.001	0.83 (0.80, 0.86)	<0.001
Heart Failure				
No History of HF	Reference		Reference	
History of HF	1.67 (1.59, 1.76)	<0.001	1.04 (0.98, 1.10)	0.22
Diabetes				
No History of Diabetes	Reference		Reference	
History of Diabetes	1.34 (1.30, 1.39)	<0.001	0.98 (0.95, 1.02)	0.27
Hypertension				
No History of HTN	Reference		Reference	
History of HTN	1.52 (1.49, 1.56)	<0.001	1.12 (1.08, 1.15)	<0.001
Cardiovascular Disease				
No History of CVD	Reference		Reference	
History of CVD	1.69 (1.64, 1.74)	<0.001	1.45 (1.40, 1.50)	<0.001
Obesity				
No History of Obesity	Reference		Reference	
History of Obesity	1.50 (1.46, 1.54)	<0.001	1.34 (1.31, 1.38)	<0.001
Tobacco Usage				
No History of Tobacco Usage	Reference		Reference	
History of Tobacco Usage	1.17 (1.13, 1.21)	<0.001	1.06 (1.02, 1.10)	<0.001
Rurality				
Urban	Reference		Reference	
Rural	0.99 (0.96, 1.03)	0.58	0.91 (0.88, 0.95)	<0.001
Missing	1.10 (1.05, 1.14)	<0.001	1.04 (1.00, 1.09)	0.04

Supplemental Table 5 includes univariate and multivariable Cox Proportional Hazards for long COVID (U09.9) among patients with a SARS-CoV-2 infection documented between October 1, 2021, and September 30, 2023. Abbreviations: hazard ratio (HR), subdistribution hazard ratio (sHR), Coronavirus disease 2019 (COVID-19), acute respiratory syndrome coronavirus (SARS-CoV-2), chronic kidney disease (CKD), end-stage kidney disease (ESKD), acute kidney injury (AKI).

Supplemental Table 6. Univariate and Multivariable Cox Proportional Hazards Regression for Long COVID after PSM (mild CKD - Stage 3a)

Characteristic	Unadjusted HR (95% CI)	p value	Adjusted sHR (95% CI)	p value
CKD/ESKD Status				
Non-CKD/ESKD	Reference		Reference	
CKD Stage 3a	1.33 (1.23, 1.44)	<0.001	1.15 (1.05, 1.25)	0.002
Sex				
Female	Reference		Reference	
Male	0.81 (0.75, 0.88)	<0.001	0.79 (0.72, 0.85)	<0.001
Age Group				
60-69	Reference		Reference	
18-29	1.11 (0.53, 2.33)	0.79	1.08 (0.51, 2.29)	0.83
30-39	1.17 (0.81, 1.70)	0.40	1.17 (0.81, 1.71)	0.40
40-49	0.98 (0.78, 1.23)	0.84	0.96 (0.76, 1.20)	0.72
50-59	0.97 (0.84, 1.11)	0.64	0.95 (0.82, 1.09)	0.53
70-79	0.87 (0.78, 0.96)	0.005	0.90 (0.81, 1.00)	0.05
80+	0.74 (0.67, 0.83)	<0.001	0.80 (0.71, 0.90)	<0.001
Race/Ethnicity				
Black or African American Non-Hispanic	0.95 (0.84, 1.06)	0.34	0.86 (0.76, 0.97)	0.01
Hispanic or Latino Any Race	1.05 (0.86, 1.28)	0.62	1.00 (0.82, 1.21)	0.98
Other non-Hispanic	1.03 (0.84, 1.26)	0.80	1.07 (0.87, 1.32)	0.50
Unknown	1.00 (0.77, 1.31)	0.98	1.06 (0.81, 1.39)	0.68
White non-Hispanic	Reference		Reference	
SARS-CoV-2 Variant Period				
Omicron (B.1.1.529, BA.2, BA.2.12.1)	Reference		Reference	
Delta (B.1.617.2)	1.58 (1.41, 1.77)	<0.001	1.51 (1.34, 1.70)	<0.001
Omicron (BA.5, BQ.1.1, XBB.1.5)	0.64 (0.59, 0.70)	<0.001	0.66 (0.61, 0.72)	<0.001
COVID-19 Vaccination Status				
No Documented COVID-19 Vaccination	Reference		Reference	
Primary COVID-19 Vaccination Documented	0.92 (0.83, 1.02)	0.11	0.83 (0.74, 0.92)	<0.001
Primary and Additional COVID-19 Dose(s) Documented	0.65 (0.59, 0.72)	<0.001	0.72 (0.65, 0.80)	<0.001
Heart Failure				
No History of HF	Reference		Reference	
History of HF	1.41 (1.29, 1.55)	<0.001	1.17 (1.05, 1.31)	0.005
Diabetes				
No History of Diabetes	Reference		Reference	
History of Diabetes	1.19 (1.09, 1.29)	<0.001	0.96 (0.88, 1.06)	0.43
Hypertension				
No History of HTN	Reference		Reference	
History of HTN	1.39 (1.27, 1.52)	<0.001	1.22 (1.09, 1.35)	<0.001
Cardiovascular Disease				
No History of CVD	Reference		Reference	
History of CVD	1.33 (1.23, 1.44)	<0.001	1.21 (1.09, 1.33)	<0.001
Obesity				
No History of Obesity	Reference		Reference	
History of Obesity	1.49 (1.37, 1.61)	<0.001	1.31 (1.20, 1.43)	<0.001
Tobacco Usage				
No History of Tobacco Usage	Reference		Reference	
History of Tobacco Usage	1.19 (1.06, 1.32)	0.002	1.07 (0.96, 1.20)	0.24
Rurality				
Urban	Reference		Reference	
Rural	1.08 (0.97, 1.19)	0.14	1.03 (0.93, 1.14)	0.58
Missing	0.94 (0.82, 1.08)	0.37	0.87 (0.76, 1.00)	0.06

Supplemental Table 6 includes univariate and multivariable Cox Proportional Hazards for long COVID (U09.9) among patients with a SARS-CoV-2 infection documented between October 1, 2021, and September 30, 2023, after propensity-score matching (PSM). Patients were PSM using logistic regression with 1:1 matching for binary long COVID status with exact matching on sex, race/ethnicity, COVID-19 variant period, and data-contributing site and nearest neighbor PSM on age. Abbreviations: hazard ratio (HR), subdistribution hazard ratio (sHR), Coronavirus disease 2019 (COVID-19), acute respiratory syndrome coronavirus (SARS-CoV-2), chronic kidney disease (CKD), end-stage kidney disease (ESKD), acute kidney injury (AKI)

Supplemental Table 7. Descriptive Statistics of Patients with and without Baseline Long COVID

Characteristic[1]	Overall, N = 2,020,162	Acute COVID-19, N = 1,995,902	Long COVID, N = 24,260	*p* value
Age at COVID-19	47 (33, 62)	47 (33, 62)	52 (40, 63)	<0.001
Age Group				<0.001
18-29	365,681 (18%)	363,646 (18%)	2,035 (8.4%)	
30-39	377,035 (19%)	373,323 (19%)	3,712 (15%)	
40-49	345,962 (17%)	341,049 (17%)	4,913 (20%)	
50-59	354,726 (18%)	349,221 (17%)	5,505 (23%)	
60-69	316,195 (16%)	311,474 (16%)	4,721 (19%)	
70-79	188,467 (9.3%)	185,919 (9.3%)	2,548 (11%)	
80+	72,096 (3.6%)	71,270 (3.6%)	826 (3.4%)	
Sex				<0.001
Female	1,272,845 (63%)	1,256,220 (63%)	16,625 (69%)	
Male	747,317 (37%)	739,682 (37%)	7,635 (31%)	
Race/Ethnicity				<0.001
Black or African American Non-Hispanic	229,773 (11%)	227,526 (11%)	2,247 (9.3%)	
Hispanic or Latino Any Race	208,877 (10%)	206,456 (10%)	2,421 (10.0%)	
Other non-Hispanic	122,343 (6.1%)	120,997 (6.1%)	1,346 (5.5%)	
Unknown	90,976 (4.5%)	89,909 (4.5%)	1,067 (4.4%)	
White non-Hispanic	1,368,193 (68%)	1,351,014 (68%)	17,179 (71%)	
Rurality				<0.001
Urban	1,507,908 (75%)	1,489,934 (75%)	17,974 (74%)	
Rural	305,903 (15%)	302,321 (15%)	3,582 (15%)	
Missing	*206,351 (10%)*	*203,647 (10%)*	*2,704 (11%)*	
COVID-19 Vaccination Status				<0.001
No Documented COVID-19 Vaccination	1,252,584 (62%)	1,236,443 (62%)	16,141 (67%)	
Primary COVID-19 Vaccination Documented	394,171 (20%)	390,150 (20%)	4,021 (17%)	
Primary and Additional COVID-19 Dose(s) Documented	373,407 (18%)	369,309 (19%)	4,098 (17%)	
SARS-CoV-2 Variant-Dominant Period				<0.001
Delta (B.1.617.2)	262,362 (13%)	257,712 (13%)	4,650 (19%)	
Omicron (B.1.1.529, BA.2, BA.2.12.1)	996,967 (49%)	985,489 (49%)	11,478 (47%)	
Omicron (BA.5, BQ.1.1, XBB.1.5)	760,833 (38%)	752,701 (38%)	8,132 (34%)	
Heart Failure Before COVID-19	60,791 (3.0%)	59,655 (3.0%)	1,136 (4.7%)	<0.001
Cardiovascular Disease Before COVID-19	339,041 (17%)	332,970 (17%)	6,071 (25%)	<0.001
Hypertension Before COVID-19	568,770 (28%)	559,846 (28%)	8,924 (37%)	<0.001
Obesity Before COVID-19	823,258 (41%)	811,004 (41%)	12,254 (51%)	<0.001
Tobacco Usage Before COVID-19	264,842 (13%)	261,218 (13%)	3,624 (15%)	<0.001
Diabetes Before COVID-19	241,626 (12%)	237,954 (12%)	3,672 (15%)	<0.001
Baseline eGFR Before COVID-19	96 (83, 108)	96 (83, 108)	94 (82, 105)	<0.001
No eGFR Available in the Year Before COVID-19	*1,131,347*	*1,120,291*	*11,056*	
Adverse post-COVID-19 Events				
Incident CKD/ESKD	53,667 (2.7%)	52,398 (2.6%)	1,269 (5.2%)	<0.001
Death After COVID-19 (with no Long COVID diagnosis)	15,167 (0.8%)	14,868 (0.7%)	299 (1.2%)	<0.001
Any Death After COVID-19	17,699 (0.9%)	17,322 (0.9%)	377 (1.6%)	<0.001

1. n (%); Median (IQR)
2. Pearson's Chi-squared test; Wilcoxon rank sum test

Supplemental Table 7 provides descriptive statistics for patients with a documented SARS-CoV-2 infection between October 1, 2021, and September 30, 2023. This includes all patients seen at clinics with >=250 long COVID cases based on study inclusion/exclusion criteria. Patients with a history of CKD/ESKD are not included in this analysis. Abbreviations: Coronavirus disease 2019 (COVID-19), acute respiratory syndrome coronavirus (SARS-CoV-2), chronic kidney disease (CKD), end-stage kidney disease (ESKD), acute kidney injury (AKI)

Supplemental Table 8. Univariate and Multivariable Cox Proportional Hazards Regression for incident CKD/ESKD

Characteristic	N Events / N (%)	Unadjusted HR (95% CI)	p value	Adjusted sHR (95% CI)	p value
COVID-19 Status					
Acute COVID-19	52,398 / 1,995,902 (2.6%)	Reference		Reference	
Long COVID	1,269 / 24,260 (5.2%)	2.02 (1.91, 2.13)	<0.001	1.66 (1.57, 1.76)	<0.001
Sex					
Female	30,771 / 1,272,845 (2.4%)	Reference		Reference	
Male	22,896 / 747,317 (3.1%)	1.27 (1.25, 1.29)	<0.001	1.01 (0.99, 1.03)	0.20
Age Group					
60-69	14,428 / 316,195 (4.6%)	Reference		Reference	
18-29	903 / 365,681 (0.25%)	0.05 (0.05, 0.06)	<0.001	0.07 (0.07, 0.07)	<0.001
30-39	2,103 / 377,035 (0.56%)	0.12 (0.11, 0.12)	<0.001	0.15 (0.14, 0.16)	<0.001
40-49	4,549 / 345,962 (1.3%)	0.28 (0.27, 0.29)	<0.001	0.33 (0.31, 0.34)	<0.001
50-59	9,503 / 354,726 (2.7%)	0.58 (0.56, 0.59)	<0.001	0.61 (0.60, 0.63)	<0.001
70-79	13,682 / 188,467 (7.3%)	1.62 (1.58, 1.66)	<0.001	1.56 (1.52, 1.60)	<0.001
80+	8,499 / 72,096 (12%)	2.71 (2.64, 2.79)	<0.001	2.54 (2.47, 2.62)	<0.001
Race/Ethnicity					
Black or African American Non-Hispanic	7,405 / 229,773 (3.2%)	1.13 (1.10, 1.16)	<0.001	1.60 (1.55, 1.64)	<0.001
Hispanic or Latino Any Race	3,124 / 208,877 (1.5%)	0.52 (0.50, 0.54)	<0.001	0.81 (0.78, 0.84)	<0.001
Other non-Hispanic	2,337 / 122,343 (1.9%)	0.67 (0.64, 0.70)	<0.001	0.86 (0.82, 0.89)	<0.001
Unknown	1,441 / 90,976 (1.6%)	0.57 (0.54, 0.60)	<0.001	0.80 (0.76, 0.84)	<0.001
White non-Hispanic	39,360 / 1,368,193 (2.9%)	Reference		Reference	
SARS-CoV-2 Variant Period					
Omicron (B.1.1.529, BA.2, BA.2.12.1)	22,732 / 996,967 (2.3%)	Reference		Reference	
Delta (B.1.617.2)	6,035 / 262,362 (2.3%)	1.01 (0.98, 1.04)	0.44	1.03 (1.00, 1.06)	0.05
Omicron (BA.5, BQ.1.1, XBB.1.5)	24,900 / 760,833 (3.3%)	1.49 (1.47, 1.52)	<0.001	1.01 (1.00, 1.03)	0.13
COVID-19 Vaccination Status					
No Documented COVID-19 Vaccination	31,481 / 1,252,584 (2.5%)	Reference		Reference	
Primary COVID-19 Vaccination Documented	9,534 / 394,171 (2.4%)	0.95 (0.93, 0.97)	<0.001	0.95 (0.93, 0.97)	<0.001
Primary and Additional COVID-19 Dose(s) Documented	12,652 / 373,407 (3.4%)	1.34 (1.32, 1.37)	<0.001	0.90 (0.88, 0.92)	<0.001
Heart Failure					
No History of HF	47,178 / 1,959,371 (2.4%)	Reference		Reference	
History of HF	6,489 / 60,791 (11%)	4.69 (4.57, 4.82)	<0.001	1.54 (1.49, 1.59)	<0.001
Diabetes					
No History of Diabetes	39,243 / 1,778,536 (2.2%)	Reference		Reference	
History of Diabetes	14,424 / 241,626 (6.0%)	2.77 (2.72, 2.83)	<0.001	1.45 (1.42, 1.48)	<0.001
Hypertension					
No History of HTN	22,593 / 1,451,392 (1.6%)	Reference		Reference	
History of HTN	31,074 / 568,770 (5.5%)	3.61 (3.55, 3.67)	<0.001	1.32 (1.30, 1.35)	<0.001
Cardiovascular Disease					
No History of CVD	32,920 / 1,681,121 (2.0%)	Reference		Reference	
History of CVD	20,747 / 339,041 (6.1%)	3.23 (3.17, 3.28)	<0.001	1.19 (1.16, 1.21)	<0.001
Obesity					
No History of Obesity	27,796 / 1,196,904 (2.3%)	Reference		Reference	
History of Obesity	25,871 / 823,258 (3.1%)	1.36 (1.34, 1.38)	<0.001	1.13 (1.11, 1.15)	<0.001
Tobacco Usage					
No History of Tobacco Usage	45,337 / 1,755,320 (2.6%)	Reference		Reference	
History of Tobacco Usage	8,330 / 264,842 (3.1%)	1.22 (1.20, 1.25)	<0.001	1.13 (1.10, 1.15)	<0.001
Rurality					
Urban	37,481 / 1,507,908 (2.5%)	Reference		Reference	
Rural	10,366 / 305,903 (3.4%)	1.34 (1.32, 1.37)	<0.001	1.27 (1.24, 1.30)	<0.001
Missing	5,820 / 206,351 (2.8%)	1.15 (1.12, 1.18)	<0.001	1.06 (1.03, 1.09)	<0.001

Supplemental Table 8 includes univariate and multivariable Cox Proportional Hazards for incident CKD/ESKD among patients with a SARS-CoV-2 infection documented between October 1, 2021, and September 30, 2023. Patients are followed up for 365 days for an outcome of CKD. Abbreviations: Number of Events (N Events), Number of Patients (N), hazard ratio (HR), subdistribution hazard ratio (sHR), Coronavirus disease 2019 (COVID-19), acute respiratory syndrome coronavirus (SARS-CoV-2), chronic kidney disease (CKD), end-stage kidney disease (ESKD), acute kidney injury (AKI).

Supplemental Table 9. Descriptive Statistics for Patients with and without Long COVID (Among Patients with at Least 2 eGFR Measurements 30–365 Days Post COVID-19)

Characteristic[1]	Overall, N = 212,470	No Long COVID, N = 207,473	Long COVID, N = 4,997	*p* value[2]
Age at COVID-19	58 (44, 69)	58 (44, 69)	57 (45, 67)	0.20
Age Group				<0.001
18-29	16,656 (7.8%)	16,419 (7.9%)	237 (4.7%)	
30-39	23,699 (11%)	23,127 (11%)	572 (11%)	
40-49	31,929 (15%)	31,061 (15%)	868 (17%)	
50-59	42,249 (20%)	41,140 (20%)	1,109 (22%)	
60-69	49,480 (23%)	48,263 (23%)	1,217 (24%)	
70-79	35,831 (17%)	35,063 (17%)	768 (15%)	
80+	12,626 (5.9%)	12,400 (6.0%)	226 (4.5%)	
Sex				<0.001
Female	135,097 (64%)	131,581 (63%)	3,516 (70%)	
Male	77,373 (36%)	75,892 (37%)	1,481 (30%)	
Race/Ethnicity				<0.001
Black or African American Non-Hispanic	23,756 (11%)	23,272 (11%)	484 (9.7%)	
Hispanic or Latino Any Race	18,193 (8.6%)	17,743 (8.6%)	450 (9.0%)	
Other non-Hispanic	11,354 (5.3%)	11,139 (5.4%)	215 (4.3%)	
Unknown	6,834 (3.2%)	6,674 (3.2%)	160 (3.2%)	
White non-Hispanic	152,333 (72%)	148,645 (72%)	3,688 (74%)	
Rurality				0.34
Urban	153,767 (72%)	150,105 (72%)	3,662 (73%)	
Rural	37,511 (18%)	36,661 (18%)	850 (17%)	
Missing	21,192 (10.0%)	20,707 (10.0%)	485 (9.7%)	
COVID-19 Vaccination Status				<0.001
No Documented COVID-19 Vaccination	117,763 (55%)	114,662 (55%)	3,101 (62%)	
Primary COVID-19 Vaccination Documented	41,653 (20%)	40,758 (20%)	895 (18%)	
Primary and Additional COVID-19 Dose(s) Documented	53,054 (25%)	52,053 (25%)	1,001 (20%)	
SARS-CoV-2 Variant-Dominant Period				<0.001
Delta (B.1.617.2)	22,756 (11%)	21,898 (11%)	858 (17%)	
Omicron (B.1.1.529, BA.2, BA.2.12.1)	96,577 (45%)	94,170 (45%)	2,407 (48%)	
Omicron (BA.5, BQ.1.1, XBB.1.5)	93,137 (44%)	91,405 (44%)	1,732 (35%)	
Heart Failure Before COVID-19	16,815 (7.9%)	16,373 (7.9%)	442 (8.8%)	0.01
Cardiovascular Disease Before COVID-19	74,307 (35%)	72,281 (35%)	2,026 (41%)	<0.001
Hypertension Before COVID-19	112,652 (53%)	109,865 (53%)	2,787 (56%)	<0.001
Obesity Before COVID-19	115,979 (55%)	112,930 (54%)	3,049 (61%)	<0.001
Tobacco Usage Before COVID-19	36,588 (17%)	35,673 (17%)	915 (18%)	0.04
Diabetes Before COVID-19	55,726 (26%)	54,461 (26%)	1,265 (25%)	0.14
Baseline eGFR Before COVID-19	94 (82, 105)	94 (82, 105)	93 (81, 103)	<0.001
Post-COVID-19 Earliest eGFR	93 (81, 105)	93 (81, 105)	93 (81, 104)	0.05
Post-COVID-19 Latest eGFR	92 (80, 104)	92 (80, 104)	91 (79, 102)	<0.001
Post-COVID-19 Average eGFR	92 (81, 103)	92 (81, 103)	92 (81, 102)	<0.001
Change in eGFR Post-COVID	-1 (-6, 4)	-1 (-6, 4)	-1 (-8, 4)	<0.001
Adverse post-COVID-19 Events				
eGFR Decline - 28+ Days after Acute COVID-19 (90+ Days Apart)	13,671 (6.4%)	13,275 (6.4%)	396 (7.9%)	<0.001
Death - 28+ Days After Acute COVID-19 (With two Creatinine Measures 90+ Days Apart, No eGFR Decline)	1,884 (0.9%)	1,844 (0.9%)	40 (0.8%)	0.51
Any Death After COVID-19	2,322 (1.1%)	2,268 (1.1%)	54 (1.1%)	0.93

1. n (%); Median (IQR)
2. Pearson's Chi-squared test; Wilcoxon rank sum test

Supplemental Table 9 provides descriptive statistics for patients with a documented SARS-CoV-2 infection between October 1, 2021, and September 30, 2023. This includes all patients seen at clinics with >=250 long COVID cases based on study inclusion/exclusion criteria. All patients in this cohort must have at least 2 eGFR measurements (at least 90 days apart) documented 30 days after initial SARS-CoV-2 infection. Abbreviations: Coronavirus disease 2019 (COVID-19), acute respiratory syndrome coronavirus (SARS-CoV-2), chronic kidney disease (CKD), end-stage kidney disease (ESKD), acute kidney injury (AKI**)**.

Supplemental Table 10. Descriptive Statistics for Patients with and without Long COVID after PSM (Among Patients with at least 2 eGFR Measurements between 30 and 365 days of Acute COVID-19)

Characteristic[1]	Overall, N = 9,980	No Long COVID, N = 4,990	Long COVID, N = 4,990	SMD (95% CI)[2]
Age at COVID-19	57 (44, 67)	57 (44, 67)	57 (44, 67)	-0.03 (-0.07, 0.01)
Age Group				0.03 (-0.01, 0.07)
18-29	495 (5.0%)	258 (5.2%)	237 (4.7%)	
30-39	1,172 (12%)	600 (12%)	572 (11%)	
40-49	1,744 (17%)	878 (18%)	866 (17%)	
50-59	2,197 (22%)	1,091 (22%)	1,106 (22%)	
60-69	2,426 (24%)	1,210 (24%)	1,216 (24%)	
70-79	1,512 (15%)	745 (15%)	767 (15%)	
80+	434 (4.3%)	208 (4.2%)	226 (4.5%)	
Sex				0.00 (-0.04, 0.04)
Female	7,024 (70%)	3,512 (70%)	3,512 (70%)	
Male	2,956 (30%)	1,478 (30%)	1,478 (30%)	
Race/Ethnicity				0.00 (-0.04, 0.04)
Black or African American Non-Hispanic	966 (9.7%)	483 (9.7%)	483 (9.7%)	
Hispanic or Latino Any Race	898 (9.0%)	449 (9.0%)	449 (9.0%)	
Other non-Hispanic	424 (4.2%)	212 (4.2%)	212 (4.2%)	
Unknown	316 (3.2%)	158 (3.2%)	158 (3.2%)	
White non-Hispanic	7,376 (74%)	3,688 (74%)	3,688 (74%)	
Rurality				0.03 (-0.01, 0.07)
Urban	7,263 (73%)	3,606 (72%)	3,657 (73%)	
Rural	1,758 (18%)	909 (18%)	849 (17%)	
Missing	959 (9.6%)	475 (9.5%)	484 (9.7%)	
COVID-19 Vaccination Status				0.06 (0.02, 0.10)
No Documented COVID-19 Vaccination	6,058 (61%)	2,961 (59%)	3,097 (62%)	
Primary COVID-19 Vaccination Documented	1,862 (19%)	969 (19%)	893 (18%)	
Primary and Additional COVID-19 Dose(s) Documented	2,060 (21%)	1,060 (21%)	1,000 (20%)	
SARS-CoV-2 Variant-Dominant Period				0.00 (-0.04, 0.04)
Delta (B.1.617.2)	1,706 (17%)	853 (17%)	853 (17%)	
Omicron (B.1.1.529, BA.2, BA.2.12.1)	4,810 (48%)	2,405 (48%)	2,405 (48%)	
Omicron (BA.5, BQ.1.1, XBB.1.5)	3,464 (35%)	1,732 (35%)	1,732 (35%)	
Heart Failure Before COVID-19	852 (8.5%)	410 (8.2%)	442 (8.9%)	-0.02 (-0.06, 0.02)
Cardiovascular Disease Before COVID-19	3,836 (38%)	1,815 (36%)	2,021 (41%)	-0.08 (-0.12, -0.05)
Hypertension Before COVID-19	5,423 (54%)	2,641 (53%)	2,782 (56%)	-0.06 (-0.10, -0.02)
Obesity Before COVID-19	5,938 (59%)	2,892 (58%)	3,046 (61%)	-0.06 (-0.10, -0.02)
Tobacco Usage Before COVID-19	1,874 (19%)	960 (19%)	914 (18%)	0.02 (-0.02, 0.06)
Diabetes Before COVID-19	2,617 (26%)	1,354 (27%)	1,263 (25%)	0.04 (0.00, 0.08)
Baseline eGFR Before COVID-19	93 (82, 104)	94 (83, 105)	93 (81, 103)	0.07 (0.03, 0.11)
Post-COVID-19 Earliest eGFR	93 (82, 104)	94 (82, 104)	93 (81, 104)	0.04 (0.00, 0.07)
Post-COVID-19 Latest eGFR	92 (80, 103)	93 (80, 103)	91 (79, 102)	0.08 (0.04, 0.12)
Post-COVID-19 Average eGFR	92 (81, 102)	93 (82, 103)	92 (81, 102)	0.06 (0.02, 0.10)
Change in eGFR Post-COVID	-1 (-7, 4)	-1 (-7, 4)	-1 (-8, 4)	0.06 (0.03, 0.10)
Adverse post-COVID-19 Events				
eGFR Decline - 28+ Days after Acute COVID-19 (90+ Days Apart)	721 (7.2%)	326 (6.5%)	395 (7.9%)	-0.05 (-0.09, -0.01)
Death - 28+ Days After Acute COVID-19 (With two Creatinine Measures 90+ Days Apart, No eGFR Decline)	71 (0.7%)	31 (0.6%)	40 (0.8%)	-0.02 (-0.06, 0.02)
Any Death After COVID-19	98 (1.0%)	44 (0.9%)	54 (1.1%)	-0.02 (-0.06, 0.02)

1. n (%); Median (IQR)
2. Standardized mean differences (SMD) with 95% confidence intervals (CI)

Supplemental Table 10 provides descriptive statistics for patients with a documented SARS-CoV-2 infection between October 1, 2021, and September 30, 2023, after propensity-score matching (PSM). This includes all patients seen at clinics with >=250 long COVID cases based on study inclusion/exclusion criteria. All patients in this cohort must have at least 2 eGFR measurements (at least 90 days apart) documented 30 days after initial SARS-CoV-2 infection. Abbreviations: Coronavirus disease 2019 (COVID-19), acute respiratory syndrome coronavirus (SARS-CoV-2), chronic kidney disease (CKD), end-stage kidney disease (ESKD), acute kidney injury (AKI).

Supplemental Table 11. Univariate and Multivariable Cox Proportional Hazards Regression for eGFR Decline (Among Patients with at Least 2 eGFR Measurements)

Characteristic	N Events / N (%)	Unadjusted HR (95% CI)	*p* value	Adjusted sHR (95% CI)	*p* value
COVID-19 Status					
Acute COVID-19	13,275 / 207,473 (6.4%)	Reference		Reference	
Long COVID	396 / 4,997 (7.9%)	1.24 (1.12, 1.37)	<0.001	1.21 (1.10, 1.34)	<0.001
Sex					
Female	8,429 / 135,097 (6.2%)	Reference		Reference	
Male	5,242 / 77,373 (6.8%)	1.09 (1.05, 1.13)	<0.001	1.04 (1.01, 1.08)	0.02
Age Group					
60-69	3,243 / 49,480 (6.6%)	Reference		Reference	
18-29	865 / 16,656 (5.2%)	0.78 (0.73, 0.84)	<0.001	0.84 (0.77, 0.91)	<0.001
30-39	1,409 / 23,699 (5.9%)	0.90 (0.85, 0.96)	0.001	0.94 (0.88, 1.01)	0.08
40-49	1,986 / 31,929 (6.2%)	0.94 (0.89, 1.00)	0.04	0.97 (0.92, 1.03)	0.29
50-59	2,770 / 42,249 (6.6%)	1.00 (0.95, 1.05)	0.94	1.01 (0.96, 1.06)	0.82
70-79	2,439 / 35,831 (6.8%)	1.04 (0.99, 1.10)	0.12	1.04 (0.99, 1.10)	0.14
80+	959 / 12,626 (7.6%)	1.17 (1.09, 1.26)	<0.001	1.15 (1.07, 1.24)	<0.001
Race/Ethnicity					
Black or African American Non-Hispanic	1,851 / 23,756 (7.8%)	1.21 (1.15, 1.27)	<0.001	1.25 (1.19, 1.32)	<0.001
Hispanic or Latino Any Race	973 / 18,193 (5.3%)	0.83 (0.78, 0.89)	<0.001	0.88 (0.82, 0.94)	<0.001
Other non-Hispanic	573 / 11,354 (5.0%)	0.79 (0.72, 0.86)	<0.001	0.82 (0.75, 0.89)	<0.001
Unknown	351 / 6,834 (5.1%)	0.82 (0.73, 0.91)	<0.001	0.86 (0.77, 0.96)	0.007
White non-Hispanic	9,923 / 152,333 (6.5%)	Reference		Reference	
SARS-CoV-2 Variant Period					
Omicron (B.1.1.529, BA.2, BA.2.12.1)	5,986 / 96,577 (6.2%)	Reference		Reference	
Delta (B.1.617.2)	1,652 / 22,756 (7.3%)	1.17 (1.11, 1.23)	<0.001	1.14 (1.08, 1.20)	<0.001
Omicron (BA.5, BQ.1.1, XBB.1.5)	6,033 / 93,137 (6.5%)	1.08 (1.04, 1.12)	<0.001	1.06 (1.03, 1.10)	0.001
COVID-19 Vaccination Status					
No Documented COVID-19 Vaccination	7,891 / 117,763 (6.7%)	Reference		Reference	
Primary COVID-19 Vaccination Documented	2,591 / 41,653 (6.2%)	0.92 (0.88, 0.96)	<0.001	0.91 (0.87, 0.96)	<0.001
Primary and Additional COVID-19 Dose(s) Documented	3,189 / 53,054 (6.0%)	0.89 (0.85, 0.92)	<0.001	0.86 (0.83, 0.90)	<0.001
Heart Failure					
No History of HF	12,012 / 195,655 (6.1%)	Reference		Reference	
History of HF	1,659 / 16,815 (9.9%)	1.65 (1.56, 1.73)	<0.001	1.40 (1.32, 1.48)	<0.001
Diabetes					
No History of Diabetes	9,631 / 156,744 (6.1%)	Reference		Reference	
History of Diabetes	4,040 / 55,726 (7.2%)	1.19 (1.14, 1.23)	<0.001	1.09 (1.05, 1.14)	<0.001
Hypertension					
No History of HTN	5,802 / 99,818 (5.8%)	Reference		Reference	
History of HTN	7,869 / 112,652 (7.0%)	1.21 (1.17, 1.25)	<0.001	1.03 (0.99, 1.07)	0.20
Cardiovascular Disease					
No History of CVD	8,074 / 138,163 (5.8%)	Reference		Reference	
History of CVD	5,597 / 74,307 (7.5%)	1.31 (1.26, 1.35)	<0.001	1.12 (1.07, 1.16)	<0.001
Obesity					
No History of Obesity	5,824 / 96,491 (6.0%)	Reference		Reference	
History of Obesity	7,847 / 115,979 (6.8%)	1.12 (1.09, 1.16)	<0.001	1.06 (1.02, 1.10)	0.002
Tobacco Usage					
No History of Tobacco Usage	10,984 / 175,882 (6.2%)	Reference		Reference	
History of Tobacco Usage	2,687 / 36,588 (7.3%)	1.18 (1.13, 1.23)	<0.001	1.13 (1.08, 1.18)	<0.001
Rurality					
Urban	9,647 / 153,767 (6.3%)	Reference		Reference	
Rural	2,675 / 37,511 (7.1%)	1.12 (1.07, 1.17)	<0.001	1.11 (1.06, 1.16)	<0.001
Missing	1,349 / 21,192 (6.4%)	1.03 (0.97, 1.09)	0.34	1.00 (0.94, 1.06)	0.99

Supplemental Table 11 includes univariate and multivariable Cox Proportional Hazards for eGFR decline among patients with a SARS-CoV-2 infection documented between October 1, 2021, and September 30, 2023. eGFR decline is assessed using the earliest post-COVID-19 eGFR measurement after infection (>= 30 days) and the last post-COVID-19 eGFR measurement taken within 365 days. These measurements must be at least 90 days apart. A decrease of >=20% is used as the threshold for eGFR decline. Abbreviations: Number of Events (N Events), Number of Patients (N), hazard ratio (HR), subdistribution hazard ratio (sHR), Coronavirus disease 2019 (COVID-19), acute respiratory syndrome coronavirus (SARS-CoV-2), chronic kidney disease (CKD), end-stage kidney disease (ESKD), acute kidney injury (AKI).

Supplemental Table 12. Univariate and Multivariable Cox Proportional Hazards Regression for eGFR Decline after PSM (Among Patients with at Least 2 eGFR Measurements)

Characteristic	N Events / N (%)	Unadjusted HR (95% CI)	*p* value	Adjusted sHR (95% CI)	*p* value
COVID-19 Status					
Acute COVID-19	326 / 4,990 (6.5%)	Reference		Reference	
Long COVID	395 / 4,990 (7.9%)	1.22 (1.05, 1.41)	0.008	1.21 (1.04, 1.40)	0.01
Sex					
Female	466 / 7,024 (6.6%)	Reference		Reference	
Male	255 / 2,956 (8.6%)	1.30 (1.12, 1.51)	<0.001	1.23 (1.05, 1.44)	0.01
Age Group					
60-69	206 / 2,426 (8.5%)	Reference		Reference	
18-29	24 / 495 (4.8%)	0.56 (0.37, 0.86)	0.008	0.61 (0.39, 0.95)	0.03
30-39	68 / 1,172 (5.8%)	0.67 (0.51, 0.89)	0.005	0.70 (0.52, 0.93)	0.01
40-49	125 / 1,744 (7.2%)	0.84 (0.68, 1.05)	0.13	0.89 (0.70, 1.12)	0.32
50-59	152 / 2,197 (6.9%)	0.80 (0.65, 0.99)	0.04	0.80 (0.65, 0.99)	0.04
70-79	119 / 1,512 (7.9%)	0.92 (0.73, 1.15)	0.46	0.94 (0.75, 1.17)	0.57
80+	27 / 434 (6.2%)	0.73 (0.49, 1.09)	0.12	0.73 (0.49, 1.10)	0.13
Race/Ethnicity					
Black or African American Non-Hispanic	81 / 966 (8.4%)	1.15 (0.91, 1.45)	0.24	1.23 (0.97, 1.57)	0.09
Hispanic or Latino Any Race	53 / 898 (5.9%)	0.81 (0.61, 1.07)	0.14	0.88 (0.65, 1.17)	0.36
Other non-Hispanic	25 / 424 (5.9%)	0.81 (0.55, 1.22)	0.32	0.90 (0.60, 1.35)	0.62
Unknown	20 / 316 (6.3%)	0.88 (0.56, 1.38)	0.58	0.99 (0.63, 1.56)	0.98
White non-Hispanic	542 / 7,376 (7.3%)	Reference	White non-Hispanic	542 / 7,376 (7.3%)	Reference
SARS-CoV-2 Variant Period					
Omicron (B.1.1.529, BA.2, BA.2.12.1)	337 / 4,810 (7.0%)	Reference		Reference	
Delta (B.1.617.2)	158 / 1,706 (9.3%)	1.33 (1.10, 1.61)	0.003	1.27 (1.05, 1.54)	0.02
Omicron (BA.5, BQ.1.1, XBB.1.5)	226 / 3,464 (6.5%)	0.96 (0.81, 1.14)	0.66	0.95 (0.80, 1.13)	0.56
COVID-19 Vaccination Status					
No Documented COVID-19 Vaccination	451 / 6,058 (7.4%)	Reference		Reference	
Primary COVID-19 Vaccination Documented	133 / 1,862 (7.1%)	0.95 (0.78, 1.15)	0.60	0.94 (0.77, 1.14)	0.51
Primary and Additional COVID-19 Dose(s) Documented	137 / 2,060 (6.7%)	0.88 (0.73, 1.07)	0.20	0.93 (0.76, 1.14)	0.49
Heart Failure					
No History of HF	626 / 9,128 (6.9%)	Reference		Reference	
History of HF	95 / 852 (11%)	1.68 (1.36, 2.09)	<0.001	1.36 (1.07, 1.74)	0.012
Diabetes					
No History of Diabetes	512 / 7,363 (7.0%)	Reference		Reference	
History of Diabetes	209 / 2,617 (8.0%)	1.15 (0.98, 1.35)	0.09	1.01 (0.85, 1.20)	0.89
Hypertension					
No History of HTN	300 / 4,557 (6.6%)	Reference		Reference	

History of HTN	421 / 5,423 (7.8%)	1.18 (1.02, 1.37)	0.03	0.94 (0.79, 1.12)	0.53
Cardiovascular Disease					
No History of CVD	390 / 6,144 (6.3%)	Reference		Reference	
History of CVD	331 / 3,836 (8.6%)	1.38 (1.19, 1.60)	<0.001	1.20 (1.01, 1.42)	0.04
Obesity					
No History of Obesity	258 / 4,042 (6.4%)	Reference		Reference	
History of Obesity	463 / 5,938 (7.8%)	1.23 (1.05, 1.43)	0.009	1.17 (1.00, 1.39)	0.06
Tobacco Usage					
No History of Tobacco Usage	558 / 8,106 (6.9%)	Reference		Reference	
History of Tobacco Usage	163 / 1,874 (8.7%)	1.27 (1.07, 1.51)	0.007	1.21 (1.02, 1.45)	0.03
Rurality					
Urban	511 / 7,263 (7.0%)	Reference		Reference	
Rural	145 / 1,758 (8.2%)	1.16 (0.97, 1.40)	0.11	1.10 (0.91, 1.33)	0.33
Missing	65 / 959 (6.8%)	0.98 (0.76, 1.27)	0.88	0.98 (0.76, 1.27)	0.88

Supplemental Table 12 includes univariate and multivariable Cox Proportional Hazards for eGFR decline among patients with a SARS-CoV-2 infection documented between October 1, 2021, and September 30, 2023, after propensity-score matching (PSM). Patients were PSM using logistic regression with 1:1 matching for binary long COVID status with exact matching on sex, race/ethnicity, COVID-19 variant period, and data-contributing site and nearest neighbor PSM on age. eGFR decline is assessed using the earliest post-COVID-19 eGFR measurement after infection (>= 30 days) and the last post-COVID-19 eGFR measurement taken within 365 days. These measurements must be at least 90 days apart. A decrease of >=20% is used as the threshold for eGFR decline. Abbreviations: Number of Events (N Events), Number of Patients (N), hazard ratio (HR), subdistribution hazard ratio (sHR), Coronavirus disease 2019 (COVID-19), acute respiratory syndrome coronavirus (SARS-CoV-2), chronic kidney disease (CKD), end-stage kidney disease (ESKD), acute kidney injury (AKI).

Supplemental Table 13. Descriptive Statistics for Patients with and without CKD/ESKD after PSM (baseline CKD based on the 2 eGFR criteria - Sensitivity Analysis 1).

Characteristic[1]	Overall, N = 523,620	No Baseline CKD/ESKD, N = 261,810	Baseline CKD/ESKD, N = 261,810	SMD (95% CI)[2]
Age at COVID-19	71 (61, 80)	71 (61, 79)	71 (62, 80)	-0.03 (-0.04, -0.02)
Age Group				0.04 (0.03, 0.05)
60-69	5,884 (1.1%)	2,967 (1.1%)	2,917 (1.1%)	
18-29	13,950 (2.7%)	7,010 (2.7%)	6,940 (2.7%)	
30-39	27,918 (5.3%)	14,000 (5.3%)	13,918 (5.3%)	
40-49	64,016 (12%)	32,144 (12%)	31,872 (12%)	
50-59	122,121 (23%)	61,853 (24%)	60,268 (23%)	
70-79	158,601 (30%)	80,489 (31%)	78,112 (30%)	
80+	131,130 (25%)	63,347 (24%)	67,783 (26%)	
Sex				0.00 (-0.01, 0.01)
Female	286,014 (55%)	143,007 (55%)	143,007 (55%)	
Male	237,606 (45%)	118,803 (45%)	118,803 (45%)	
Race/Ethnicity				0.00 (-0.01, 0.01)
Black or African American Non-Hispanic	85,970 (16%)	42,985 (16%)	42,985 (16%)	
Hispanic or Latino Any Race	32,412 (6.2%)	16,206 (6.2%)	16,206 (6.2%)	
Other non-Hispanic	28,460 (5.4%)	14,230 (5.4%)	14,230 (5.4%)	
Unknown	12,660 (2.4%)	6,330 (2.4%)	6,330 (2.4%)	
White non-Hispanic	364,118 (70%)	182,059 (70%)	182,059 (70%)	
Rurality				0.03 (0.03, 0.04)
Urban	378,145 (72%)	190,644 (73%)	187,501 (72%)	
Rural	85,898 (16%)	41,331 (16%)	44,567 (17%)	
Missing	*59,577 (11%)*	*29,835 (11%)*	*29,742 (11%)*	
COVID-19 Vaccination Status				0.08 (0.08, 0.09)
No Documented COVID-19 Vaccination	285,938 (55%)	148,292 (57%)	137,646 (53%)	
Primary COVID-19 Vaccination Documented	90,963 (17%)	43,246 (17%)	47,717 (18%)	
Primary and Additional COVID-19 Dose(s) Documented	146,719 (28%)	70,272 (27%)	76,447 (29%)	
SARS-CoV-2 Variant-Dominant Period				0.00 (-0.01, 0.01)
Delta (B.1.617.2)	46,288 (8.8%)	23,144 (8.8%)	23,144 (8.8%)	
Omicron (B.1.1.529, BA.2, BA.2.12.1)	207,478 (40%)	103,739 (40%)	103,739 (40%)	
Omicron (BA.5, BQ.1.1, XBB.1.5)	269,854 (52%)	134,927 (52%)	134,927 (52%)	
Heart Failure Before COVID-19	104,370 (20%)	24,755 (9.5%)	79,615 (30%)	
Cardiovascular Disease Before COVID-19	265,421 (51%)	97,548 (37%)	167,873 (64%)	
Hypertension Before COVID-19	363,507 (69%)	141,948 (54%)	221,559 (85%)	
Obesity Before COVID-19	258,651 (49%)	106,866 (41%)	151,785 (58%)	
Tobacco Usage Before COVID-19	77,903 (15%)	33,740 (13%)	44,163 (17%)	
Diabetes Before COVID-19	174,594 (33%)	53,807 (21%)	120,787 (46%)	
Baseline eGFR Before COVID-19	66 (50, 84)	83 (72, 93)	54 (41, 66)	1.50 (1.50, 1.50)
No eGFR Available in the Year Before COVID-19	*162,644*	*114,223*	*48,421*	
Adverse post-COVID-19 Events				
Long COVID	8,510 (1.6%)	3,600 (1.4%)	4,910 (1.9%)	-0.04 (-0.04, -0.03)
Death After COVID-19 (with no Long COVID diagnosis)	26,588 (5.1%)	8,554 (3.3%)	18,034 (6.9%)	-0.17 (-0.17, -0.16)
Any Death After COVID-19	27,181 (5.2%)	8,721 (3.3%)	18,460 (7.1%)	-0.17 (-0.17, -0.16)

1. n (%); Median (IQR)
2. Standardized mean differences (SMD) with 95% confidence intervals (CI)

Supplemental Table 13 provides descriptive statistics for patients with a documented SARS-CoV-2 infection between October 1, 2021, and September 30, 2023, among patients with at least 2 eGFR measurements for eGFR-qualifying CKD (sensitivity analysis 1) after propensity-score matching (PSM). Patients were PSM using logistic regression with 1:1 matching for binary long COVID status with exact matching on sex, race/ethnicity, COVID-19 variant period, and data-contributing site and nearest neighbor PSM on age. Abbreviations: Coronavirus disease 2019 (COVID-19), acute respiratory syndrome coronavirus (SARS-CoV-2), chronic kidney disease (CKD), end-stage kidney disease (ESKD), acute kidney injury (AKI).

Supplemental Table 14. Univariate and Multivariable Cox Proportional Hazards Regression for Long COVID After PSM (Sensitivity Analysis 1)

Characteristic	Unadjusted cHR (95% CI)	*p* value	Adjusted sHR (95% CI)	*p* value
CKD/ESKD Status				
No baseline CKD/ESKD				
Baseline CKD/ESKD	1.37 (1.31, 1.43)	<0.001	1.17 (1.12, 1.23)	<0.001
Sex				
Female	Reference		Reference	
Male	0.83 (0.79, 0.86)	<0.001	0.79 (0.76, 0.83)	<0.001
Age Group				
60-69	Reference		Reference	
18-29	0.45 (0.34, 0.59)	<0.001	0.48 (0.36, 0.63)	<0.001
30-39	0.73 (0.63, 0.84)	<0.001	0.76 (0.65, 0.88)	<0.001
40-49	1.02 (0.92, 1.12)	0.74	1.02 (0.93, 1.12)	0.68
50-59	0.98 (0.91, 1.05)	0.60	0.98 (0.91, 1.05)	0.53
70-79	0.89 (0.84, 0.94)	<0.001	0.90 (0.85, 0.95)	<0.001
80+	0.70 (0.66, 0.75)	<0.001	0.73 (0.69, 0.78)	<0.001
Race/Ethnicity				
Black or African American Non-Hispanic	0.89 (0.84, 0.95)	<0.001	0.81 (0.76, 0.87)	<0.001
Hispanic or Latino Any Race	1.01 (0.92, 1.10)	0.91	0.99 (0.90, 1.08)	0.83
Other non-Hispanic	0.78 (0.70, 0.86)	<0.001	0.85 (0.76, 0.94)	0.002
Unknown	0.95 (0.82, 1.09)	0.44	1.01 (0.88, 1.16)	0.88
White non-Hispanic	Reference		Reference	
SARS-CoV-2 Variant Period				
Omicron (B.1.1.529, BA.2, BA.2.12.1)	Reference		Reference	
Delta (B.1.617.2)	1.58 (1.48, 1.68)	<0.001	1.54 (1.44, 1.64)	<0.001
Omicron (BA.5, BQ.1.1, XBB.1.5)	0.69 (0.65, 0.72)	<0.001	0.69 (0.65, 0.72)	<0.001
COVID-19 Vaccination Status				
No Documented COVID-19 Vaccination	Reference		Reference	
Primary COVID-19 Vaccination Documented	0.92 (0.87, 0.98)	0.005	0.85 (0.80, 0.90)	<0.001
Primary and Additional COVID-19 Dose(s) Documented	0.77 (0.73, 0.81)	<0.001	0.87 (0.82, 0.92)	<0.001
Heart Failure				
No History of HF	Reference		Reference	
History of HF	1.31 (1.25, 1.38)	<0.001	1.05 (0.99, 1.11)	0.12
Diabetes				
No History of Diabetes	Reference		Reference	
History of Diabetes	1.19 (1.14, 1.24)	<0.001	0.95 (0.91, 1.00)	0.05
Hypertension				
No History of HTN	Reference		Reference	
History of HTN	1.37 (1.31, 1.44)	<0.001	1.15 (1.08, 1.22)	<0.001
Cardiovascular Disease				
No History of CVD	Reference		Reference	
History of CVD	1.37 (1.31, 1.43)	<0.001	1.28 (1.21, 1.35)	<0.001
Obesity				
No History of Obesity	Reference		Reference	
History of Obesity	1.55 (1.49, 1.62)	<0.001	1.36 (1.30, 1.43)	<0.001
Tobacco Usage				
No History of Tobacco Usage	Reference		Reference	
History of Tobacco Usage	1.17 (1.11, 1.24)	<0.001	1.08 (1.02, 1.14)	0.01
Rurality				
Urban	Reference		Reference	
Rural	1.10 (1.04, 1.17)	<0.001	1.04 (0.98, 1.10)	0.18
Missing	1.02 (0.95, 1.09)	0.61	0.99 (0.92, 1.06)	0.73

Supplmenetal Table 14 includes univariate and multivariable Cox Proportional Hazards for long COVID (U09.9) among patients with a SARS-CoV-2 infection documented between October 1, 2021, and September 30, 2023, among patients with at least 2 eGFR measurements for eGFR-qualifying CKD (sensitivity analysis 1) after propensity-score matching (PSM). Patients were PSM using logistic regression with 1:1 matching for binary long COVID status with exact matching on sex, race/ethnicity, COVID-19 variant period, and data-contributing site and nearest neighbor PSM on age. Abbreviations: hazard ratio (HR), subdistribution hazard ratio (sHR), Coronavirus disease 2019 (COVID-19), acute respiratory syndrome coronavirus (SARS-CoV-2), chronic kidney disease (CKD), end-stage kidney disease (ESKD), acute kidney injury (AKI).

Supplemental Table 15. Descriptive Statistics Among Patients with and without Long COVID after PSM (incident CKD based on the 2 eGFR criteria - Sensitivity Analysis 1).

Characteristic[1]	Overall, N = 48,518	No Long COVID, N = 24,259	Long COVID, N = 24,259	SMD (95% CI)[2]
Age at COVID-19	53 (41, 64)	53 (41, 64)	53 (41, 64)	-0.01 (-0.02, 0.01)
Age Group				0.01 (-0.01, 0.03)
18-29	4,109 (8.0%)	2,064 (8.0%)	2,045 (7.9%)	
30-39	7,517 (15%)	3,760 (15%)	3,757 (15%)	
40-49	10,095 (20%)	5,057 (20%)	5,038 (20%)	
50-59	11,616 (23%)	5,809 (23%)	5,807 (23%)	
60-69	10,388 (20%)	5,199 (20%)	5,189 (20%)	
70-79	5,838 (11%)	2,910 (11%)	2,928 (11%)	
80+	2,021 (3.9%)	993 (3.9%)	1,028 (4.0%)	
Sex				0.00 (-0.02, 0.02)
Female	35,374 (69%)	17,687 (69%)	17,687 (69%)	
Male	16,210 (31%)	8,105 (31%)	8,105 (31%)	
Race/Ethnicity				0.00 (-0.02, 0.02)
Black or African American Non-Hispanic	4,908 (9.5%)	2,454 (9.5%)	2,454 (9.5%)	
Hispanic or Latino Any Race	5,004 (9.7%)	2,502 (9.7%)	2,502 (9.7%)	
Other non-Hispanic	2,792 (5.4%)	1,396 (5.4%)	1,396 (5.4%)	
Unknown	2,210 (4.3%)	1,105 (4.3%)	1,105 (4.3%)	
White non-Hispanic	36,670 (71%)	18,335 (71%)	18,335 (71%)	
Rurality				0.01 (-0.01, 0.02)
Urban	38,182 (74%)	19,108 (74%)	19,074 (74%)	
Rural	7,729 (15%)	3,871 (15%)	3,858 (15%)	
Missing	*5,673 (11%)*	*2,813 (11%)*	*2,860 (11%)*	
COVID-19 Vaccination Status				0.05 (0.04, 0.07)
No Documented COVID-19 Vaccination	33,673 (65%)	16,572 (64%)	17,101 (66%)	
Primary COVID-19 Vaccination Documented	9,053 (18%)	4,788 (19%)	4,265 (17%)	
Primary and Additional COVID-19 Dose(s) Documented	8,858 (17%)	4,432 (17%)	4,426 (17%)	
SARS-CoV-2 Variant-Dominant Period				0.00 (-0.02, 0.02)
Delta (B.1.617.2)	9,844 (19%)	4,922 (19%)	4,922 (19%)	
Omicron (B.1.1.529, BA.2, BA.2.12.1)	24,344 (47%)	12,172 (47%)	12,172 (47%)	
Omicron (BA.5, BQ.1.1, XBB.1.5)	17,396 (34%)	8,698 (34%)	8,698 (34%)	
Heart Failure Before COVID-19	2,393 (4.6%)	1,022 (4.0%)	1,371 (5.3%)	-0.06 (-0.08, -0.05)
Cardiovascular Disease Before COVID-19	12,187 (24%)	5,361 (21%)	6,826 (26%)	-0.13 (-0.15, -0.12)
Hypertension Before COVID-19	18,987 (37%)	9,036 (35%)	9,951 (39%)	-0.07 (-0.09, -0.06)
Obesity Before COVID-19	24,866 (48%)	11,677 (45%)	13,189 (51%)	-0.12 (-0.13, -0.10)
Tobacco Usage Before COVID-19	7,906 (15%)	3,994 (15%)	3,912 (15%)	0.01 (-0.01, 0.03)
Diabetes Before COVID-19	7,853 (15%)	3,751 (15%)	4,102 (16%)	-0.04 (-0.06, -0.02)
Baseline eGFR Before COVID-19	92 (79, 104)	92 (80, 104)	92 (79, 104)	0.00 (-0.02, 0.02)
No eGFR Available in the Year Before COVID-19	*24,410*	*13,094*	*11,316*	
Adverse post-COVID-19 Events				
Incident CKD/ESKD	1,206 (2.3%)	398 (1.5%)	808 (3.1%)	-0.11 (-0.12, -0.09)
Death After COVID-19 (with no Long COVID diagnosis)	624 (1.2%)	239 (0.9%)	385 (1.5%)	-0.05 (-0.07, -0.03)
Any Death After COVID-19	704 (1.4%)	258 (1.0%)	446 (1.7%)	-0.06 (-0.08, -0.05)

1. n (%); Median (IQR)
2. Standardized mean differences (SMD) with 95% confidence intervals (CI)

Supplemental Table 15 provides descriptive statistics for patients with a documented SARS-CoV-2 infection between October 1, 2021, and September 30, 2023, among patients with at least 2 eGFR measurements for eGFR-qualifying CKD (sensitivity analysis 1) after propensity-score matching (PSM). Patients were PSM using logistic regression with 1:1 matching for binary long COVID status with exact matching on sex, race/ethnicity, COVID-19 variant period, and data-contributing site and nearest neighbor PSM on age. This includes all patients seen at clinics with >=250 long COVID cases based on study inclusion/exclusion criteria. Abbreviations: Coronavirus disease 2019 (COVID-19), acute respiratory syndrome coronavirus (SARS-CoV-2), chronic kidney disease (CKD), end-stage kidney disease (ESKD), acute kidney injury.

Supplemental Table 16. Univariate and Multivariable Cox Proportional Hazards Regression for Incident CKD/ESKD After PSM (Sensitivity Analysis 1)

Characteristic	Unadjusted HR (95% CI)	*p* value	Adjusted sHR (95% CI)	*p* value
COVID-19 Status				
Acute COVID-19	Reference		Reference	
Long COVID	2.05 (1.81, 2.31)	<0.001	1.93 (1.71, 2.18)	<0.001
Sex				
Female	Reference		Reference	
Male	1.61 (1.43, 1.80)	<0.001	1.29 (1.14, 1.45)	<0.001
Age Group				
60-69	Reference		Reference	
18-29	0.09 (0.05, 0.15)	<0.001	0.12 (0.07, 0.22)	<0.001
30-39	0.16 (0.12, 0.22)	<0.001	0.21 (0.15, 0.30)	<0.001
40-49	0.27 (0.22, 0.34)	<0.001	0.33 (0.26, 0.42)	<0.001
50-59	0.59 (0.50, 0.69)	<0.001	0.64 (0.54, 0.75)	<0.001
70-79	1.61 (1.38, 1.87)	<0.001	1.52 (1.30, 1.78)	<0.001
80+	2.54 (2.11, 3.06)	<0.001	2.18 (1.79, 2.66)	<0.001
Race/Ethnicity				
Black or African American Non-Hispanic	1.18 (0.99, 1.40)	0.07	1.44 (1.20, 1.74)	<0.001
Hispanic or Latino Any Race	0.62 (0.49, 0.78)	<0.001	0.87 (0.68, 1.10)	0.23
Other non-Hispanic	0.65 (0.48, 0.87)	0.004	0.87 (0.64, 1.17)	0.36
Unknown	0.40 (0.26, 0.61)	<0.001	0.57 (0.37, 0.87)	0.009
White non-Hispanic	Reference		Reference	
SARS-CoV-2 Variant Period				
Omicron (B.1.1.529, BA.2, BA.2.12.1)	Reference		Reference	
Delta (B.1.617.2)	1.18 (1.02, 1.37)	0.03	1.12 (0.96, 1.31)	0.14
Omicron (BA.5, BQ.1.1, XBB.1.5)	1.12 (0.98, 1.27)	0.09	0.95 (0.84, 1.08)	0.50
COVID-19 Vaccination Status				
No Documented COVID-19 Vaccination	Reference		Reference	
Primary COVID-19 Vaccination Documented	0.84 (0.72, 0.99)	0.04	0.83 (0.71, 0.98)	0.02
Primary and Additional COVID-19 Dose(s) Documented	0.98 (0.84, 1.14)	0.81	0.78 (0.67, 0.92)	0.002
Heart Failure				
No History of HF	Reference		Reference	
History of HF	4.62 (3.99, 5.36)	<0.001	1.86 (1.56, 2.22)	<0.001
Diabetes				
No History of Diabetes	Reference		Reference	
History of Diabetes	2.49 (2.20, 2.81)	<0.001	1.47 (1.29, 1.69)	<0.001
Hypertension				
No History of HTN	Reference		Reference	
History of HTN	2.85 (2.54, 3.20)	<0.001	1.19 (1.03, 1.36)	0.02
Cardiovascular Disease				
No History of CVD	Reference		Reference	
History of CVD	2.98 (2.66, 3.34)	<0.001	1.30 (1.13, 1.49)	<0.001
Obesity				
No History of Obesity	Reference		Reference	
History of Obesity	1.36 (1.22, 1.53)	<0.001	1.13 (1.0, 1.27)	0.06
Tobacco Usage				
No History of Tobacco Usage	Reference		Reference	
History of Tobacco Usage	0.84 (0.71, 1.0)	0.04	0.78 (0.66, 0.93)	0.004
Rurality				
Urban	Reference		Reference	
Rural	1.36 (1.18, 1.57)	<0.001	1.17 (1.00, 1.35)	0.04
Missing	1.07 (0.89, 1.28)	0.48	1.04 (0.87, 1.26)	0.67

Supplemental Table 16 includes univariate and multivariable Cox Proportional Hazards for incident CKD/ESKD among patients with a SARS-CoV-2 infection documented between October 1, 2021, and September 30, 2023, among patients with at least 2 eGFR measurements for eGFR-qualifying CKD (sensitivity analysis 1) after propensity-score matching (PSM). Patients are followed up for 365 days for an outcome of CKD. Abbreviations: hazard ratio (HR), subdistribution hazard ratio (sHR), Coronavirus disease 2019 (COVID-19), acute respiratory syndrome coronavirus (SARS-CoV-2), chronic kidney disease (CKD), end-stage kidney disease (ESKD), acute kidney injury (AKI).

Supplemental Table 17. Descriptive Statistics of Patients with and without Long COVID After PSM (Among Patients with at Least 2 eGFR Measurements) (Sensitivity Analysis 1)

Characteristic[1]	Overall, N = 11,272	No Long COVID, N = 5,636	Long COVID, N = 5,636	SMD (95% CI)[2]
Age at COVID-19	58 (46, 68)	58 (45, 68)	58 (46, 68)	-0.03 (-0.07, 0.01)
Age Group				0.03 (-0.01, 0.07)
18-29	503 (4.5%)	264 (4.7%)	239 (4.2%)	
30-39	1,198 (11%)	607 (11%)	591 (10%)	
40-49	1,842 (16%)	927 (16%)	915 (16%)	
50-59	2,464 (22%)	1,227 (22%)	1,237 (22%)	
60-69	2,846 (25%)	1,422 (25%)	1,424 (25%)	
70-79	1,824 (16%)	900 (16%)	924 (16%)	
80+	595 (5.3%)	289 (5.1%)	306 (5.4%)	
Sex				0.00 (-0.04, 0.04)
Female	7,946 (70%)	3,973 (70%)	3,973 (70%)	
Male	3,326 (30%)	1,663 (30%)	1,663 (30%)	
Race/Ethnicity				0.00 (-0.04, 0.04)
Black or African American Non-Hispanic	1,130 (10%)	565 (10%)	565 (10%)	
Hispanic or Latino Any Race	972 (8.6%)	486 (8.6%)	486 (8.6%)	
Other non-Hispanic	462 (4.1%)	231 (4.1%)	231 (4.1%)	
Unknown	342 (3.0%)	171 (3.0%)	171 (3.0%)	
White non-Hispanic	8,366 (74%)	4,183 (74%)	4,183 (74%)	
Rurality				0.01 (-0.03, 0.05)
Urban	8,194 (73%)	4,085 (72%)	4,109 (73%)	
Rural	1,996 (18%)	1,009 (18%)	987 (18%)	
Missing	*1,082 (9.6%)*	*542 (9.6%)*	*540 (9.6%)*	
COVID-19 Vaccination Status				0.05 (0.01, 0.09)
No Documented COVID-19 Vaccination	6,852 (61%)	3,360 (60%)	3,492 (62%)	
Primary COVID-19 Vaccination Documented	2,065 (18%)	1,067 (19%)	998 (18%)	
Primary and Additional COVID-19 Dose(s) Documented	2,355 (21%)	1,209 (21%)	1,146 (20%)	
SARS-CoV-2 Variant-Dominant Period				0.00 (-0.04, 0.04)
Delta (B.1.617.2)	1,956 (17%)	978 (17%)	978 (17%)	
Omicron (B.1.1.529, BA.2, BA.2.12.1)	5,388 (48%)	2,694 (48%)	2,694 (48%)	
Omicron (BA.5, BQ.1.1, XBB.1.5)	3,928 (35%)	1,964 (35%)	1,964 (35%)	
Heart Failure Before COVID-19	1,088 (9.7%)	517 (9.2%)	571 (10%)	-0.03 (-0.07, 0.00)
Cardiovascular Disease Before COVID-19	4,497 (40%)	2,107 (37%)	2,390 (42%)	-0.10 (-0.14, -0.07)
Hypertension Before COVID-19	6,402 (57%)	3,144 (56%)	3,258 (58%)	-0.04 (-0.08, 0.00)
Obesity Before COVID-19	6,802 (60%)	3,336 (59%)	3,466 (61%)	-0.05 (-0.08, -0.01)
Tobacco Usage Before COVID-19	2,111 (19%)	1,077 (19%)	1,034 (18%)	0.02 (-0.02, 0.06)
Diabetes Before COVID-19	3,017 (27%)	1,535 (27%)	1,482 (26%)	0.02 (-0.02, 0.06)
Baseline eGFR Before COVID-19	91 (79, 103)	92 (79, 103)	91 (78, 102)	0.04 (0.00, 0.08)
Post-COVID-19 Earliest eGFR	91 (79, 103)	91 (78, 103)	92 (79, 103)	0.01 (-0.03, 0.05)
Post-COVID-19 Latest eGFR	90 (76, 102)	90 (77, 102)	90 (76, 101)	0.05 (0.02, 0.09)
Post-COVID-19 Average eGFR	90 (79, 101)	90 (79, 101)	90 (78, 100)	0.03 (0.00, 0.07)
Change in eGFR Post-COVID	-1 (-8, 5)	-1 (-7, 5)	-1 (-8, 5)	0.06 (0.03, 0.10)
Adverse post-COVID-19 Events				
eGFR Decline - 28+ Days after Acute COVID-19 (90+ Days Apart)	895 (7.9%)	412 (7.3%)	483 (8.6%)	-0.05 (-0.08, -0.01)
Death - 28+ Days After Acute COVID-19 (With two Creatinine Measures 90+ Days Apart, No eGFR Decline)	109 (1.0%)	57 (1.0%)	52 (0.9%)	0.01 (-0.03, 0.05)
Any Death After COVID-19	135 (1.2%)	68 (1.2%)	67 (1.2%)	0.00 (-0.04, 0.04)

1. n (%); Median (IQR)
2. Standardized mean differences (SMD) with 95% confidence intervals (CI)

Supplemental Table 17 provides descriptive statistics for patients with a documented SARS-CoV-2 infection between October 1, 2021, and September 30, 2023, among patients with at least 2 eGFR measurements for eGFR-qualifying CKD (sensitivity analysis 1) after propensity-score matching (PSM). This includes all patients seen at clinics with >=250 long COVID cases based on study inclusion/exclusion criteria. All patients in this cohort must have at least 2 eGFR measurements (at least 90 days apart) documented 30 days after initial SARS-CoV-2 infection. Abbreviations: Coronavirus disease 2019 (COVID-19), acute respiratory syndrome coronavirus (SARS-CoV-2), chronic kidney disease (CKD), end-stage kidney disease (ESKD), acute kidney injury (AKI).

Supplemental Table 18. Univariate and Multivariable Cox Proportional Hazards Regression for eGFR Decline After PSM (Sensitivity Analysis 1)

Characteristic	Unadjusted HR (95% CI)	*p* value	Adjusted sHR (95% CI)	*p* value
COVID-19 Status				
Acute COVID-19	Reference		Reference	
Long COVID	1.18 (1.03, 1.34)	0.02	1.15 (1.01, 1.31)	0.04
Sex				
Female	Reference		Reference	
Male	1.33 (1.16, 1.53)	<0.001	1.27 (1.11, 1.47)	<0.001
Age Group				
60-69	Reference		Reference	
18-29	0.60 (0.41, 0.89)	0.011	0.66 (0.44, 0.98)	0.04
30-39	0.72 (0.56, 0.93)	0.011	0.76 (0.59, 0.98)	0.04
40-49	0.85 (0.70, 1.05)	0.13	0.90 (0.73, 1.12)	0.35
50-59	0.82 (0.68, 0.99)	0.04	0.83 (0.69, 1.00)	0.05
70-79	0.88 (0.72, 1.07)	0.20	0.89 (0.73, 1.09)	0.28
80+	0.94 (0.69, 1.27)	0.67	0.94 (0.69, 1.28)	0.68
Race/Ethnicity				
Black or African American Non-Hispanic	1.20 (0.98, 1.47)	0.08	1.27 (1.03, 1.57)	0.03
Hispanic or Latino Any Race	0.78 (0.60, 1.01)	0.06	0.82 (0.63, 1.07)	0.15
Other non-Hispanic	0.81 (0.56, 1.17)	0.27	0.91 (0.63, 1.31)	0.60
Unknown	0.69 (0.44, 1.09)	0.11	0.77 (0.49, 1.21)	0.26
White non-Hispanic	Reference		Reference	
SARS-CoV-2 Variant Period				
Omicron (B.1.1.529, BA.2, BA.2.12.1)	Reference		Reference	
Delta (B.1.617.2)	1.54 (1.31, 1.82)	<0.001	1.43 (1.21, 1.69)	<0.001
Omicron (BA.5, BQ.1.1, XBB.1.5)	1.03 (0.89, 1.20)	0.67	1.04 (0.89, 1.21)	0.65
COVID-19 Vaccination Status				
No Documented COVID-19 Vaccination	Reference		Reference	
Primary COVID-19 Vaccination Documented	0.89 (0.75, 1.07)	0.21	0.89 (0.75, 1.06)	0.20
Primary and Additional COVID-19 Dose(s) Documented	0.77 (0.64, 0.91)	0.003	0.81 (0.67, 0.98)	0.03
Heart Failure				
No History of HF	Reference		Reference	
History of HF	1.76 (1.47, 2.11)	<0.001	1.41 (1.15, 1.73)	<0.001
Diabetes				
No History of Diabetes	Reference		Reference	
History of Diabetes	1.17 (1.02, 1.35)	0.03	1.05 (0.90, 1.22)	0.54
Hypertension				
No History of HTN	Reference		Reference	
History of HTN	1.13 (0.98, 1.29)	0.08	0.87 (0.74, 1.01)	0.07
Cardiovascular Disease				
No History of CVD	Reference		Reference	
History of CVD	1.47 (1.29, 1.67)	<0.001	1.29 (1.11, 1.50)	0.001
Obesity				
No History of Obesity	Reference		Reference	
History of Obesity	1.25 (1.09, 1.43)	0.002	1.20 (1.04, 1.39)	0.01
Tobacco Usage				
No History of Tobacco Usage	Reference		Reference	
History of Tobacco Usage	1.13 (0.97, 1.33)	0.13	1.05 (0.89, 1.24)	0.54
Rurality				
Urban	Reference		Reference	
Rural	1.27 (1.08, 1.49)	0.005	1.17 (0.99, 1.38)	0.07
Missing	1.12 (0.90, 1.40)	0.30	1.11 (0.89, 1.39)	0.36

Supplemental Table 18 includes univariate and multivariable Cox Proportional Hazards for eGFR decline among patients with a SARS-CoV-2 infection documented between October 1, 2021, and September 30, 2023, among patients with at least 2 eGFR measurements for eGFR-qualifying CKD (sensitivity analysis 1) after propensity-score matching (PSM). eGFR decline is assessed using the earliest post-COVID-19 eGFR measurement after infection (>= 30 days) and the last post-COVID-19 eGFR measurement taken within 365 days. These measurements must be at least 90 days apart. A decrease of >=20% is used as the threshold for eGFR decline. Abbreviations: hazard ratio (HR), subdistribution hazard ratio (sHR), Coronavirus disease 2019 (COVID-19), acute respiratory syndrome coronavirus (SARS-CoV-2), chronic kidney disease (CKD), end-stage kidney disease (ESKD), acute kidney injury (AKI).

Supplemental Table 19. Univariate and Multivariable Cox Proportional Hazards Regression for *Long COVID* Including Adjustment for Acute COVID-19 Severity After PSM (Sensitivity Analysis 2)

Characteristic	Unadjusted cHR (95% CI)	*p* value	Adjusted sHR (95% CI)	*p* value
CKD/ESKD Status				
No baseline CKD/ESKD	Reference		Reference	
Baseline CKD/ESKD	1.31 (1.26, 1.36)	<0.001	1.08 (1.03, 1.12)	<0.001
Sex				
Female	Reference		Reference	
Male	0.80 (0.77, 0.83)	<0.001	0.75 (0.72, 0.78)	<0.001
Age Group				
60-69	Reference		Reference	
18-29	0.51 (0.40, 0.64)	<0.001	0.55 (0.43, 0.69)	<0.001
30-39	0.74 (0.65, 0.84)	<0.001	0.78 (0.69, 0.89)	<0.001
40-49	0.98 (0.91, 1.07)	0.68	1.01 (0.93, 1.10)	0.79
50-59	0.99 (0.93, 1.05)	0.74	1.01 (0.95, 1.07)	0.84
70-79	0.90 (0.86, 0.94)	<0.001	0.89 (0.85, 0.94)	<0.001
80+	0.72 (0.68, 0.77)	<0.001	0.69 (0.65, 0.73)	<0.001
Race/Ethnicity				
Black or African American Non-Hispanic	0.89 (0.84, 0.94)	<0.001	0.77 (0.73, 0.81)	<0.001
Hispanic or Latino Any Race	1.02 (0.95, 1.11)	0.56	0.96 (0.89, 1.04)	0.29
Other non-Hispanic	0.80 (0.73, 0.88)	<0.001	0.89 (0.81, 0.98)	0.01
Unknown	0.89 (0.79, 1.01)	0.08	0.95 (0.84, 1.08)	0.47
White non-Hispanic	Reference		Reference	
SARS-CoV-2 Variant Period				
Omicron (B.1.1.529, BA.2, BA.2.12.1)	Reference		Reference	
Delta (B.1.617.2)	1.54 (1.46, 1.62)	<0.001	1.43 (1.36, 1.51)	<0.001
Omicron (BA.5, BQ.1.1, XBB.1.5)	0.70 (0.67, 0.72)	<0.001	0.69 (0.66, 0.72)	<0.001
COVID-19 Vaccination Status				
No Documented COVID-19 Vaccination	Reference		Reference	
Primary COVID-19 Vaccination Documented	0.87 (0.82, 0.91)	<0.001	0.85 (0.81, 0.90)	<0.001
Primary and Additional COVID-19 Dose(s) Documented	0.75 (0.72, 0.79)	<0.001	0.92 (0.88, 0.97)	0.001
Heart Failure				
No History of HF	Reference		Reference	
History of HF	1.31 (1.26, 1.37)	<0.001	0.91 (0.86, 0.96)	<0.001
Diabetes				
No History of Diabetes	Reference		Reference	
History of Diabetes	1.21 (1.16, 1.26)	<0.001	0.94 (0.90, 0.98)	0.004
Hypertension				
No History of HTN	Reference		Reference	
History of HTN	1.35 (1.29, 1.40)	<0.001	1.15 (1.10, 1.21)	<0.001
Cardiovascular Disease				
No History of CVD	Reference		Reference	
History of CVD	1.37 (1.33, 1.43)	<0.001	1.24 (1.18, 1.30)	<0.001
Obesity				
No History of Obesity	Reference		Reference	
History of Obesity	1.52 (1.47, 1.58)	<0.001	1.36 (1.30, 1.42)	<0.001
Tobacco Usage				
No History of Tobacco Usage	Reference		Reference	
History of Tobacco Usage	1.21 (1.15, 1.27)	<0.001	1.05 (1.00, 1.10)	0.06
Rurality				
Urban	Reference		Reference	
Rural	1.08 (1.03, 1.13)	0.003	1.00 (0.95, 1.05)	>0.99
Missing	1.01 (0.95, 1.07)	0.74	0.98 (0.92, 1.04)	0.53
Acute COVID-19 Severity				
Mild: Non-Hospitalized	Reference		Reference	
Moderate: Hospitalized	1.80 (1.71, 1.90)	<0.001	1.82 (1.72, 1.92)	<0.001
Severe: Supplemental Oxygen in Hospital	3.58 (3.39, 3.78)	<0.001	3.33 (3.13, 3.55)	<0.001

Supplemental Table 19 includes univariate and multivariable Cox Proportional Hazards for long COVID (U09.9), including adjustments for acute COVID-19 severity (sensitivity analysis 2) after propensity score matching (PSM). Severity is classified into non-hospitalized, requiring hospitalization around acute COVID-19, and requiring oxygen support or mechanical ventilation while admitted. Patients were PSM using logistic regression with 1:1 matching for binary long COVID status with exact matching on sex, race/ethnicity, COVID-19 variant period, and data-contributing site and nearest neighbor PSM on age. Abbreviations: hazard ratio (HR), subdistribution hazard ratio (sHR), Coronavirus disease 2019 (COVID-19), acute respiratory syndrome coronavirus (SARS-CoV-2), chronic kidney disease (CKD), end-stage kidney disease (ESKD), acute kidney injury (AKI).

Supplemental Table 20. Univariate and Multivariable Cox Proportional Hazards Regression for *Incident CKD/ESKD* Including Adjustment for Acute COVID-19 Severity After PSM (Sensitivity Analysis 2)

Characteristic	Unadjusted HR (95% CI)	*p* value	Adjusted sHR (95% CI)	*p* value
COVID-19 Status				
Acute COVID-19	Reference		Reference	
Long COVID	1.73 (1.58, 1.89)	<0.001	1.51 (1.37, 1.65)	<0.001
Sex				
Female	Reference		Reference	
Male	1.36 (1.25, 1.49)	<0.001	1.02 (0.93, 1.12)	0.67
Age Group				
60-69	Reference		Reference	
18-29	0.09 (0.06, 0.13)	<0.001	0.13 (0.09, 0.20)	<0.001
30-39	0.13 (0.10, 0.17)	<0.001	0.17 (0.13, 0.22)	<0.001
40-49	0.27 (0.23, 0.32)	<0.001	0.33 (0.27, 0.39)	<0.001
50-59	0.57 (0.50, 0.64)	<0.001	0.62 (0.55, 0.71)	<0.001
70-79	1.49 (1.33, 1.68)	<0.001	1.45 (1.28, 1.64)	<0.001
80+	2.40 (2.06, 2.78)	<0.001	2.12 (1.81, 2.49)	<0.001
Race/Ethnicity				
Black or African American Non-Hispanic	1.14 (1.00, 1.31)	0.06	1.37 (1.18, 1.58)	<0.001
Hispanic or Latino Any Race	0.50 (0.42, 0.61)	<0.001	0.69 (0.57, 0.84)	<0.001
Other non-Hispanic	0.56 (0.44, 0.71)	<0.001	0.77 (0.60, 0.98)	0.03
Unknown	0.44 (0.32, 0.59)	<0.001	0.65 (0.48, 0.88)	0.006
White non-Hispanic	Reference		Reference	
SARS-CoV-2 Variant Period				
Omicron (B.1.1.529, BA.2, BA.2.12.1)	Reference		Reference	
Delta (B.1.617.2)	1.23 (1.10, 1.38)	<0.001	1.05 (0.93, 1.19)	0.38
Omicron (BA.5, BQ.1.1, XBB.1.5)	1.15 (1.05, 1.28)	0.005	1.00 (0.90, 1.11)	>0.99
COVID-19 Vaccination Status				
No Documented COVID-19 Vaccination	Reference		Reference	
Primary COVID-19 Vaccination Documented	0.93 (0.83, 1.05)	0.24	0.96 (0.85, 1.08)	0.49
Primary and Additional COVID-19 Dose(s) Documented	0.97 (0.86, 1.09)	0.62	0.84 (0.74, 0.95)	0.006
Heart Failure				
No History of HF	Reference		Reference	
History of HF	3.75 (3.30, 4.27)	<0.001	1.41 (1.21, 1.64)	<0.001
Diabetes				
No History of Diabetes	Reference		Reference	
History of Diabetes	2.41 (2.19, 2.65)	<0.001	1.40 (1.26, 1.56)	<0.001
Hypertension				
No History of HTN	Reference		Reference	
History of HTN	2.83 (2.59, 3.09)	<0.001	1.25 (1.13, 1.39)	<0.001
Cardiovascular Disease				
No History of CVD	Reference		Reference	
History of CVD	2.55 (2.34, 2.79)	<0.001	1.16 (1.04, 1.30)	0.006
Obesity				
No History of Obesity	Reference		Reference	
History of Obesity	1.44 (1.32, 1.57)	<0.001	1.22 (1.11, 1.35)	<0.001
Tobacco Usage				
No History of Tobacco Usage	Reference		Reference	
History of Tobacco Usage	1.11 (0.99, 1.25)	0.08	1.03 (0.91, 1.16)	0.64
Rurality				
Urban	Reference		Reference	
Rural	1.47 (1.32, 1.65)	<0.001	1.25 (1.11, 1.39)	<0.001
Missing	1.06 (0.92, 1.23)	0.40	1.07 (0.93, 1.24)	0.34
Acute COVID-19 Severity				
Mild: Non-Hospitalized	Reference		Reference	
Moderate: Hospitalized	2.95 (2.59, 3.35)	<0.001	1.73 (1.51, 1.99)	<0.001
Severe: Supplemental Oxygen in Hospital	3.82 (3.34, 4.37)	<0.001	1.94 (1.67, 2.26)	<0.001

Supplemental Table 20 includes univariate and multivariable Cox Proportional Hazards for incident CKD/ESKD among patients with a SARS-CoV-2 infection documented between October 1, 2021, and September 30, 2023, including adjustments for acute COVID-19 severity (sensitivity analysis 2) after propensity score matching (PSM). Severity is classified into non-hospitalized, requiring hospitalization around acute COVID-19, and requiring oxygen support or mechanical ventilation while admitted. Patients were PSM using logistic regression with 1:1 matching for binary long COVID status with exact matching on sex, race/ethnicity, COVID-19 variant period, and data-contributing site and nearest neighbor PSM on age. Abbreviations: hazard ratio (HR), subdistribution hazard ratio (sHR), Coronavirus disease 2019 (COVID-19), acute respiratory syndrome coronavirus (SARS-CoV-2), chronic kidney disease (CKD), end-stage kidney disease (ESKD), acute kidney injury (AKI).

Supplemental Table 21. Univariate and Multivariable Cox Proportional Hazards Regression for *eGFR Decline* Including Adjustment for Acute COVID-19 Severity After PSM (Sensitivity Analysis 2)

Characteristic	Unadjusted HR (95% CI)	*p* value	Adjusted sHR (95% CI)	*p* value
COVID-19 Status				
Acute COVID-19	Reference		Reference	
Long COVID	1.24 (1.07, 1.44)	0.004	1.13 (0.97, 1.31)	0.11
Sex				
Female	Reference		Reference	
Male	1.27 (1.09, 1.48)	0.002	1.13 (0.97, 1.33)	0.12
Age Group				
60-69	Reference		Reference	
18-29	0.64 (0.42, 0.97)	0.04	0.69 (0.45, 1.07)	0.09
30-39	0.71 (0.54, 0.94)	0.02	0.76 (0.57, 1.01)	0.06
40-49	0.91 (0.73, 1.14)	0.42	0.98 (0.77, 1.24)	0.85
50-59	0.92 (0.75, 1.14)	0.46	0.96 (0.77, 1.18)	0.67
70-79	0.99 (0.79, 1.25)	0.96	1.01 (0.80, 1.27)	0.94
80+	0.88 (0.60, 1.29)	0.52	0.84 (0.57, 1.25)	0.38
Race/Ethnicity				
Black or African American Non-Hispanic	1.19 (0.95, 1.50)	0.13	1.23 (0.97, 1.56)	0.09
Hispanic or Latino Any Race	0.77 (0.58, 1.03)	0.08	0.83 (0.62, 1.11)	0.21
Other non-Hispanic	0.89 (0.60, 1.30)	0.54	1.00 (0.68, 1.48)	>0.99
Unknown	0.75 (0.46, 1.21)	0.24	0.90 (0.55, 1.46)	0.66
White non-Hispanic	Reference		Reference	
SARS-CoV-2 Variant Period				
Omicron (B.1.1.529, BA.2, BA.2.12.1)	Reference		Reference	
Delta (B.1.617.2)	1.69 (1.41, 2.03)	<0.001	1.44 (1.19, 1.74)	<0.001
Omicron (BA.5, BQ.1.1, XBB.1.5)	1.01 (0.85, 1.20)	0.89	1.04 (0.87, 1.25)	0.66
COVID-19 Vaccination Status				
No Documented COVID-19 Vaccination	Reference		Reference	
Primary COVID-19 Vaccination Documented	1.06 (0.88, 1.27)	0.56	1.11 (0.92, 1.34)	0.27
Primary and Additional COVID-19 Dose(s) Documented	0.78 (0.63, 0.95)	0.01	0.92 (0.74, 1.14)	0.43
Heart Failure				
No History of HF	Reference		Reference	
History of HF	1.65 (1.33, 2.06)	<0.001	1.34 (1.04, 1.72)	0.02
Diabetes				
No History of Diabetes	Reference		Reference	
History of Diabetes	1.15 (0.98, 1.35)	0.09	1.02 (0.86, 1.21)	0.80
Hypertension				
No History of HTN	Reference		Reference	
History of HTN	1.09 (0.94, 1.26)	0.27	0.88 (0.74, 1.04)	0.12
Cardiovascular Disease				
No History of CVD	Reference		Reference	
History of CVD	1.24 (1.07, 1.44)	0.004	1.06 (0.89, 1.26)	0.51
Obesity				
No History of Obesity	Reference		Reference	
History of Obesity	1.21 (1.04, 1.41)	0.01	1.14 (0.97, 1.35)	0.11
Tobacco Usage				
No History of Tobacco Usage	Reference		Reference	
History of Tobacco Usage	1.18 (0.99, 1.42)	0.07	1.11 (0.92, 1.33)	0.28
Rurality				
Urban	Reference		Reference	
Rural	1.27 (1.06, 1.52)	0.01	1.15 (0.95, 1.38)	0.15
Missing	1.00 (0.77, 1.30)	0.99	1.00 (0.77, 1.30)	0.99
Acute COVID-19 Severity				
Mild: Non-Hospitalized	Reference		Reference	
Moderate: Hospitalized	2.54 (2.08, 3.10)	<0.001	2.25 (1.83, 2.76)	<0.001
Severe: Supplemental Oxygen in Hospital	2.64 (2.06, 3.38)	<0.001	2.14 (1.65, 2.79)	<0.001

Supplemental Table 21 includes univariate and multivariable Cox Proportional Hazards for eGFR decline among patients with a SARS-CoV-2 infection documented between October 1, 2021, and September 30, 2023 including adjustments for acute COVID-19 severity (sensitivity analysis 2) after propensity score matching (PSM). Severity is classified into non-hospitalized, requiring hospitalization around acute COVID-19, and requiring oxygen support or mechanical ventilation while admitted. Patients were PSM using logistic regression with 1:1 matching for binary long COVID status with exact matching on sex, race/ethnicity, COVID-19 variant period, and data-contributing site and nearest neighbor PSM on age. eGFR decline is assessed using the earliest post-COVID-19 eGFR measurement after infection (>= 30 days) and the last post-COVID-19 eGFR measurement taken within 365 days. These measurements must be at least 90 days apart. A decrease of >=20% is used as the threshold for eGFR decline. Abbreviations: hazard ratio (HR), subdistribution hazard ratio (sHR), Coronavirus disease 2019 (COVID-19), acute respiratory syndrome coronavirus (SARS-CoV-2), chronic kidney disease (CKD), end-stage kidney disease (ESKD), acute kidney injury (AKI).

Supplemental Table 22. Univariate and Multivariable Cox Proportional Hazards Regression for *Long COVID* Among Patients *Under 65* After PSM (Sensitivity Analysis 3)

Characteristic	Unadjusted HR (95% CI)	*p* value	Adjusted sHR (95% CI)	*p* value
CKD/ESKD Status				
No baseline CKD/ESKD	Reference		Reference	
Baseline CKD/ESKD	1.31 (1.23, 1.39)	<0.001	1.08 (1.01, 1.16)	0.02
Sex				
Female	Reference		Reference	
Male	0.68 (0.64, 0.73)	<0.001	0.65 (0.61, 0.70)	<0.001
Age Group				
40-49	Reference		Reference	
18-29	0.49 (0.38, 0.63)	<0.001	0.52 (0.40, 0.66)	<0.001
30-39	0.80 (0.69, 0.92)	0.002	0.82 (0.72, 0.95)	0.007
50-59	1.06 (0.97, 1.15)	0.21	1.05 (0.96, 1.15)	0.28
60-69	1.07 (0.98, 1.17)	0.15	1.06 (0.96, 1.16)	0.25
Race/Ethnicity				
Black or African American Non-Hispanic	0.83 (0.77, 0.90)	<0.001	0.81 (0.75, 0.88)	<0.001
Hispanic or Latino Any Race	0.95 (0.85, 1.07)	0.40	1.03 (0.92, 1.15)	0.65
Other non-Hispanic	0.85 (0.74, 0.98)	0.03	0.97 (0.84, 1.13)	0.72
Unknown	0.92 (0.76, 1.12)	0.42	1.05 (0.86, 1.27)	0.64
White non-Hispanic	Reference		Reference	
SARS-CoV-2 Variant Period				
Omicron (B.1.1.529, BA.2, BA.2.12.1)	Reference		Reference	
Delta (B.1.617.2)	1.63 (1.50, 1.77)	<0.001	1.62 (1.49, 1.76)	<0.001
Omicron (BA.5, BQ.1.1, XBB.1.5)	0.70 (0.66, 0.76)	<0.001	0.67 (0.63, 0.72)	<0.001
COVID-19 Vaccination Status				
No Documented COVID-19 Vaccination	Reference		Reference	
Primary COVID-19 Vaccination Documented	0.80 (0.73, 0.87)	<0.001	0.75 (0.69, 0.82)	<0.001
Primary and Additional COVID-19 Dose(s) Documented	0.80 (0.74, 0.87)	<0.001	0.90 (0.82, 0.98)	0.01
Heart Failure				
No History of HF	Reference		Reference	
History of HF	1.41 (1.29, 1.53)	<0.001	1.02 (0.92, 1.12)	0.71
Diabetes				
No History of Diabetes	Reference		Reference	
History of Diabetes	1.21 (1.13, 1.29)	<0.001	0.95 (0.88, 1.02)	0.13
Hypertension				
No History of HTN	Reference		Reference	
History of HTN	1.40 (1.32, 1.49)	<0.001	1.16 (1.07, 1.25)	<0.001
Cardiovascular Disease				
No History of CVD	Reference		Reference	
History of CVD	1.53 (1.44, 1.63)	<0.001	1.39 (1.29, 1.50)	<0.001
Obesity				
No History of Obesity	Reference		Reference	
History of Obesity	1.56 (1.46, 1.66)	<0.001	1.42 (1.32, 1.52)	<0.001
Tobacco Usage				
No History of Tobacco Usage	Reference		Reference	
History of Tobacco Usage	1.06 (0.98, 1.14)	0.14	1.00 (0.92, 1.08)	0.97
Rurality				
Urban	Reference		Reference	
Rural	1.15 (1.06, 1.25)	0.001	1.03 (0.95, 1.13)	0.43
Missing	1.06 (0.96, 1.17)	0.24	1.03 (0.94, 1.14)	0.50

Supplemental Table 22 includes univariate and multivariable Cox Proportional Hazards for incident CKD/ESKD among patients with a SARS-CoV-2 infection documented between October 1, 2021, and September 30, 2023, among patients under 65 (sensitivity analysis 3) after propensity score matching (PSM). Severity is classified into non-hospitalized, requiring hospitalization around acute COVID-19, and requiring oxygen support or mechanical ventilation while admitted. Patients were PSM using logistic regression with 1:1 matching for binary long COVID status with exact matching on sex, race/ethnicity, COVID-19 variant period, and data-contributing site and nearest neighbor PSM on age. Abbreviations: hazard ratio (HR), subdistribution hazard ratio (sHR), Coronavirus disease 2019 (COVID-19), acute respiratory syndrome coronavirus (SARS-CoV-2), chronic kidney disease (CKD), end-stage kidney disease (ESKD), acute kidney injury (AKI).

Supplemental Table 23. Univariatee and Multivariable Cox Proportional Hazards Regression for *Long COVID* Among Patients *65 and Older* After PSM (Sensitivity Analysis 3)

Characteristic	Unadjusted HR (95% CI)	*p* value	Adjusted sHR (95% CI)	*p* value
CKD/ESKD Status				
No baseline CKD/ESKD	Reference		Reference	
Baseline CKD/ESKD	1.28 (1.22, 1.34)	<0.001	1.13 (1.07, 1.19)	<0.001
Sex				
Female	Reference		Reference	
Male	0.87 (0.83, 0.92)	<0.001	0.83 (0.79, 0.87)	<0.001
Age Group				
70-79	Reference		Reference	
60-69	1.09 (1.03, 1.15)	0.005	1.08 (1.01, 1.14)	0.02
80+	0.81 (0.76, 0.85)	<0.001	0.82 (0.77, 0.86)	<0.001
Race/Ethnicity				
Black or African American Non-Hispanic	0.84 (0.77, 0.91)	<0.001	0.78 (0.72, 0.85)	<0.001
Hispanic or Latino Any Race	1.01 (0.90, 1.12)	0.90	0.98 (0.88, 1.10)	0.78
Other non-Hispanic	0.79 (0.70, 0.89)	<0.001	0.86 (0.76, 0.97)	0.02
Unknown	0.96 (0.82, 1.12)	0.58	1.02 (0.87, 1.20)	0.76
White non-Hispanic	Reference		Reference	
SARS-CoV-2 Variant Period				
Omicron (B.1.1.529, BA.2, BA.2.12.1)	Reference		Reference	
Delta (B.1.617.2)	1.54 (1.44, 1.66)	<0.001	1.51 (1.40, 1.62)	<0.001
Omicron (BA.5, BQ.1.1, XBB.1.5)	0.70 (0.66, 0.73)	<0.001	0.70 (0.66, 0.74)	<0.001
COVID-19 Vaccination Status				
No Documented COVID-19 Vaccination	Reference		Reference	
Primary COVID-19 Vaccination Documented	0.93 (0.87, 0.99)	0.02	0.84 (0.78, 0.89)	<0.001
Primary and Additional COVID-19 Dose(s) Documented	0.76 (0.72, 0.81)	<0.001	0.84 (0.79, 0.89)	<0.001
Heart Failure				
No History of HF	Reference		Reference	
History of HF	1.33 (1.26, 1.40)	<0.001	1.09 (1.02, 1.16)	0.008
Diabetes				
No History of Diabetes	Reference		Reference	
History of Diabetes	1.17 (1.12, 1.23)	<0.001	0.97 (0.92, 1.02)	0.22
Hypertension				
No History of HTN	Reference		Reference	
History of HTN	1.35 (1.28, 1.43)	<0.001	1.16 (1.09, 1.23)	<0.001
Cardiovascular Disease				
No History of CVD	Reference		Reference	
History of CVD	1.36 (1.30, 1.43)	<0.001	1.24 (1.17, 1.31)	<0.001
Obesity				
No History of Obesity	Reference		Reference	
History of Obesity	1.51 (1.44, 1.58)	<0.001	1.34 (1.28, 1.41)	<0.001
Tobacco Usage				
No History of Tobacco Usage	Reference		Reference	
History of Tobacco Usage	1.23 (1.15, 1.31)	<0.001	1.15 (1.08, 1.23)	<0.001
Rurality				
Urban	Reference		Reference	
Rural	1.10 (1.04, 1.17)	0.002	1.05 (0.99, 1.12)	0.11
Missing	0.94 (0.87, 1.02)	0.15	0.90 (0.83, 0.98)	0.01

Supplemental Table 23 includes univariate and multivariable Cox Proportional Hazards for incident CKD/ESKD among patients with a SARS-CoV-2 infection documented between October 1, 2021, and September 30, 2023, among patients under 65 (sensitivity analysis 3) after propensity score matching (PSM). Severity is classified into non-hospitalized, requiring hospitalization around acute COVID-19, and requiring oxygen support or mechanical ventilation while admitted. Patients were PSM using logistic regression with 1:1 matching for binary long COVID status with exact matching on sex, race/ethnicity, COVID-19 variant period, and data-contributing site and nearest neighbor PSM on age. Abbreviations: hazard ratio (HR), subdistribution hazard ratio (sHR), Coronavirus disease 2019 (COVID-19), acute respiratory syndrome coronavirus (SARS-CoV-2), chronic kidney disease (CKD), end-stage kidney disease (ESKD), acute kidney injury (AKI).

Supplemental Table 24. Univariate and Multivariable Cox Proportional Hazards Regression for *Incident CKD/ESKD* Among Patients *Under 65* After PSM (Sensitivity Analysis 3)

Characteristic	Unadjusted HR (95% CI)	*p* value	Adjusted sHR (95% CI)	*p* value
COVID-19 Status				
Acute COVID-19	Reference		Reference	
Long COVID	1.83 (1.61, 2.09)	<0.001	1.74 (1.53, 1.98)	<0.001
Sex				
Female	Reference		Reference	
Male	1.37 (1.21, 1.56)	<0.001	1.15 (1.01, 1.32)	0.04
Age Group				
40-49	Reference		Reference	
18-29	0.23 (0.14, 0.37)	<0.001	0.29 (0.18, 0.47)	<0.001
30-39	0.48 (0.37, 0.63)	<0.001	0.55 (0.42, 0.71)	<0.001
50-59	1.98 (1.68, 2.34)	<0.001	1.72 (1.45, 2.05)	<0.001
60-69	2.78 (2.32, 3.34)	<0.001	2.20 (1.82, 2.67)	<0.001
Race/Ethnicity				
Black or African American Non-Hispanic	1.52 (1.28, 1.80)	<0.001	1.48 (1.24, 1.77)	<0.001
Hispanic or Latino Any Race	0.59 (0.46, 0.75)	<0.001	0.70 (0.55, 0.90)	0.005
Other non-Hispanic	0.54 (0.39, 0.77)	<0.001	0.69 (0.49, 0.97)	0.033
Unknown	0.58 (0.40, 0.84)	0.004	0.74 (0.51, 1.07)	0.11
White non-Hispanic	Reference		Reference	
SARS-CoV-2 Variant Period				
Omicron (B.1.1.529, BA.2, BA.2.12.1)	Reference		Reference	
Delta (B.1.617.2)	1.37 (1.18, 1.60)	<0.001	1.20 (1.02, 1.40)	0.03
Omicron (BA.5, BQ.1.1, XBB.1.5)	1.11 (0.96, 1.29)	0.14	1.10 (0.95, 1.27)	0.24
COVID-19 Vaccination Status				
No Documented COVID-19 Vaccination	Reference		Reference	
Primary COVID-19 Vaccination Documented	0.84 (0.71, 1.00)	0.05	0.87 (0.73, 1.04)	0.12
Primary and Additional COVID-19 Dose(s) Documented	0.76 (0.63, 0.92)	0.005	0.76 (0.63, 0.93)	0.007
Heart Failure				
No History of HF	Reference		Reference	
History of HF	4.61 (3.76, 5.65)	<0.001	1.77 (1.40, 2.23)	<0.001
Diabetes				
No History of Diabetes	Reference		Reference	
History of Diabetes	2.91 (2.54, 3.33)	<0.001	1.64 (1.41, 1.91)	<0.001
Hypertension				
No History of HTN	Reference		Reference	
History of HTN	2.93 (2.59, 3.32)	<0.001	1.40 (1.21, 1.63)	<0.001
Cardiovascular Disease				
No History of CVD	Reference		Reference	
History of CVD	2.57 (2.25, 2.92)	<0.001	1.33 (1.14, 1.56)	<0.001
Obesity				
No History of Obesity	Reference		Reference	
History of Obesity	1.74 (1.53, 1.97)	<0.001	1.11 (0.97, 1.28)	0.14
Tobacco Usage				
No History of Tobacco Usage	Reference		Reference	
History of Tobacco Usage	1.53 (1.32, 1.77)	<0.001	1.25 (1.07, 1.46)	0.004
Rurality				
Urban	Reference		Reference	
Rural	1.43 (1.22, 1.67)	<0.001	1.23 (1.04, 1.45)	0.02
Missing	0.98 (0.80, 1.20)	0.84	0.95 (0.78, 1.17)	0.65

Supplemental Table 24 includes univariate and multivariable Cox Proportional Hazards for incident CKD/ESKD among patients with a SARS-CoV-2 infection documented between October 1, 2021, and September 30, 2023, among patients under 65 (sensitivity analysis 3) after propensity score matching (PSM). Severity is classified into non-hospitalized, requiring hospitalization around acute COVID-19, and requiring oxygen support or mechanical ventilation while admitted. Patients were PSM using logistic regression with 1:1 matching for binary long COVID status with exact matching on sex, race/ethnicity, COVID-19 variant period, and data-contributing site and nearest neighbor PSM on age. Abbreviations: hazard ratio (HR), subdistribution hazard ratio (sHR), Coronavirus disease 2019 (COVID-19), acute respiratory syndrome coronavirus (SARS-CoV-2), chronic kidney disease (CKD), end-stage kidney disease (ESKD), acute kidney injury (AKI).

Supplemental Table 25. Univariate and Multivariable Cox Proportional Hazards Regression for *Incident CKD/ESKD* Among Patients *65 and Older* After PSM (Sensitivity Analysis 3)

Characteristic	Unadjusted HR (95% CI)	*p* value	Adjusted sHR (95% CI)	*p* value
COVID-19 Status				
Acute COVID-19	Reference		Reference	
Long COVID	1.57 (1.38, 1.77)	<0.001	1.53 (1.35, 1.74)	<0.001
Sex				
Female	Reference		Reference	
Male	1.05 (0.93, 1.19)	0.40	1.00 (0.88, 1.13)	0.96
Age Group				
70-79	Reference		Reference	
60-69	0.75 (0.65, 0.87)	<0.001	0.75 (0.65, 0.87)	<0.001
80+	1.57 (1.34, 1.84)	<0.001	1.53 (1.30, 1.79)	<0.001
Race/Ethnicity				
Black or African American Non-Hispanic	1.51 (1.20, 1.91)	<0.001	1.48 (1.17, 1.89)	0.001
Hispanic or Latino Any Race	0.85 (0.64, 1.13)	0.27	0.86 (0.64, 1.15)	0.30
Other non-Hispanic	0.73 (0.51, 1.04)	0.08	0.75 (0.52, 1.07)	0.11
Unknown	0.89 (0.60, 1.32)	0.55	0.93 (0.62, 1.38)	0.70
White non-Hispanic	Reference		Reference	
SARS-CoV-2 Variant Period				
Delta (B.1.617.2)	Reference		Reference	
Omicron (B.1.1.529, BA.2, BA.2.12.1)	0.98 (0.82, 1.17)	0.79	0.95 (0.79, 1.14)	0.58
Omicron (BA.5, BQ.1.1, XBB.1.5)	0.96 (0.84, 1.10)	0.54	0.95 (0.83, 1.09)	0.45
COVID-19 Vaccination Status				
No Documented COVID-19 Vaccination	Reference		Reference	
Primary COVID-19 Vaccination Documented	1.04 (0.87, 1.23)	0.67	1.01 (0.85, 1.20)	0.91
Primary and Additional COVID-19 Dose(s) Documented	0.94 (0.81, 1.09)	0.43	0.93 (0.79, 1.08)	0.34
Heart Failure				
No History of HF	Reference		Reference	
History of HF	1.89 (1.60, 2.22)	<0.001	1.44 (1.19, 1.73)	<0.001
Diabetes				
No History of Diabetes	Reference		Reference	
History of Diabetes	1.45 (1.26, 1.66)	<0.001	1.28 (1.10, 1.48)	0.001
Hypertension				
No History of HTN	Reference		Reference	
History of HTN	1.36 (1.20, 1.55)	<0.001	1.05 (0.91, 1.21)	0.54
Cardiovascular Disease				
No History of CVD	Reference		Reference	
History of CVD	1.48 (1.31, 1.67)	<0.001	1.15 (1.0, 1.32)	0.06
Obesity				
No History of Obesity	Reference		Reference	
History of Obesity	1.21 (1.07, 1.37)	0.002	1.10 (0.96, 1.25)	0.16
Tobacco Usage				
No History of Tobacco Usage	Reference		Reference	
History of Tobacco Usage	1.02 (0.85, 1.22)	0.86	0.99 (0.82, 1.19)	>0.99
Rurality				
Urban	Reference		Reference	
Rural	1.29 (1.10, 1.51)	0.001	1.28 (1.09, 1.50)	0.002
Missing	1.16 (0.95, 1.41)	0.14	1.14 (0.94, 1.39)	0.19

Supplemental Table 25 includes univariate and multivariable Cox Proportional Hazards for incident CKD/ESKD among patients with a SARS-CoV-2 infection documented between October 1, 2021, and September 30, 2023, among patients 65 and older (sensitivity analysis 3) after propensity score matching (PSM). Severity is classified into non-hospitalized, requiring hospitalization around acute COVID-19, and requiring oxygen support or mechanical ventilation while admitted. Patients were PSM using logistic regression with 1:1 matching for binary long COVID status with exact matching on sex, race/ethnicity, COVID-19 variant period, and data-contributing site and nearest neighbor PSM on age. Abbreviations: hazard ratio (HR), subdistribution hazard ratio (sHR), Coronavirus disease 2019 (COVID-19), acute respiratory syndrome coronavirus (SARS-CoV-2), chronic kidney disease (CKD), end-stage kidney disease (ESKD), acute kidney injury (AKI).

Supplemental Table 26. Univariate and Multivariable Cox Proportional Hazards Regression for *eGFR Decline* Among Patients *Under 65* After PSM (Sensitivity Analysis 3)

Characteristic	Unadjusted HR (95% CI)	*p* value	Adjusted sHR (95% CI)	*p* value
COVID-19 Status				
Acute COVID-19	Reference		Reference	
**Long COVID**	1.22 (1.02, 1.47)	0.03	1.20 (1.00, 1.44)	0.06
Sex				
Female	Reference		Reference	
Male	1.26 (1.03, 1.53)	0.02	1.24 (1.02, 1.52)	0.04
Age Group				
40-49	Reference		Reference	
18-29	0.62 (0.40, 0.96)	0.03	0.64 (0.41, 1.00)	0.05
30-39	0.80 (0.60, 1.07)	0.13	0.78 (0.58, 1.04)	0.10
50-59	0.86 (0.67, 1.09)	0.20	0.81 (0.63, 1.03)	0.09
60-69	1.04 (0.80, 1.35)	0.80	1.02 (0.78, 1.33)	0.91
Race/Ethnicity				
Black or African American Non-Hispanic	1.25 (0.96, 1.62)	0.10	1.25 (0.95, 1.65)	0.10
Hispanic or Latino Any Race	0.81 (0.58, 1.14)	0.22	0.85 (0.60, 1.19)	0.34
Other non-Hispanic	0.99 (0.63, 1.55)	0.95	1.10 (0.70, 1.73)	0.69
Unknown	0.61 (0.32, 1.14)	0.12	0.69 (0.36, 1.30)	0.25
White non-Hispanic	Reference		Reference	
SARS-CoV-2 Variant Period				
Omicron (B.1.1.529, BA.2, BA.2.12.1)	Reference		Reference	
Delta (B.1.617.2)	1.69 (1.36, 2.11)	<0.001	1.55 (1.24, 1.93)	<0.001
Omicron (BA.5, BQ.1.1, XBB.1.5)	0.93 (0.75, 1.17)	0.55	0.96 (0.77, 1.21)	0.76
COVID-19 Vaccination Status				
No Documented COVID-19 Vaccination	Reference		Reference	
Primary COVID-19 Vaccination Documented	0.96 (0.76, 1.21)	0.74	0.96 (0.76, 1.22)	0.75
Primary and Additional COVID-19 Dose(s) Documented	0.62 (0.46, 0.83)	0.001	0.69 (0.51, 0.93)	0.01
Heart Failure				
No History of HF	Reference		Reference	
History of HF	1.66 (1.22, 2.27)	0.001	1.36 (0.96, 1.93)	0.086
Diabetes				
No History of Diabetes	Reference		Reference	
History of Diabetes	1.15 (0.93, 1.41)	0.19	1.02 (0.82, 1.27)	0.86
Hypertension				
No History of HTN	Reference		Reference	
History of HTN	1.11 (0.93, 1.34)	0.24	0.89 (0.72, 1.09)	0.26
Cardiovascular Disease				
No History of CVD	Reference		Reference	
History of CVD	1.31 (1.09, 1.59)	0.005	1.19 (0.96, 1.48)	0.11
Obesity				
No History of Obesity	Reference		Reference	
History of Obesity	1.31 (1.08, 1.60)	0.007	1.26 (1.01, 1.55)	0.04
Tobacco Usage				
No History of Tobacco Usage	Reference		Reference	
History of Tobacco Usage	1.11 (0.89, 1.38)	0.35	1.04 (0.83, 1.29)	0.74
Rurality				
Urban	Reference		Reference	
Rural	1.27 (1.01, 1.59)	0.04	1.15 (0.91, 1.46)	0.23
Missing	1.00 (0.74, 1.37)	0.98	1.00 (0.73, 1.37)	>0.99

Supplemental Table 26 includes univariate and multivariable Cox Proportional Hazards for eGFR decline among patients with a SARS-CoV-2 infection documented between October 1, 2021, and September 30, 2023, among patients under 65 (sensitivity analysis 3) after propensity score matching (PSM). Severity is classified into non-hospitalized, requiring hospitalization around acute COVID-19, and requiring oxygen support or mechanical ventilation while admitted. Patients were PSM using logistic regression with 1:1 matching for binary long COVID status with exact matching on sex, race/ethnicity, COVID-19 variant period, and data-contributing site and nearest neighbor PSM on age. eGFR decline is assessed using the earliest post-COVID-19 eGFR measurement after infection (>= 30 days) and the last post-COVID-19 eGFR measurement taken within 365 days. These measurements must be at least 90 days apart. A decrease of >=20% is used as the threshold for eGFR decline. Abbreviations: hazard ratio (HR), subdistribution hazard ratio (sHR), Coronavirus disease 2019 (COVID-19), acute respiratory syndrome coronavirus (SARS-CoV-2), chronic kidney disease (CKD), end-stage kidney disease (ESKD), acute kidney injury (AKI).

Supplemental Table 27. Univariate and Multivariable Cox Proportional Hazards Regression for *eGFR Decline* Among Patients *65 and Older* After PSM (Sensitivity Analysis 3)

Characteristic	Unadjusted HR (95% CI)	*p* value	Adjusted sHR (95% CI)	*p* value
COVID-19 Status				
Acute COVID-19	Reference		Reference	
Long COVID	1.22 (1.05, 1.41)	0.008	1.35 (1.09, 1.67)	0.005
Sex				
Female	Reference		Reference	
Male	1.30 (1.12, 1.51)	<0.001	1.17 (0.94, 1.45)	0.17
Age Group				
70-79	Reference		Reference	
60-69	1.09 (0.87, 1.36)	0.46	1.08 (0.86, 1.36)	0.50
80+	0.79 (0.52, 1.20)	0.27	0.76 (0.50, 1.16)	0.20
Race/Ethnicity				
Black or African American Non-Hispanic	1.15 (0.91, 1.45)	0.24	1.16 (0.74, 1.82)	0.52
Hispanic or Latino Any Race	0.81 (0.61, 1.07)	0.14	1.33 (0.90, 1.99)	0.16
Other non-Hispanic	0.81 (0.55, 1.22)	0.32	1.10 (0.64, 1.90)	0.74
Unknown	0.88 (0.56, 1.38)	0.58	1.47 (0.80, 2.70)	0.21
White non-Hispanic	Reference		Reference	
SARS-CoV-2 Variant Period				
Delta (B.1.617.2)	Reference		Reference	
Omicron (B.1.1.529, BA.2, BA.2.12.1)	1.33 (1.10, 1.61)	0.003	1.31 (0.99, 1.73)	0.06
Omicron (BA.5, BQ.1.1, XBB.1.5)	0.96 (0.81, 1.14)	0.66	0.85 (0.67, 1.09)	0.21
COVID-19 Vaccination Status				
No Documented COVID-19 Vaccination	Reference		Reference	
Primary COVID-19 Vaccination Documented	0.95 (0.78, 1.15)	0.60	0.74 (0.55, 1.00)	0.05
Primary and Additional COVID-19 Dose(s) Documented	0.88 (0.73, 1.07)	0.20	0.86 (0.66, 1.13)	0.28
Heart Failure				
No History of HF	Reference		Reference	
History of HF	1.68 (1.36, 2.09)	<0.001	1.48 (1.10, 1.99)	0.01
Diabetes				
No History of Diabetes	Reference		Reference	
History of Diabetes	1.15 (0.98, 1.35)	0.09	1.05 (0.83, 1.33)	0.69
Hypertension				
No History of HTN	Reference		Reference	
History of HTN	1.18 (1.02, 1.37)	0.03	0.95 (0.74, 1.22)	0.67
Cardiovascular Disease				
No History of CVD	Reference		Reference	
History of CVD	1.38 (1.19, 1.60)	<0.001	1.34 (1.05, 1.70)	0.02
Obesity				
No History of Obesity	Reference		Reference	
History of Obesity	1.23 (1.05, 1.43)	0.009	1.07 (0.85, 1.35)	0.56
Tobacco Usage				
No History of Tobacco Usage	Reference		Reference	
History of Tobacco Usage	1.27 (1.07, 1.51)	0.007	1.08 (0.83, 1.42)	0.56
Rurality				
Urban	Reference		Reference	
Rural	1.16 (0.97, 1.40)	0.11	1.06 (0.81, 1.39)	0.65
Missing	0.98 (0.76, 1.27)	0.88	0.72 (0.46, 1.12)	0.14

Supplemental Table 27 includes univariate and multivariable Cox Proportional Hazards for eGFR decline among patients with a SARS-CoV-2 infection documented between October 1, 2021, and September 30, 2023, among patients 65 and older (sensitivity analysis 3) after propensity score matching (PSM). Severity is classified into non-hospitalized, requiring hospitalization around acute COVID-19, and requiring oxygen support or mechanical ventilation while admitted. Patients were PSM using logistic regression with 1:1 matching for binary long COVID status with exact matching on sex, race/ethnicity, COVID-19 variant period, and data-contributing site and nearest neighbor PSM on age. eGFR decline is assessed using the earliest post-COVID-19 eGFR measurement after infection (>= 30 days) and the last post-COVID-19 eGFR measurement taken within 365 days. These measurements must be at least 90 days apart. A decrease of >=20% is used as the threshold for eGFR decline. Abbreviations: hazard ratio (HR), subdistribution hazard ratio (sHR), Coronavirus disease 2019 (COVID-19), acute respiratory syndrome coronavirus (SARS-CoV-2), chronic kidney disease (CKD), end-stage kidney disease (ESKD), acute kidney injury (AKI).

Supplementary Methods Figure

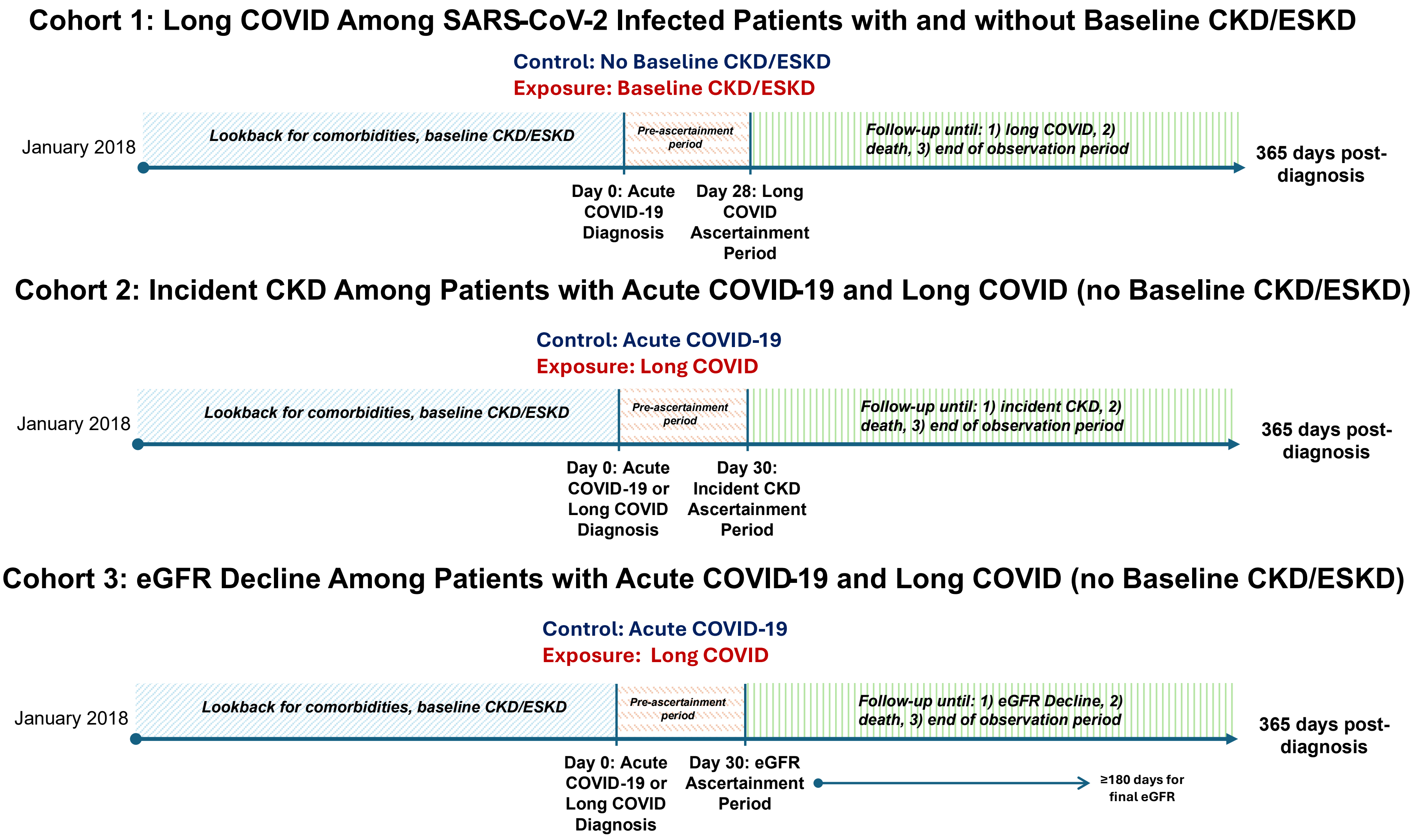

Supplementary Figure 1. Competing Risk for Long COVID/Death

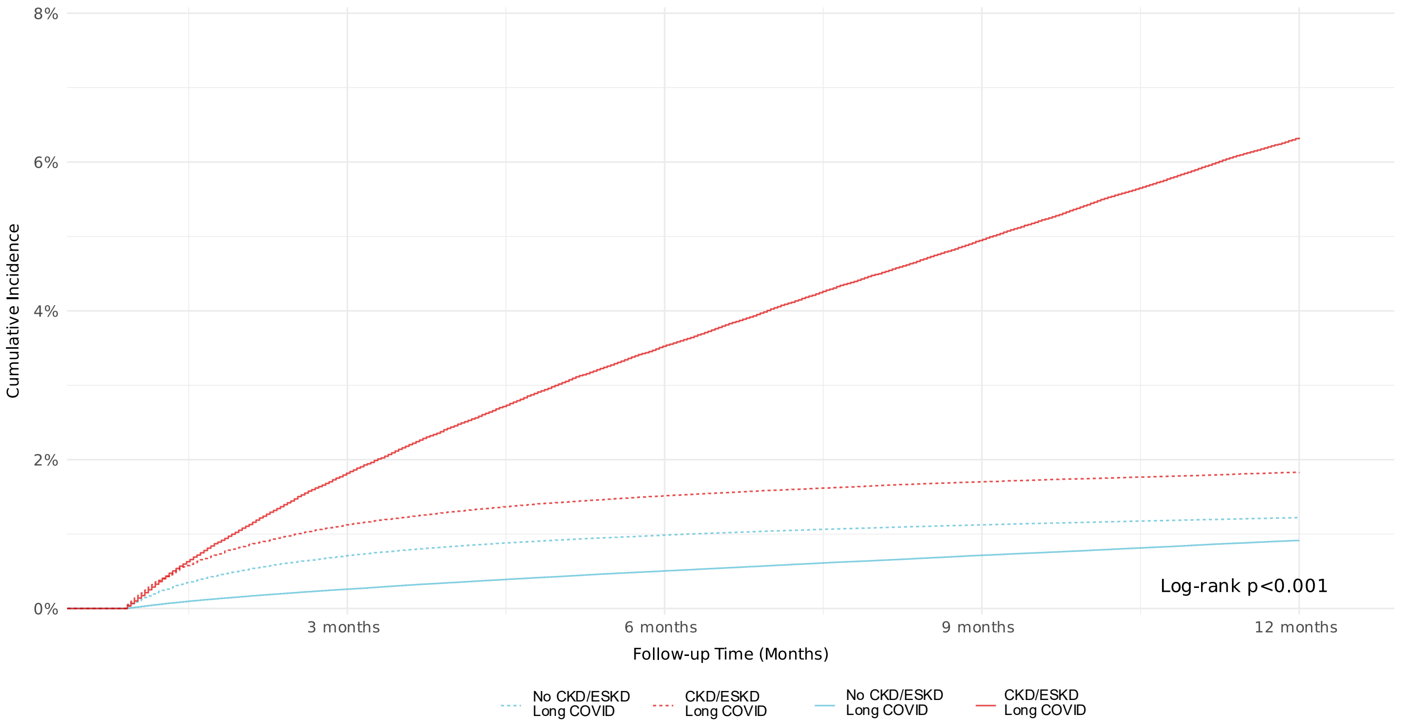

No CKD/ESKD				
At Risk	2000937	1966096	1928093	1867641
Events	19679	30148	37028	42837
CKD/ESKD				
At Risk	340403	326788	313380	296727
Events	10381	17671	23147	28133

Abbreviations: Coronavirus disease (COVID), chronic kidney disease (CKD), end-stage kidney disease (ESKD)

Supplementary Figure 2. Covariate Balance Plot After Matching for Long COVID as an Outcome

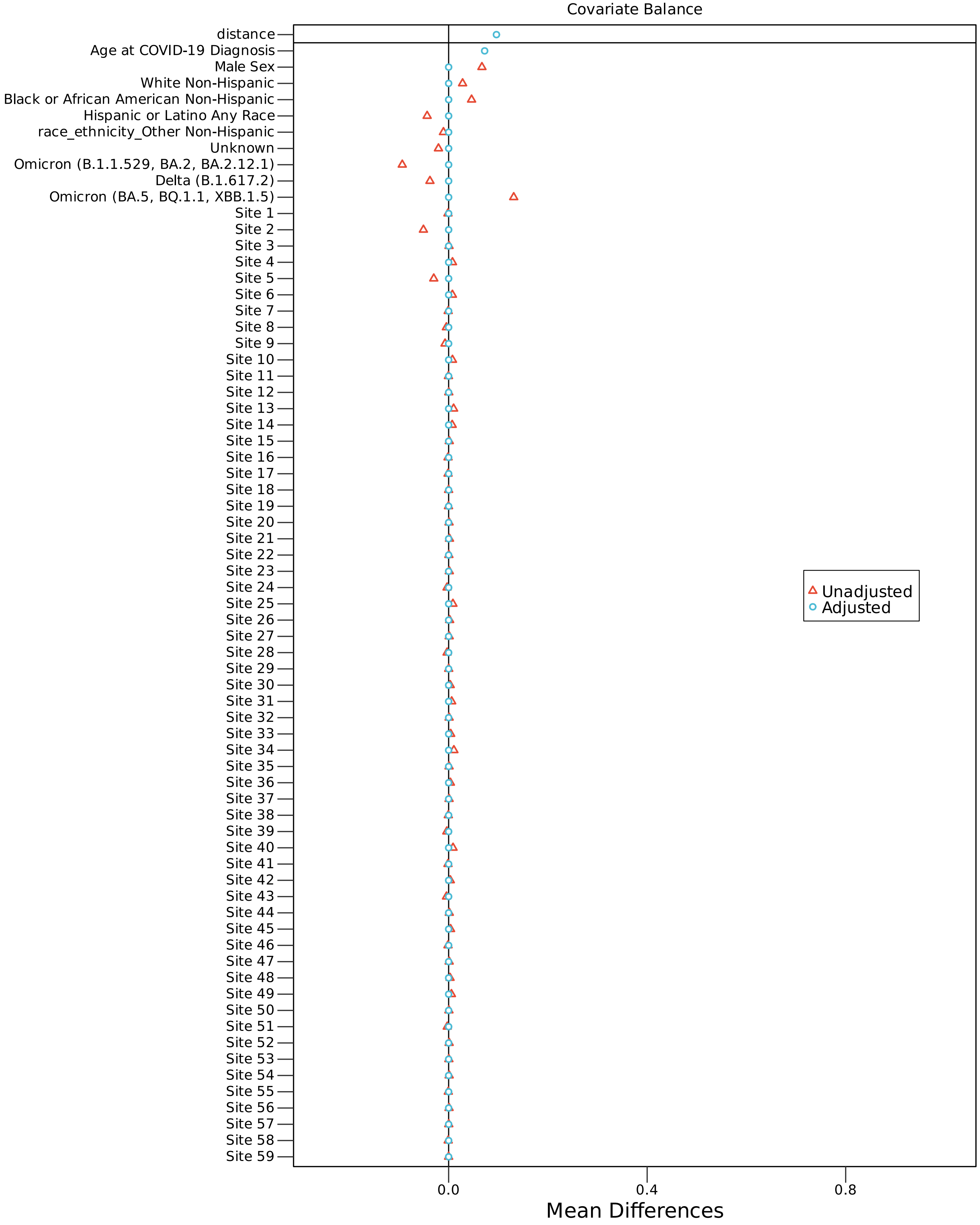

Supplementary Figure 3. Competing Risk for Long COVID/Death after PSM

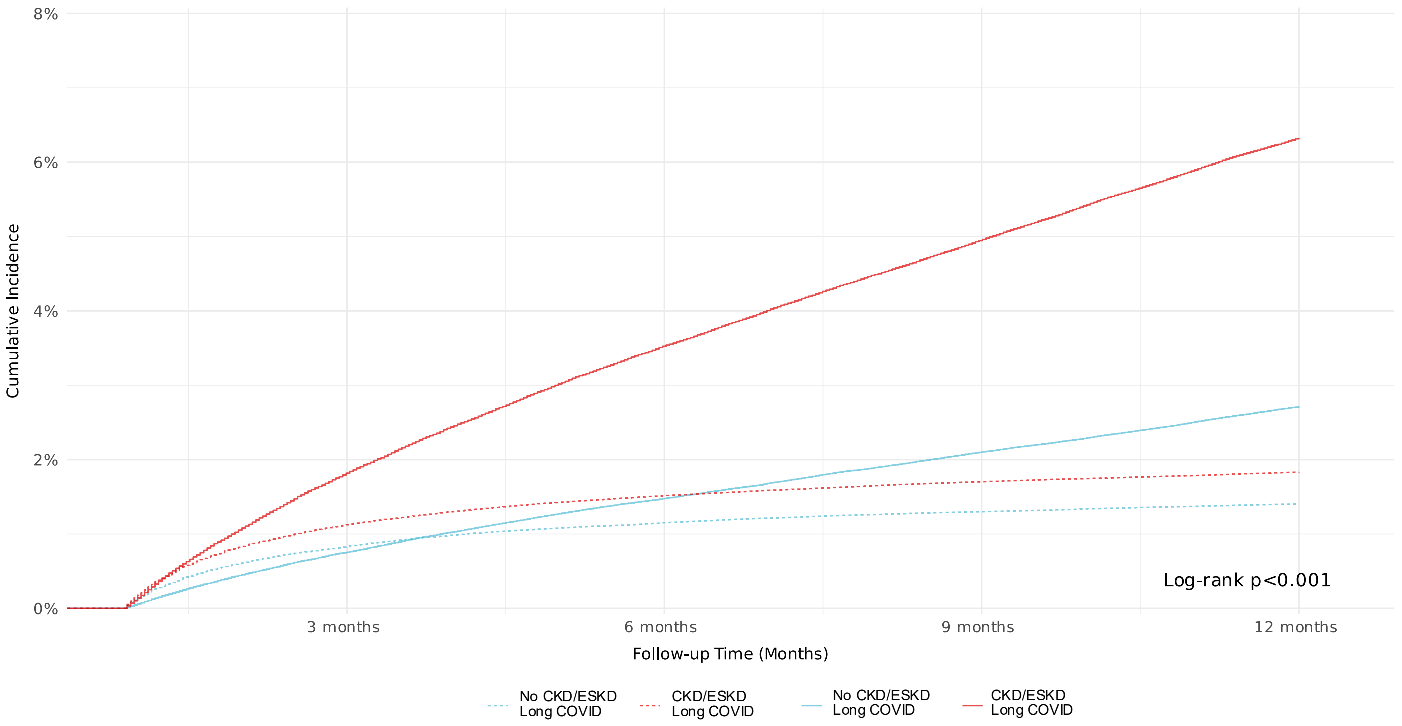

	3 months	6 months	9 months	12 months
**No CKD/ESKD**				
At Risk	345606	335910	325129	310132
Events	5594	9227	11856	14229
**CKD/ESKD**				
At Risk	340389	326777	313371	296720
Events	10381	17671	23147	28133

Abbreviations: Coronavirus disease (COVID), chronic kidney disease (CKD), end-stage kidney disease (ESKD), propensity score matching (PSM).

Supplementary Figure 4. Cumulative Incidence for Long COVID Among Patients with and without a History of CKD Stage 3a

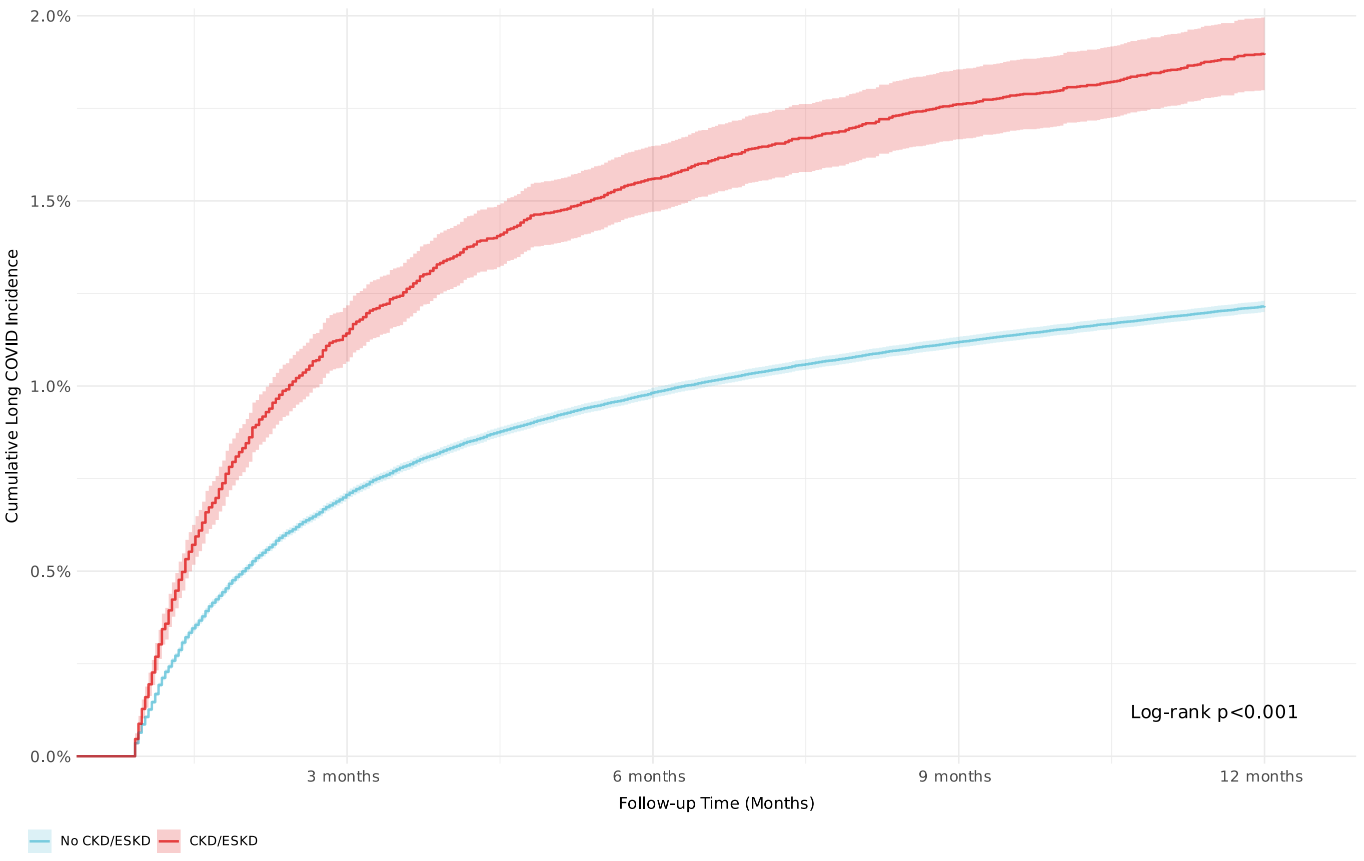

At Risk (Events)

No CKD/ESKD	1995796 (14250)	1965853 (19760)	1931946 (22455)	1875176 (24342)
CKD/ESKD	73858 (857)	72424 (1167)	70648 (1314)	67943 (1411)

Abbreviations: Coronavirus disease (COVID), chronic kidney disease (CKD)

Supplementary Figure 5. Cumulative Incidence for Long COVID Among Patients with and without a History of CKD Stage 3a after PSM

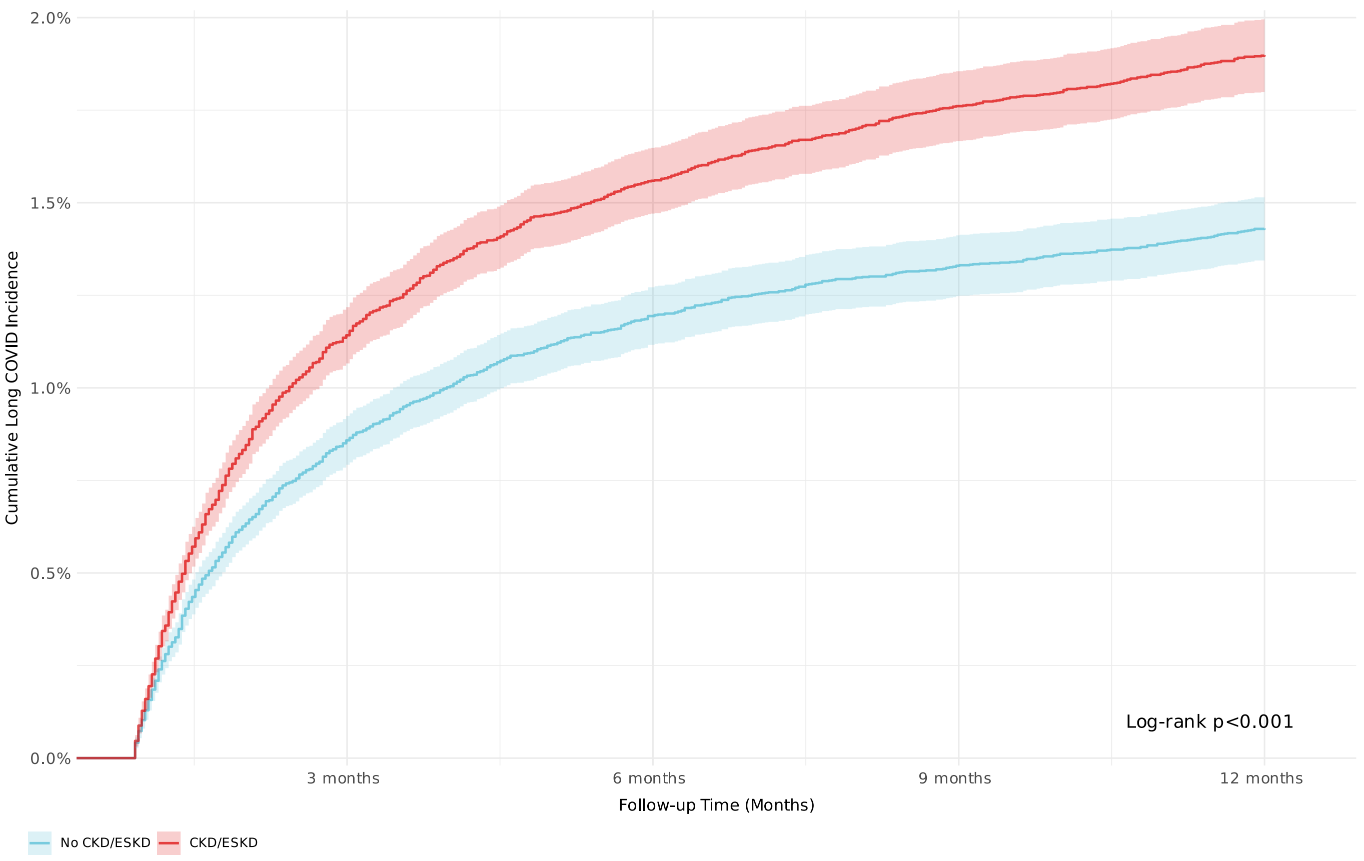

At Risk (Events)

	3 months	6 months	9 months	12 months
No CKD/ESKD	74044 (644)	72564 (893)	70850 (992)	68188 (1063)
CKD/ESKD	73856 (857)	72422 (1167)	70646 (1314)	67941 (1411)

Abbreviations: Coronavirus disease (COVID), chronic kidney disease (CKD), propensity score matching (PSM).

Supplementary Figure 6. Competing Risk for Incident CKD/ESKD and Death

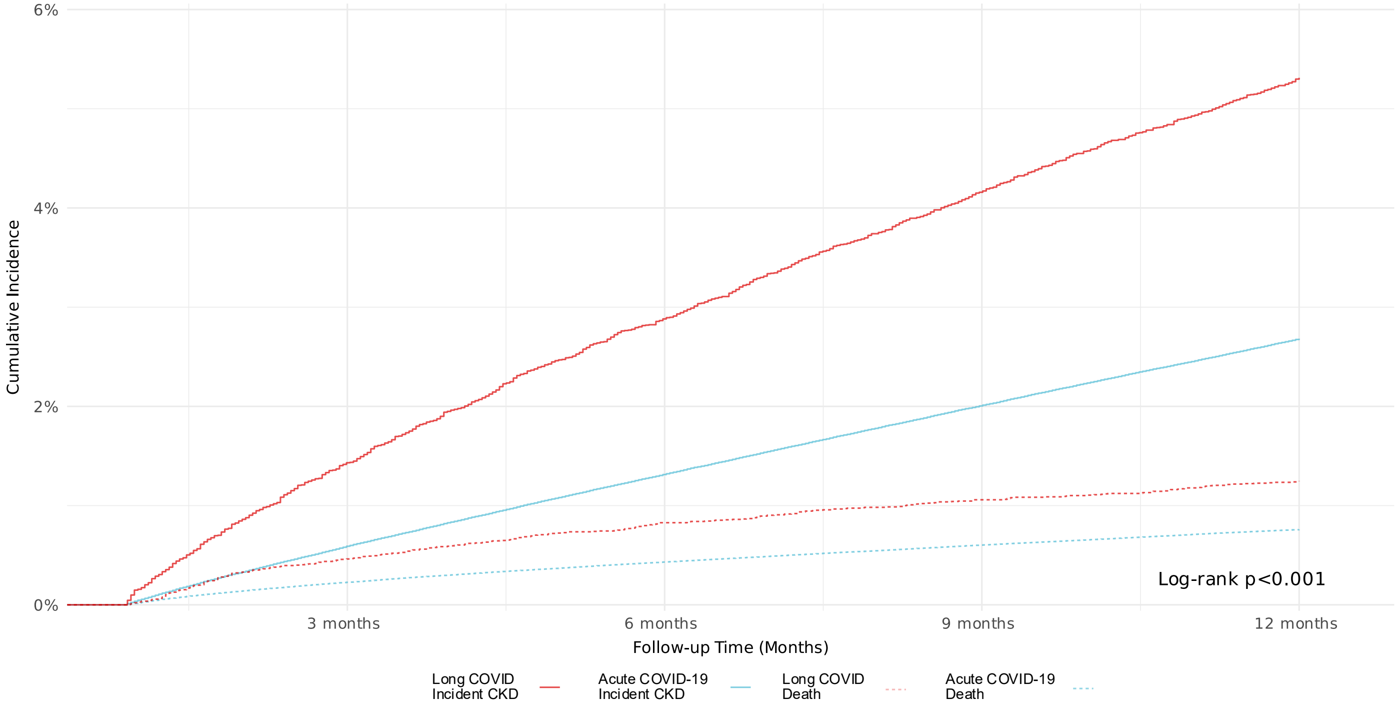

Acute COVID-19				
At Risk	1968899	1926824	1879880	1811250
Events	16286	34571	51374	67266
Long COVID				
At Risk	23715	23058	22377	21493
Events	459	896	1255	1568

Abbreviations: Coronavirus disease 2019 (COVID-19), chronic kidney disease (CKD), end-stage kidney disease (ESKD)

Supplementary Figure 7. Covariate Balance Plot After Matching for Incident CKD as an Outcome

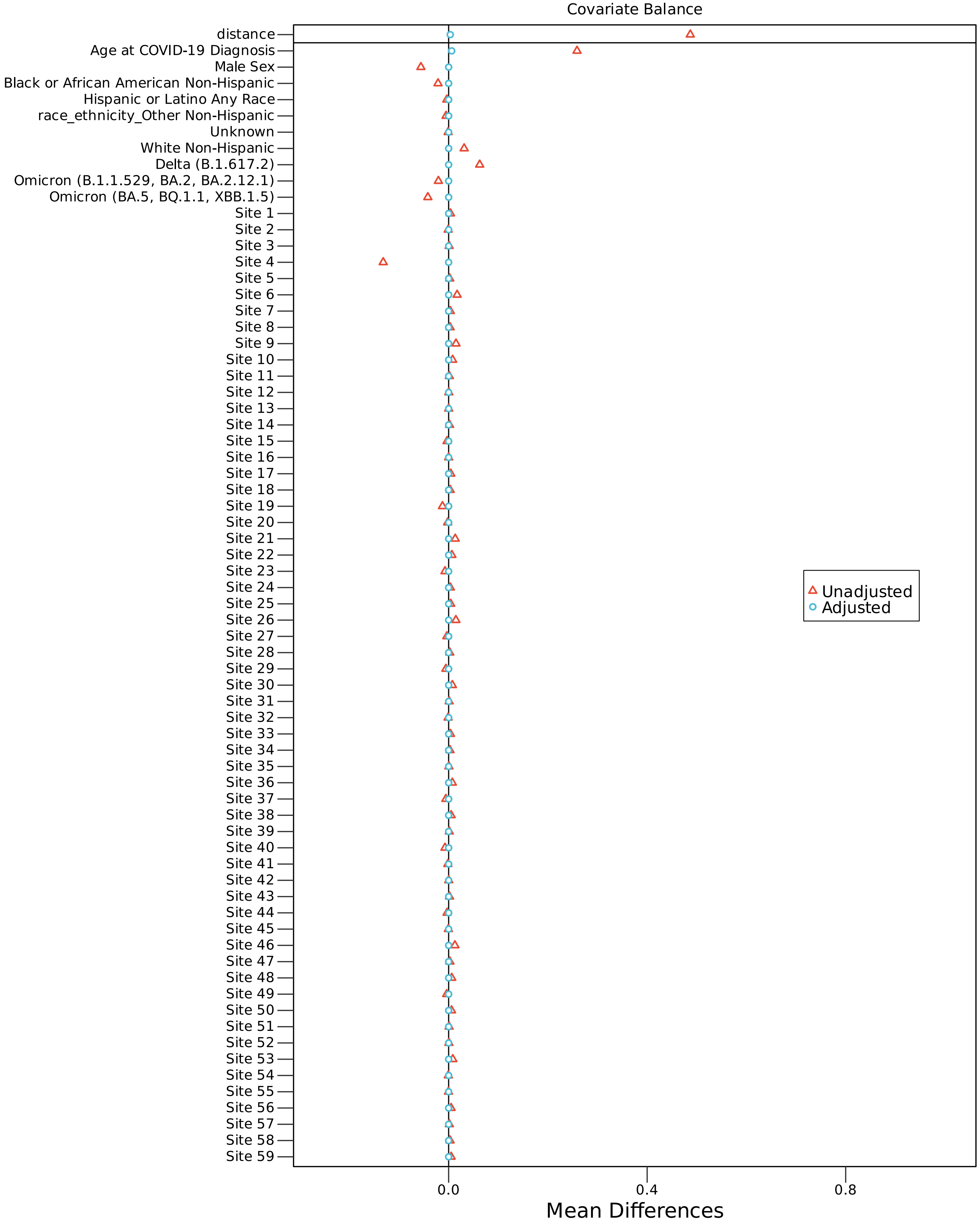

Supplementary Figure 8. Competing Risk for Incident CKD/ESKD and Death After PSM

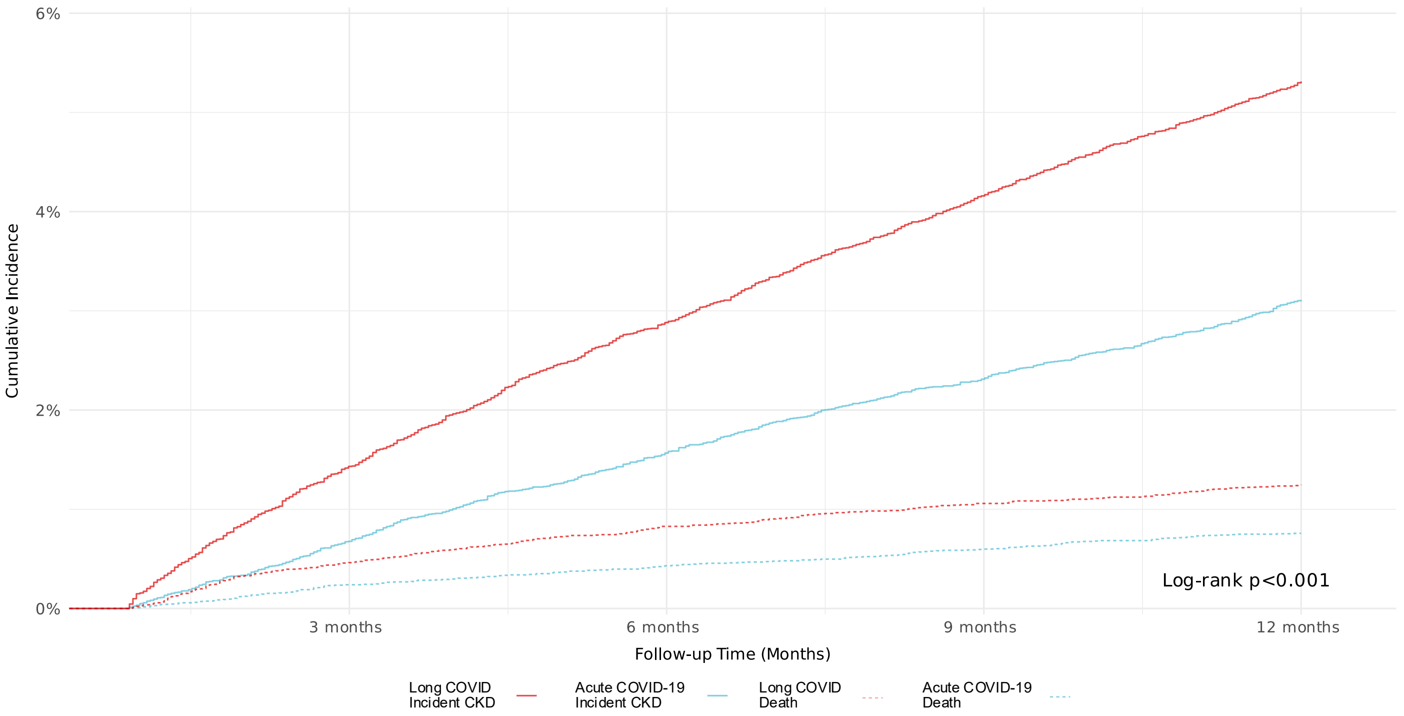

	3 months	6 months	9 months	12 months
**Acute COVID-19**				
At Risk	23949	23428	22899	22036
Events	222	482	698	922
**Long COVID**				
At Risk	23714	23057	22376	21492
Events	459	896	1255	1568

Abbreviations: Coronavirus disease 2019 (COVID-19), chronic kidney disease (CKD), end-stage kidney disease (ESKD), propensity score matching (PSM).

Supplementary Figure 9. Cumulative Incidence of eGFR Decline

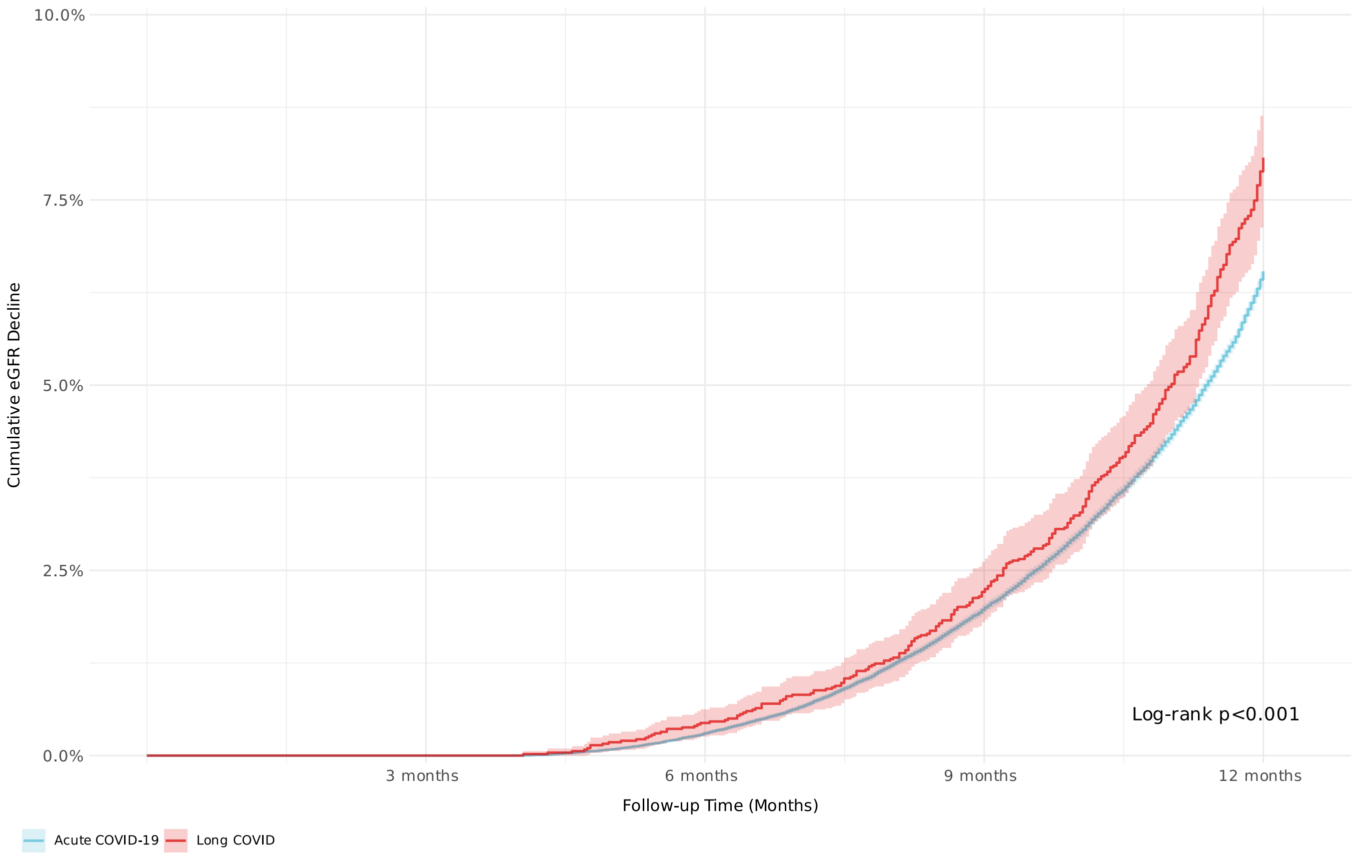

At Risk (Events)				
Acute COVID-19	207473 (0)	206581 (619)	201281 (4062)	186148 (13275)
Long COVID	4997 (0)	4972 (22)	4843 (110)	4446 (396)

Abbreviations: Coronavirus disease 2019 (COVID-19), estimated glomerular filtration rate (eGFR).

Supplementary Figure 10. Competing Risk for eGFR Decline and Death

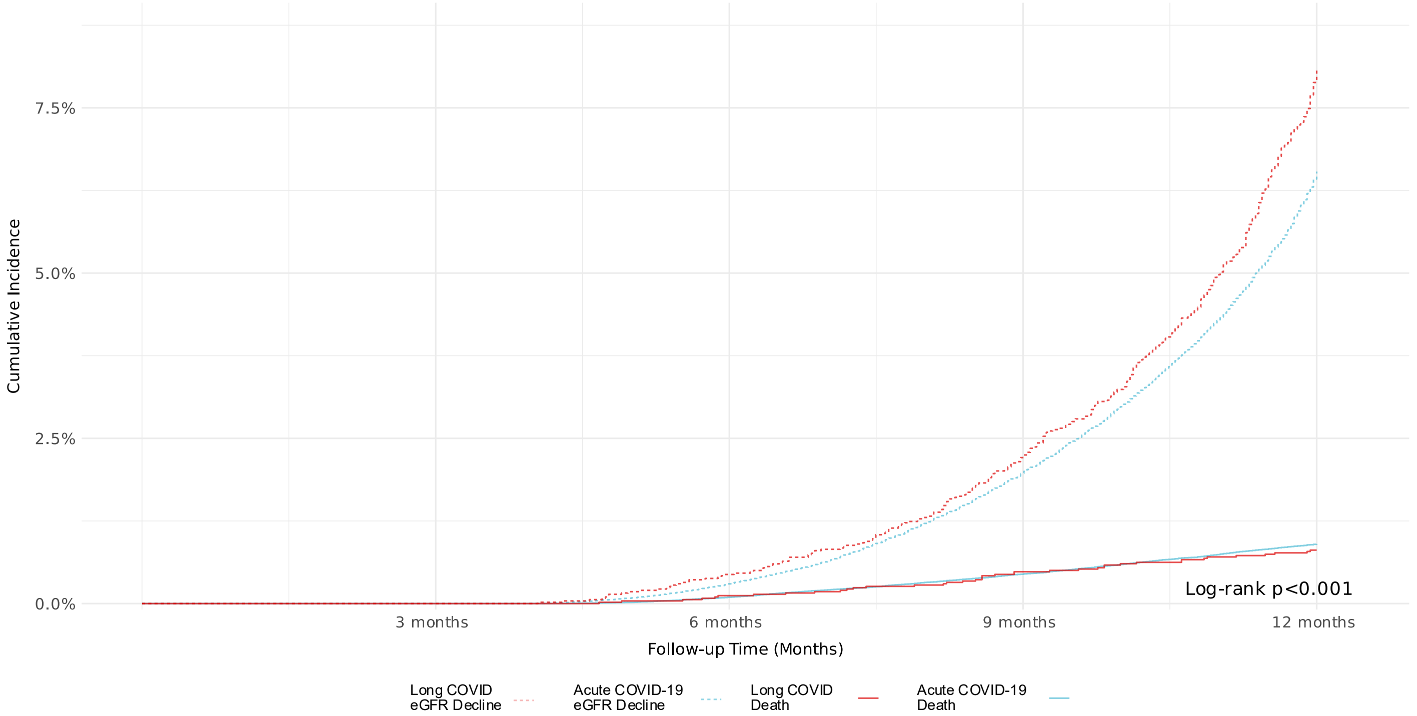

**Acute COVID-19**				
At Risk	207473	206384	200375	184390
Events	0	817	4980	15119
**Long COVID**				
At Risk	4997	4966	4820	4407
Events	0	28	134	436

Abbreviations: Coronavirus disease 2019 (COVID-19), estimated glomerular filtration rate (eGFR).

Supplementary Figure 11. Covariate Balance Plot After Matching for eGFR Decline as an Outcome

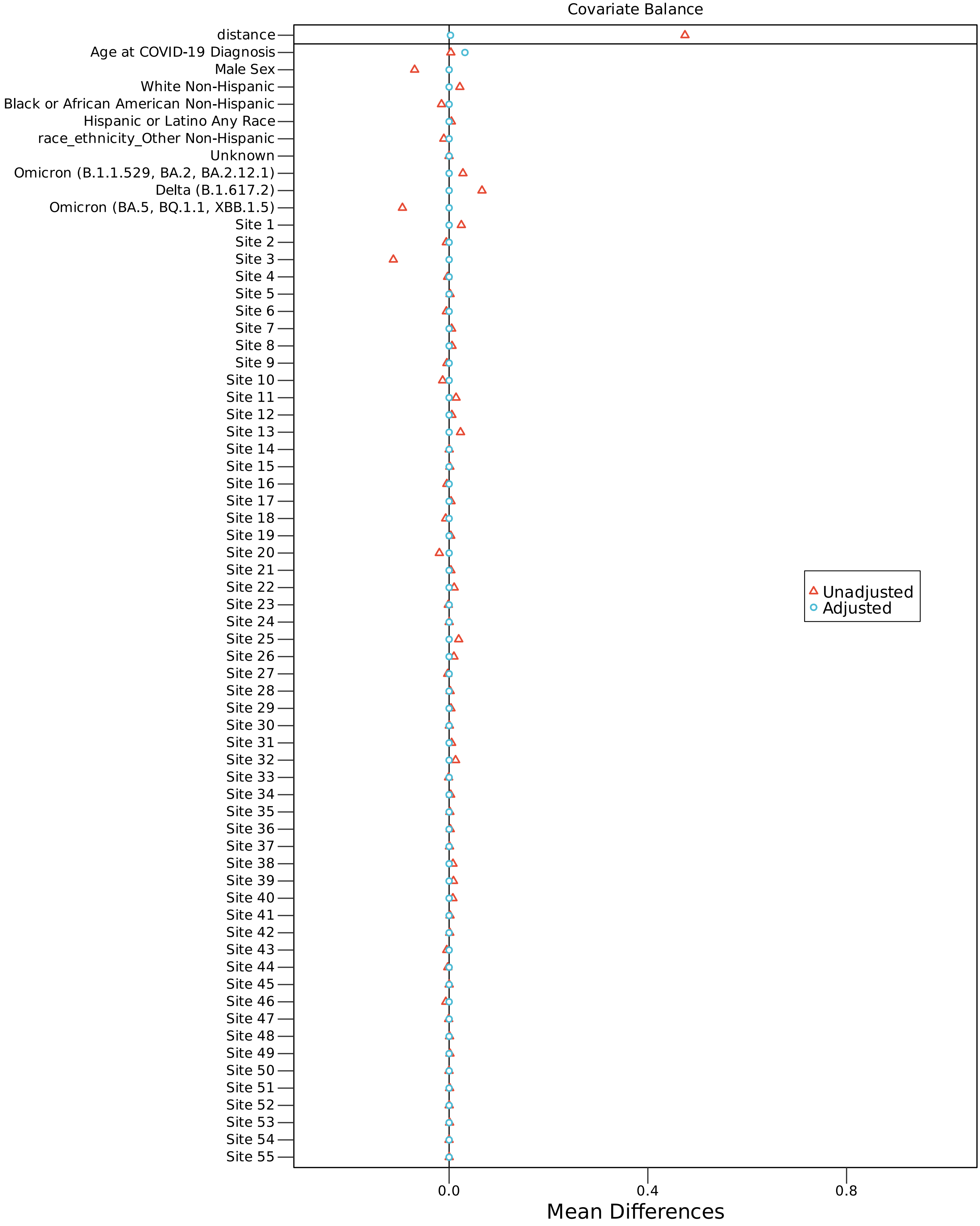

Supplementary Figure 12. Cumulative Incidence of eGFR Decline After PSM

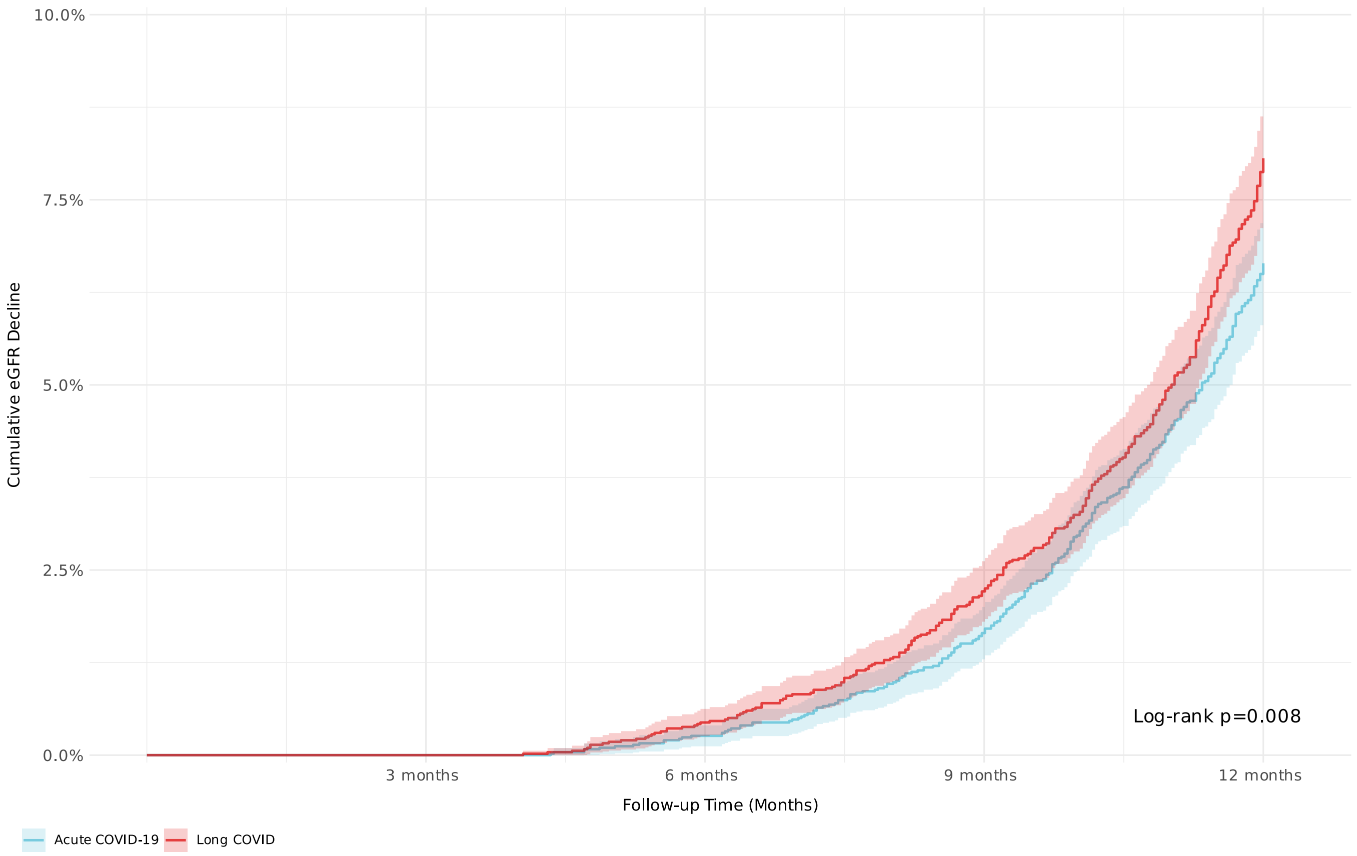

At Risk (Events)				
Acute COVID-19	4990 (0)	<4980(<20)*	4873 (82)	4513 (326)
Long COVID	4990 (0)	4965 (22)	4836 (110)	4440 (395)

*Small cell counts <20 are obfuscated to comply with N3C privacy policies
Abbreviations: Coronavirus disease 2019 (COVID-19), estimated glomerular filtration rate (eGFR), propensity score matching (PSM).

Supplementary Figure 13. Competing Risk for eGFR Decline and Death After PSM

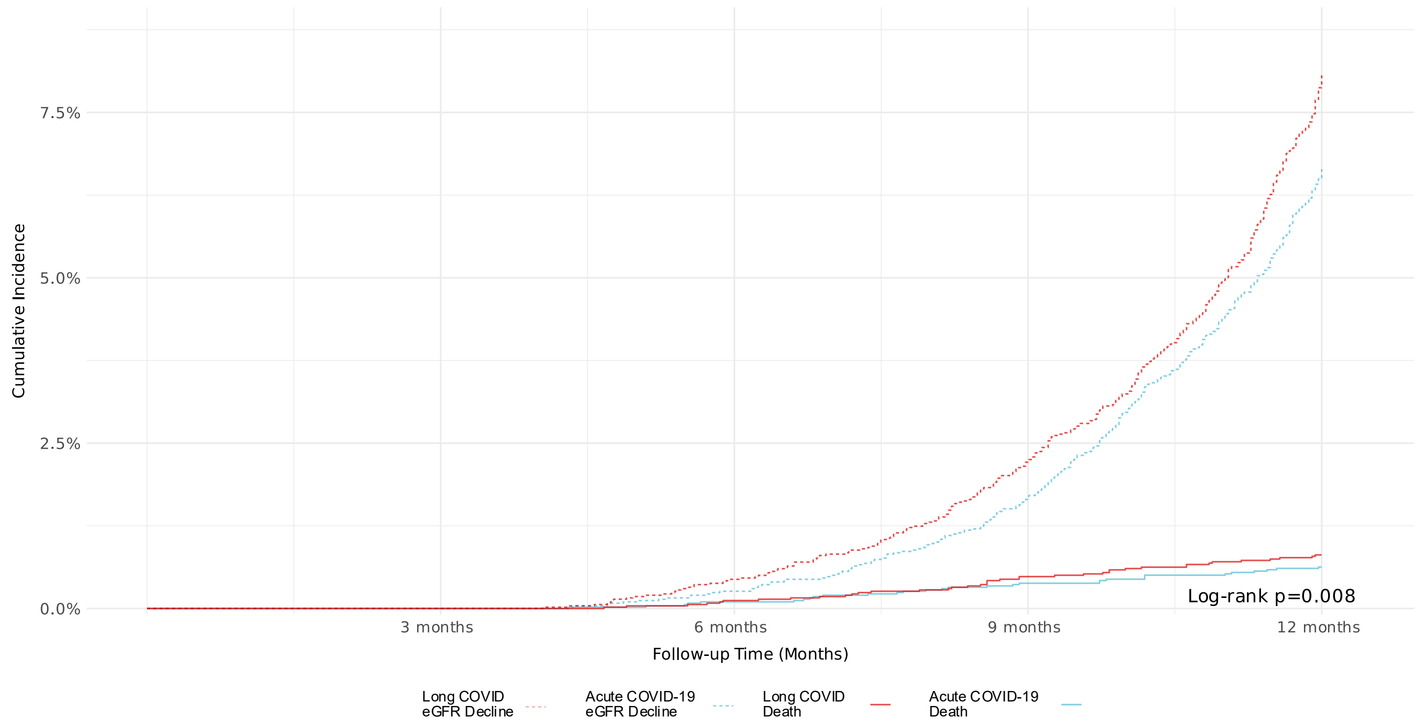

**Acute COVID-19**				
At Risk	4990	4965	4855	4483
Events	0	<20*	101	357
**Long COVID**				
At Risk	4990	4959	4813	4401
Events	0	28	134	435

*Small cell counts <20 are obfuscated to comply with N3C privacy policies
Abbreviations: Coronavirus disease 2019 (COVID-19), estimated glomerular filtration rate (eGFR), propensity score matching (PSM).
